# Prescribed drugs containing nitrogen heterocycles: an overview

**DOI:** 10.1039/d0ra09198g

**Published:** 2020-12-15

**Authors:** Majid M. Heravi, Vahideh Zadsirjan

**Affiliations:** Department of Chemistry, School of Science, Alzahra University PO Box 1993891176, Vanak Tehran Iran mmh1331@yahoo.com mmheravi@alzahra.ac.ir +98 21 88041344 +98 21 88044051

## Abstract

Heteroatoms as well as heterocyclic scaffolds are frequently present as the common cores in a plethora of active pharmaceuticals natural products. Statistically, more than 85% of all biologically active compounds are heterocycles or comprise a heterocycle and most frequently, nitrogen heterocycles as a backbone in their complex structures. These facts disclose and emphasize the vital role of heterocycles in modern drug design and drug discovery. In this review, we try to present a comprehensive overview of top prescribed drugs containing nitrogen heterocycles, describing their pharmacological properties, medical applications and their selected synthetic pathways. It is worth mentioning that the reported examples are actually limited to current top selling drugs, being or containing N-heterocycles and their synthetic information has been extracted from both scientific journals and the wider patent literature.

## Abstract 2

In this review, we try to present a comprehensive overview on top prescribed drugs containing nitrogen heterocycles, describing their pharmacological properties, medical applications and their selected synthetic pathways.

## Graphical abstract

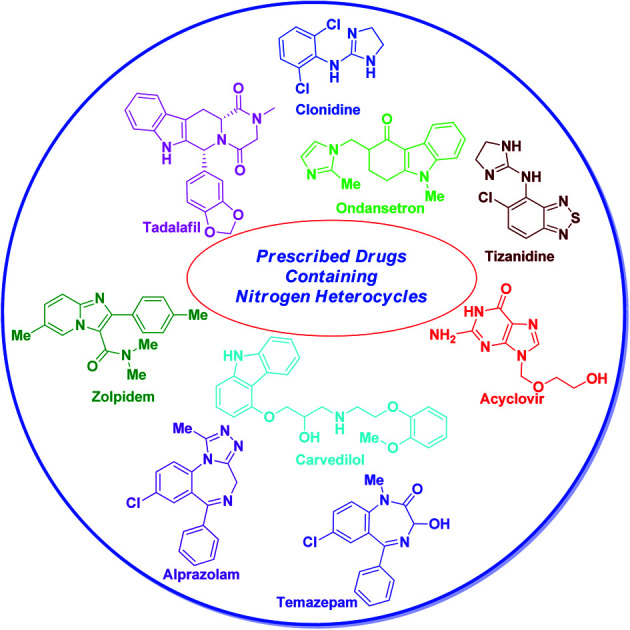

## 1. Introduction

Medicinal and pharmaceutical chemistry are disciplines at the intersection of chemistry, especially synthetic organic chemistry, and pharmacology and various other biological specialties, leading to the design, chemical synthesis and development of bio-active molecules, for being approved as prescribed and market purchasable pharmaceutical agents. Heterocyclic compounds, as the most important organic compounds, are frequently present in molecules of interest in medicinal chemistry.^1^ Among them, nitrogen containing heterocycles are of great importance to life science, since they are abundant in nature, existing as subunits in several natural products, for example vitamins, hormones and antibiotics. Some representative alkaloids and other nitrogen containing natural products, showing diverse biological activities, and several of them are even prescribed drugs such as serotonin,^2^ thiamine, which is also called vitamin B1,^3^ atropine,^4^ notorious morphine,^5^ codeine, (greater benefit may be gained when it is combined with acetaminophen or a nonsteroidal anti-inflammatory drug (NSAID) such as aspirin or ibuprofen),^6^ papaverine,^7^ coniine,^8^ caffeine^9^ and nicotine.^10^

Furthermore, N-based heterocycles are indispensable diet components such as thiamin (vitamin B1), riboflavin (vitamin B2), pyridoxol (vitamin B6), nicotinamide (vitamin B3).^11,12^ Nitrogen-containing heterocyclic compounds are not only present as the backbone in several biologically active natural products used as traditional medications or approved prescribed drugs, but some of their synthetic derivatives in different sizes, nowadays are prescribed and market purchasable drugs. The most famous are, diazepam, isoniazid, chlorpromazine, metronidazole, barbituric acid, captopril, chloroquinine, azidothymidine and *anti*-pyrine. Furthermore, most of the vitamins, nucleic acid, enzymes, co-enzymes, hormones, and alkaloids contain N-based heterocycles as scaffolds.^13^

Due to exhibiting diverse biological activities, nitrogen heterocyclic compounds have always been attractive targets to synthetic organic chemists. Since, several of them are prevalent in natural products, especially alkaloids, they have received much attention of synthetic community, especially those who are engaged with the total synthesis of natural products.^14^ As a result, the vast number of nitrogen heterocyclic compounds have been under continuous investigations from different points of view thus, found applications in pharmaceutical research and drug discovery.^15,16^ Recently, N-based heterocycles have attracted much interest of medicinal chemists and biologists due to broad range of biological activities and plentiful applications in the extensive fields of pharmacy.^17^

FDA databases has revealed that about 60% of unique small-molecule drugs, comprise N-based heterocycles, showing the structural significance of N-based heterocycles in drug design and drug discovery.^18^ The prevalence of N-heterocyles in biologically active compounds can be attributed to their stability and operational efficiency in human body and the fact of that the nitrogen atoms are readily bonded with DNA through hydrogen bonding. As a matter of fact, anti-cancer activities of N-based heterocycle agents are largely due to their tendency of interaction with DNA *via* hydrogen bonding.^19^

In 2014 Njardarson *et al.* published the first comprehensive analysis of the nitrogen based heterocycles.^16^ This analysis showed that indeed about 60% of small-molecule drugs contain a N-based heterocycle as common architectural cores. In 2011, Baumann *et al.* presented an overview of the key pathways to the synthesis of the best-selling five-membered ring heterocyclic medications regardless of their kinds of heterocycles.^20^ In the following, in 2013 the same authors presented an overview on the synthetic pathways to the best selling drugs comprising six-membered heterocyclic systems.^21^ In 2018, Ramazani and co-workers^15^ presented the recent advances in nitrogen-based heterocyles as useful cancer chemotherapy agents. Cancer is one of the foremost roots of death, globally. It is the result of mutation of the cells which regulate the genes and protein. Although, surgery and radiotherapy are the current therapy several drugs are also used as anticancer agents in spite of their undesired side effects. Some analogues of new isosteviol-fused pyrazoline, ursolic acid linked triazole or d-ribose linked exhibit anticancer activity at the nanomolar range.^22^ Furthermore showing segment resemblance with histidine imidazole molecule N-based-heterocycles can be linked with protein molecules more easily than some other heterocyclic scaffolds, thus, these types of N-heterocyclics are the most promising drugs for being designed and screened as anti-cancer drugs.^23^ We are interested in heterocyclic chemistry,^24^ especially those containing nitrogen atom.^25–33^

We are interested in heterocyclic chemistry,^24^ especially those containing nitrogen atom.^25–33^ In recent years, our group has also focused on the applications of name reactions in the total synthesis of natural products containing nitrogen heterocycles, showing diverse biological activities.^34–42^ Armed with these experiences, in this review we try to highlight the medical usages and selected synthetic pathways of approved and market purchasable prescribed medications, containing nitrogen based-heterocycles. Having collected and categorized of about 640 medications, comprising a based-nitrogen heterocycle, we had to be selective and sumarritive, limiting ourselves to most common of such pharmaceuticals, classifying them in accordance of their size of N-based heterocycles, in, four, five, six, and seven-membered rings. Moreover, the fused, bridged bicyclic nitrogen heterocycles have been also covered.

## 2. Synthesis of nitrogen prescribed drugs having

### 2.1 Four-membered heterocycles

In general antimicrobial drugs are recognized as bacteriostatic (*i.e.*, tetracyclines, sulfonamides) and as antibacterial (*i.e.*, penicillin). Beta-lactam antibiotics are categorized to four groups. They are penicllins, cephalosporins, monobactams, and carbapenems. They all comprise a four-membered beta-lactam ring that is essential for displaying their antibactericidal activities. In 1929, penicillin was explored by Sir Alexander Fleming, who observed that one of his experimental cultures of staphylococcus was polluted with fungus that caused the bacteria to lyse.^43,44^ Since fungus belonged to the family penicillium, he called this bactericidal substance penicillin. A decade later, a research group at Oxford University could isolated a crude substance built of a few low-molecular substances that were named penicillins (F, G, K, O, V, X). Among the various penicillins (F, G, K, O, V, X), penicillin G (benzylpenicillin), was found the most effective. Since then, penicillin G, is used as an antibiotic to treat a number of bacterial infections.^45^

**Scheme 1:**
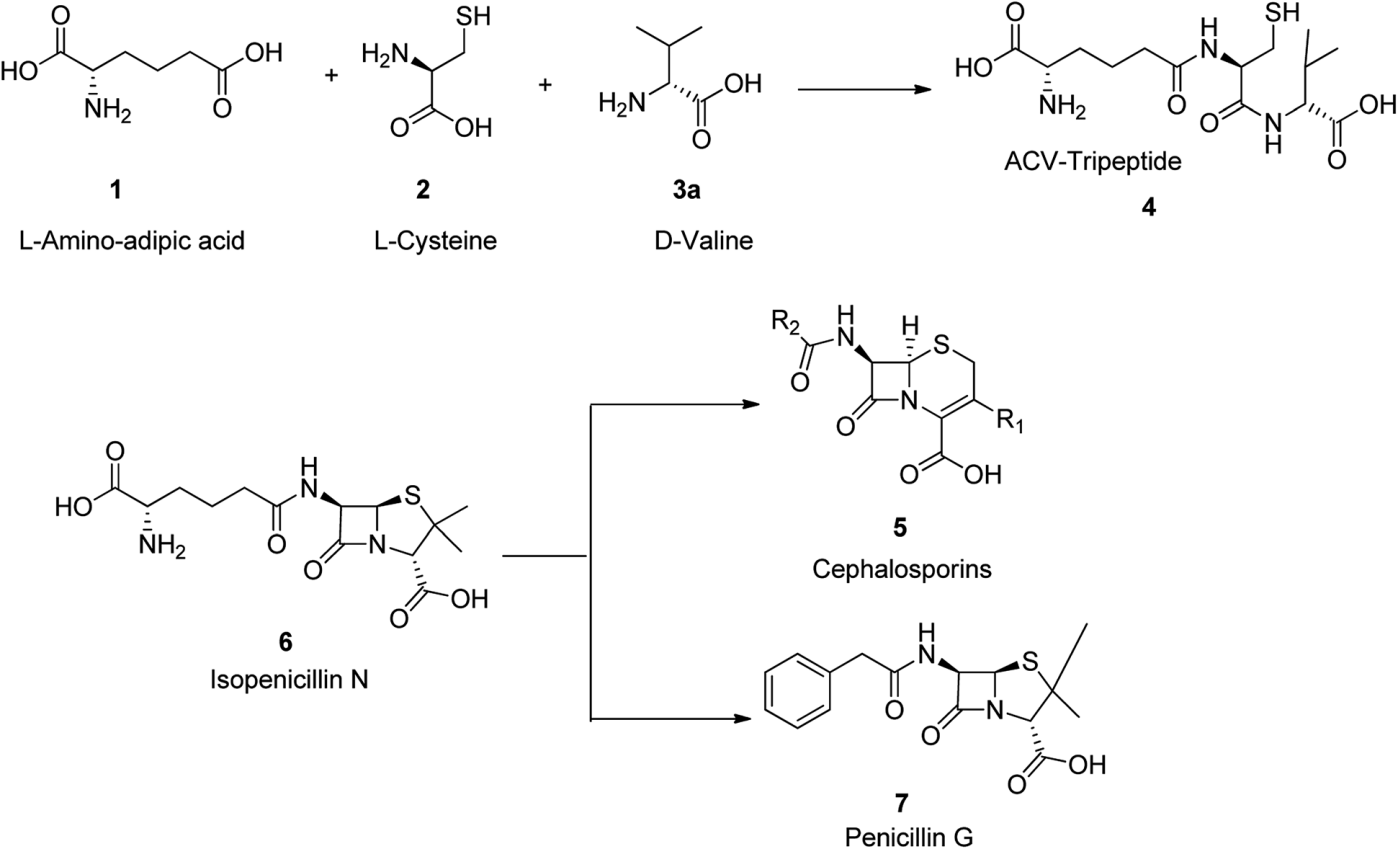
Synthesis of penicilin G 7.

There are three main and remarkable stages to the biosynthesis of penicillin G 7 (benzylpenicillin). Initially, three amino acids-l-α-aminoadipic acids, l-cysteine, l-valine are condensed to a tripeptide.^46–48^ Before the generation of this tripeptide, the amino acid l-valine is subjected to epimerization to turn out to be d-valine 3.^49,50^ This tripeptide is called δ-(l-α-aminoadipyl)-l-cysteine-d-valine (ACV) 4. The above epimerization and condensation reaction both are catalyzed by the enzyme δ-(l-α-aminoadipyl)-l-cysteine-d-valine synthetase (ACVS), a nonribosomal peptide synthetase or NRPS. In the second step of biosynthetic process of penicillin G 7, the catalyzed-isopenicillin N synthase (IPNS) oxidative transformation of linear ACV into the bicyclic intermediate isopenicillin N is taken place.^46–49^ Ultimately, by isopenicillin N 6, *N*-acyltransferase, is *trans* amidated in a way that the α-aminoadipyl side-chain of isopenicillin N 6 is eliminated and replaced by a phenylacetyl side-chain. This process is encoded by the gene *pen*DE and considered as exceptional progression in providing penicillins G 7 (Scheme 1).^46^

**Scheme 2:**
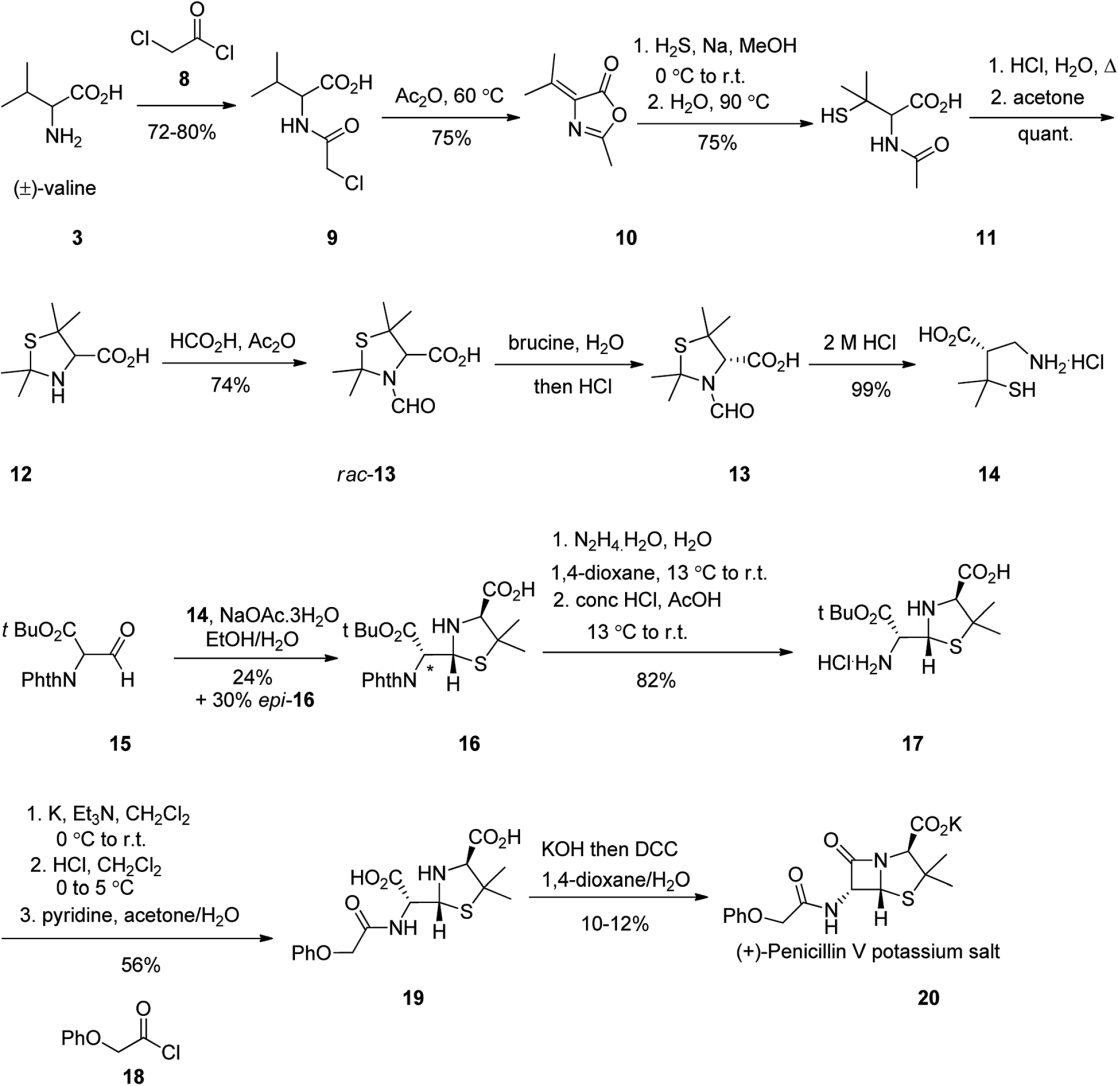
Synthesis of penicillin V 20.

The total synthesis of penicillin V 20 was first achieved in 1948. It started with racemic valine 3, which was effectively converted into *N*-acetylpenicillamine 11. Formamide *rac*-13 upon resolution using brucine followed by hydrolysis, gave (−)-penicillamine hydrochloride 14. The latter was condensed with aldehyde 15 to give thiazolidine 16. The side-product *epi*-16 could be transformed into 16 using pyridine-induced epimerization. Elimination of protecting groups and assemblage of the phenoxyacetyl side chain provided penicilloic acid 19. Successive creation of the central amide bond was accomplished using DCC in basic conditions to afford penicillin V 20 as its potassium salt (Scheme 2).^51^

**Scheme 3:**
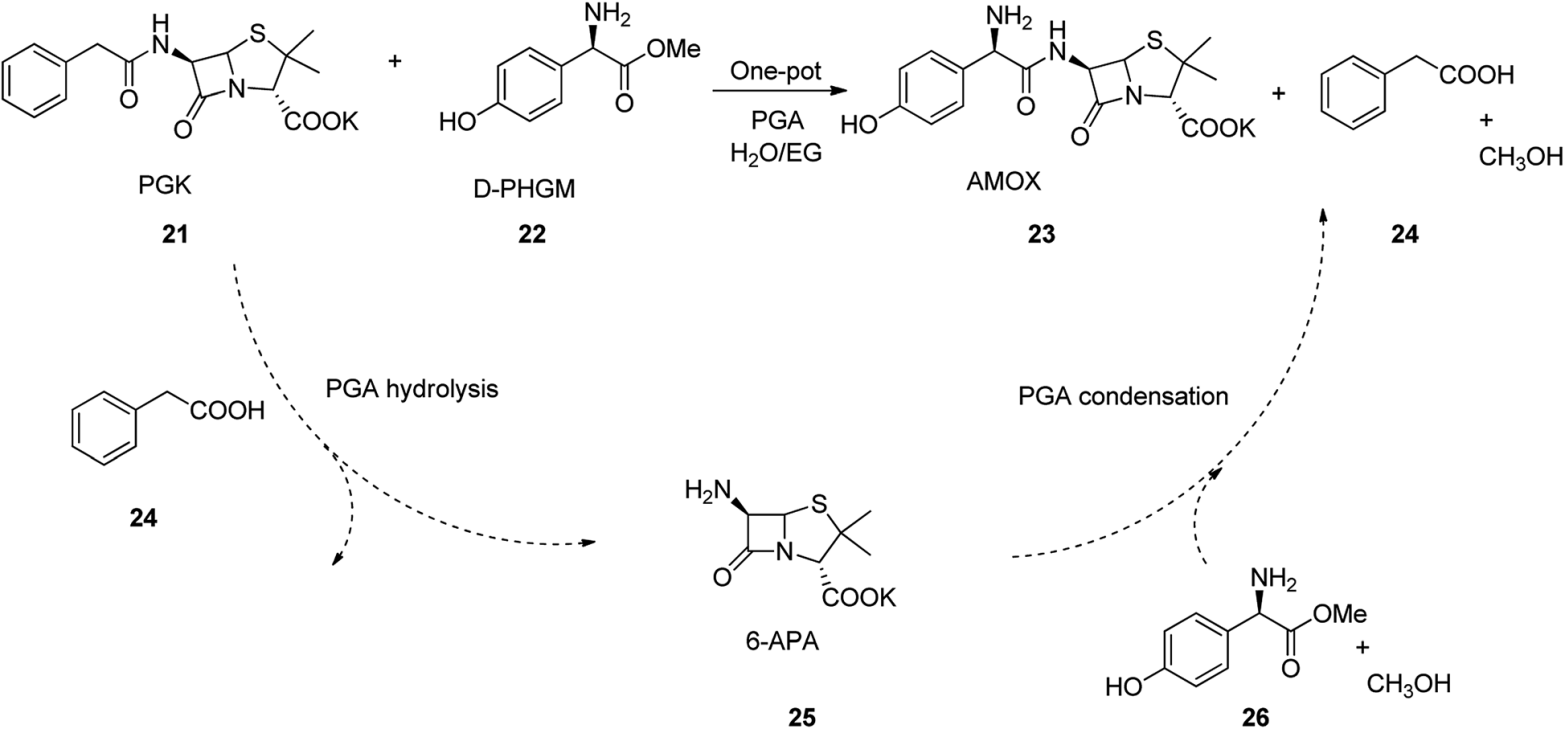
Two-step one-pot enzymatic cascade process for industrial synthesis of amoxicillin.

Amoxicillin is an antibiotic employed for the treatment of several bacterial infections, involving, strep throat, pneumonia skin infections middle ear infection, and urinary tract infections *etc.*^45,52^ Amoxicillin is one of the major β-lactam and best-selling antibiotics. It was discovered in 1958 and came into medical use in 1972 with much advantage over its precedents, for example it show higher spectrum of potency, high solubility, and high rate of absorption.^53,54^ Amoxicillin can also be prepared by enzymatic one-pot approach which has significant imminent application in its large scale production. The process began with 6-aminopenicillanic acid (6-APA) 25, which initially activated by a substrate, such as *p*-hydroxyphenylglycine methyl ester (HPGM) or *p*-hydroxyphenylglycine amide. It is well-recognized that PGA not only convert such substrates into an antibiotic, but also hydrolyzes penicillin G potassium salt (PGK) 21 into 6-APA 25. As a matter of fact, most of the β-lactam nuclei, *e.g.*, 6-APA 25 and 7-ADCA employed in the enzymatic semi-synthetic process of β-lactam antibiotics are provided from the hydrolysis of PGK or cephalosporin C mediated by PGA. Thus, combination of the hydrolysis of PGK into 6-APA with the enzymatic catalysis is resulted in coupling reaction of 6-APA with *p*-hydroxyphenylglycine methyl ester (D-HPGM) to afford amoxicillin as the desired product. This one-pot approach avoids the number of steps in the production of β-lactam antibiotic, which not only skipping the isolation of 6-APA 25, but also effectively decrease the industrial cost production (Scheme 3).

**Scheme 4:**
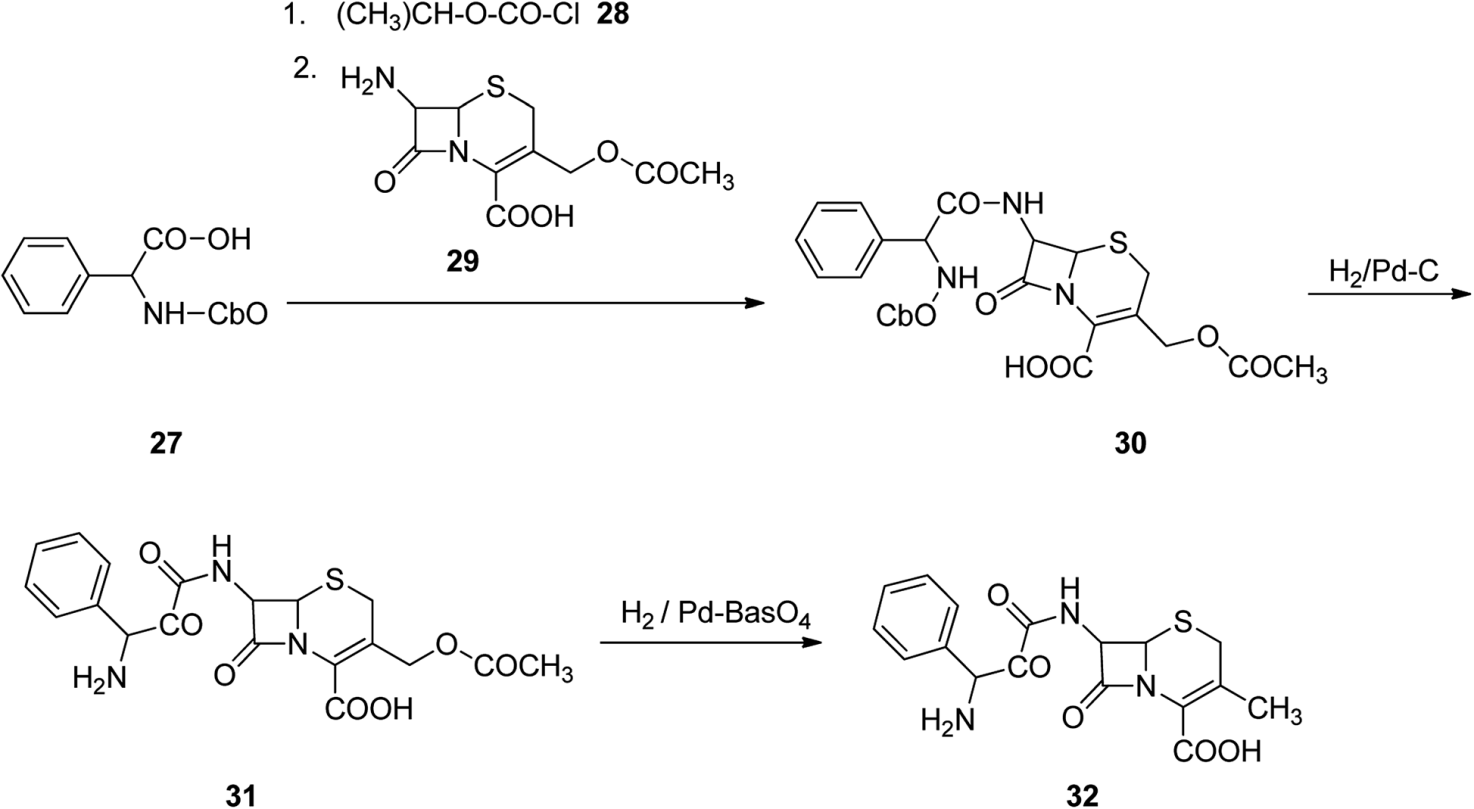
Synthesis of cephalexin 32.

Cefalexin, is an antibiotic used for the treatment of several bacterial infections.^55^ Cefalexin used for treatment of definite bacterial infections, involving those grown of the middle ear, bone and joint, skin, and urinary tract, pneumonia, strep throat and to prevent bacterial endocarditis. Cefalexin was discovered in 1967.^56–58^ Initially, it was promoted in 1969 and 1970 under the brand names Keflex and Ceporex.^59^ Cefalexin under generic versions and under other trade names are, inexpensively market purchasable.^60^ Cephalexin is a first-generation cephalosporin antibiotic that was selected as the model medicine nominee to attain dose with better stability, palatability and attractive pediatric sophistication, economic and easy to take.^61–68^ Cephalexin, [6*R*-[6α,7β(*R*)]]-3-methyl-8-oxo-7-[(aminophenylacetyl)amino]-5-thia-1-azabicyclo[4.2.0]oct-2-en-2-carboxylic acid 32, indeed is an analog of ampicillin, due to the acyl segment present in the structure of 7-aminocephalosporanic acid, is just the same phenylglycine segment present in ampicillin.^69^ Cephalexin 32 is provided from cephalophenylglycine 31 that is in turn prepared by treating 7-aminocephalosporanic acid with a mixed anhydride which itself synthesized upon treatment of *N*-carbobenzoxyphenylglycine and isobutyl chloroformate in the presence of Et_3_N. Removal of the *N*-carbobenzoxy protective moiety from the obtained product 30*via* hydrogentation in the presence of Pd on carbon catalyst provided a cephalophenyl-glycine 31 as an internal salt. Hydrogenation of the latter in the presence of Pd on barium sulfate leads to the deacetoxylation at the third position of 7-aminocephalosporanic acid, producing the desired prescribed antibiotic, cephalexin 32 (Scheme 4).^70–72^

**Scheme 5:**
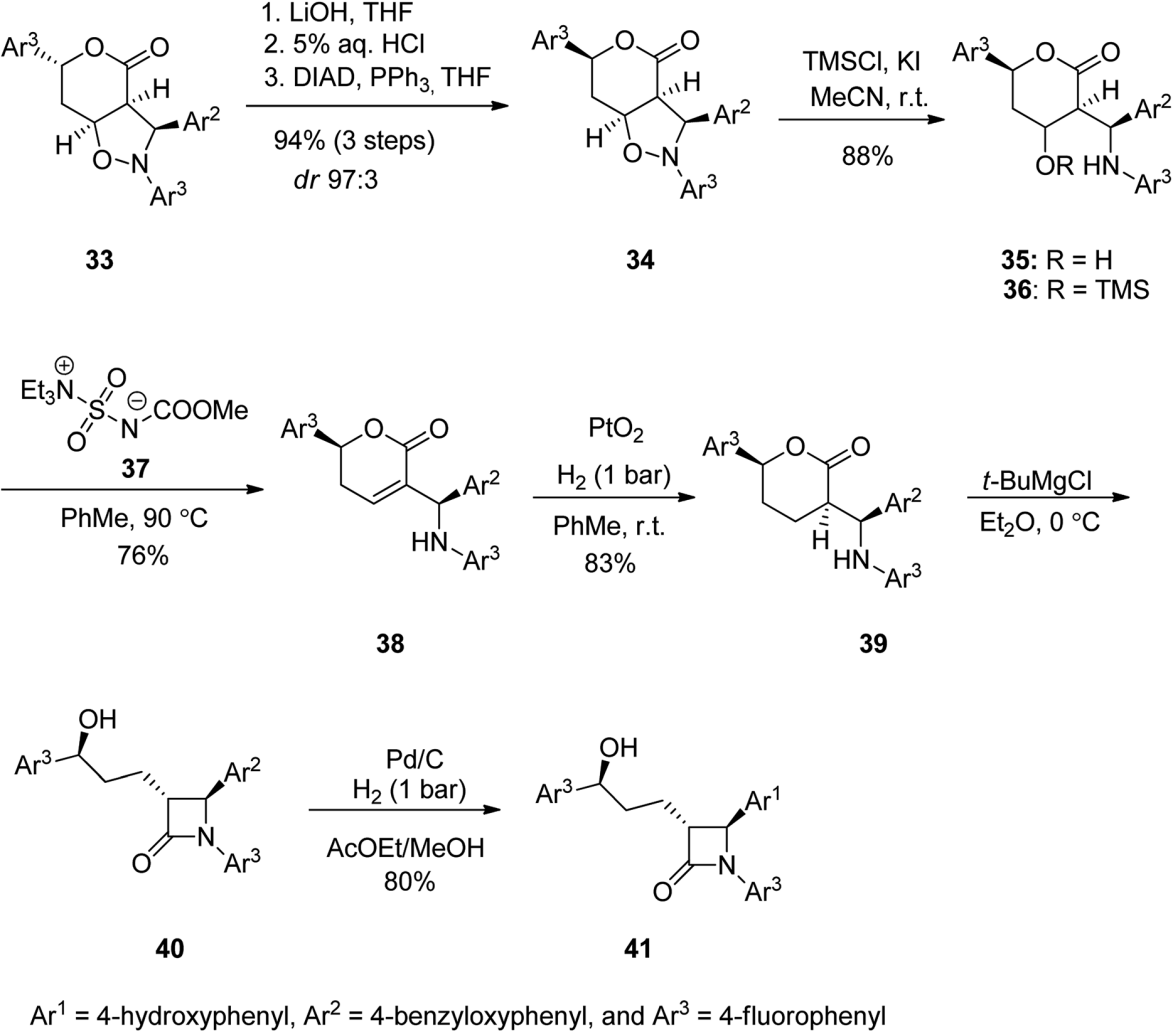
Synthesis of ezetimibe 41.

Ezetimibe is a medicine employed for the treatment of high blood cholesterol and some other lipid abnormalities. Ezetimibe was approved for medical use in the United States in 2002.^73,74^ Ezetimibe 41 is a strong β-lactamic cholesterol absorption inhibitor that decreases plasma (LDL-C).^75–78^ From the structural point of view, 41 has three *para*-substituted phenyl rings, a stereogenic benzylic hydroxyl, and two additional chiral centers at the 2-azetidinone skeleton.^76–84^ Ezetimibe 41 was synthesized starting with isoxazolidine 33 which was subjected to a ring opening of the lactone moiety upon treatment with LiOH with subsequent neutralization to a free carboxylic moiety followed by treatment of the resultant with Ph_3_P and DIAD at 0 °C to give compound 34 in high yield (80%) and high de purity (24 : 9 dr 9 : 1). In addition, the lessening of the reaction temperature to −10 °C resulted in further improvement in the chemical yield (94%) and *de* (24 : 9 dr 97 : 3). Next, the pure 34 (purified by chromatography) was subjected to N–O bond cleavage in 34 using TMSCl and potassium iodide in wet acetonitrile to give minolactone 35 in 88% yield that was enough pure for being used for the next step. Upon treatment of 35 with Burgess reagent 37 in toluene at 90 °C the desired unsaturated lactone 38 was obtained in satisfactory yield. The double bound of the latter was hydrogenated over PtO_2_, to proceed entirely anti to the aryl substituent of the lactone to produce compound 39 (83%, 98% ee) bearing three chiral centers with identical absolute configurations to those present in ezetimibe 41. The latter was reacted with *t*-BuMgBr in dry ether at 0 °C to give lactone 40 which using H_2_, Pd/C in AcOEt in MeOH to afford the desired target ezetimibe 41 in respectable yield (Scheme 5).^85^

### 2.2. Five-membered heterocycles

**Scheme 6:**
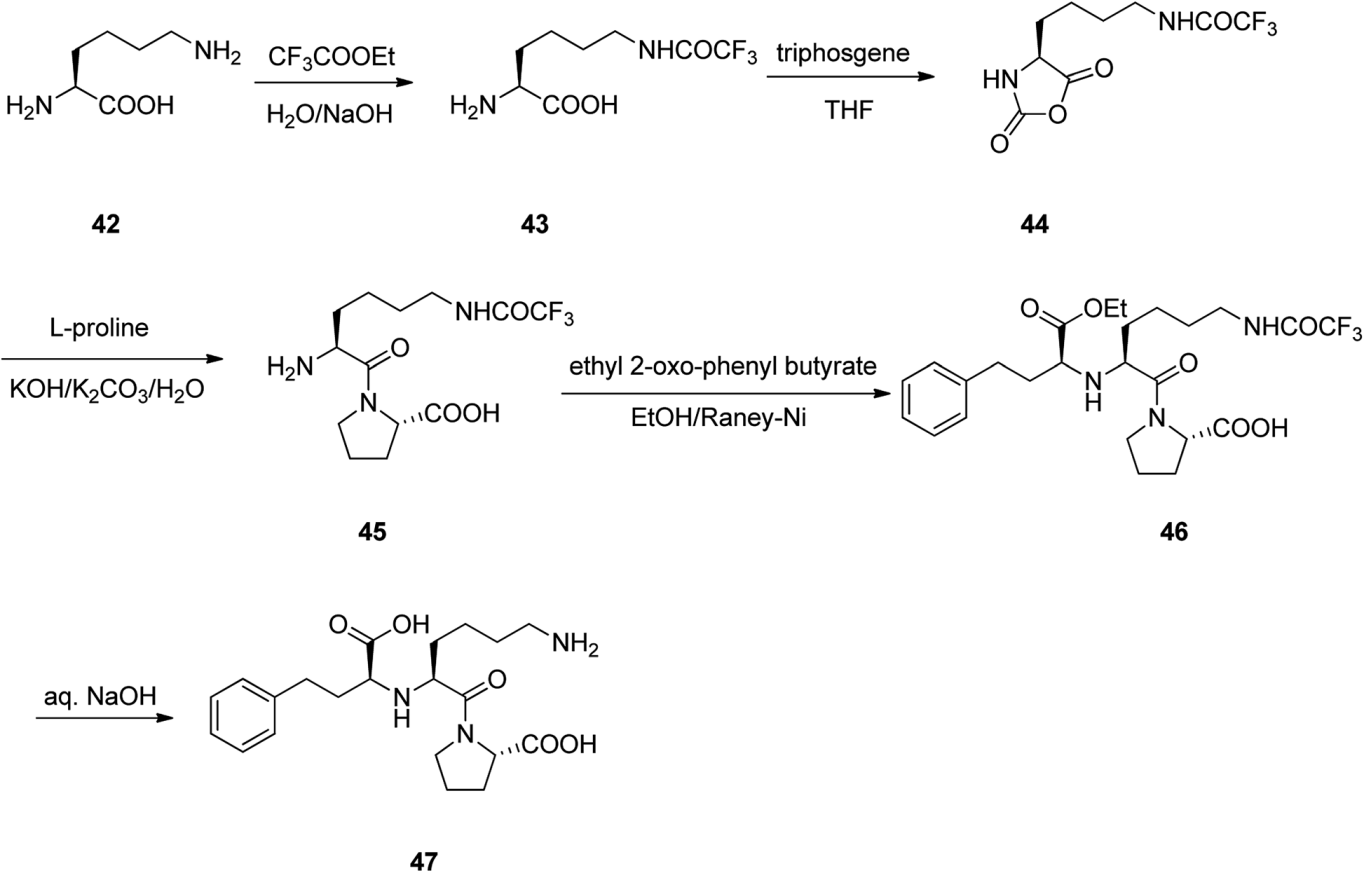
A pathway for the synthesis of lisinopril 47.

Lisinopril 47, is a drug of the angiotensin-converting enzyme (ACE) inhibitor family which is used, primarily in the treatment of hypertension, heart failure, and frequently utilized after heart attack.^86^ Lisinopril 47, chemically is named as *N*^2^-[(1*S*)-1-carboxy-3-phenylpropyl]-l-lysyl-l-proline, but sold under the brand name of PRINVIL® provided by Merck. Lisinopril 47 was patented in 1978, and approved for medical use in the United States in 1987.^87–89^ The synthetic pathway for lisinopril is depicted in Scheme 6. Its multistep synthesis, started with l-lysine 42 which upon treatment with ethyltrifluoro acetate afforded *N*^6^-trifluoroacetyl-l-lysine 43. The latter was reacted with triphosgene to provide *N*^6^-trifluoroacetyl-*N*^2^-carboxy-l-lysine anhydride 44 that was condensed with l-proline to give *N*^6^-trifluoroacetyl-l-lysyl-l-proline 45. The latter was condensed with ethyl 2-oxo-4-phenyl butyrate with subsequent hydrogenation using the RANEY® as catalyst to obtain *N*^2^-(1-(*S*)-ethoxycarbonyl-3-phenylpropyl)-l-*N*^6^-(trifluoroacetyl)-l-lysyl-l-proline 46. Lastly, upon the hydrolysis of the latter with sodium hydroxide, lisinopril 47 was obtained in pure form.^86^

**Scheme 7:**
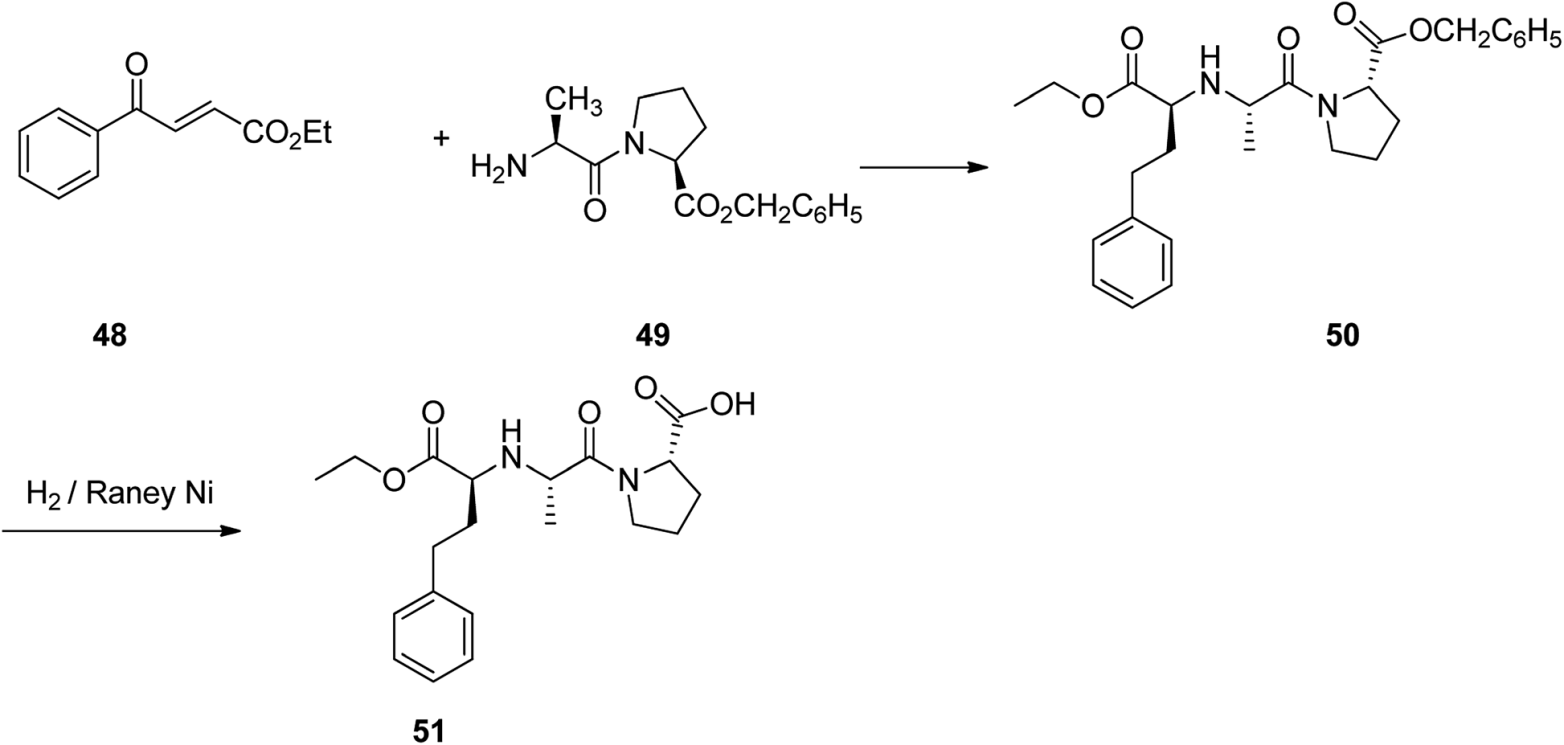
Synthesis of enalapril 51.

Enalapril 51, commercialized under the brand name Vasotec among others, is a drug, employed for the treatment of high blood pressure, kidney disease caused by diabetes and heart failure in which is frequently used with a diuretic, such as furosemide.^90^ Enalapril was patented in 1978, and approved as prescribed drug, coming to market in 1984. Enalapril, (*S*)-1-[*N*-[1-(ethoxycarbonyl)-3-phenylpropyl]-l-alanyl]-l-proline 51, is prepared by treating the benzyl ester of l-alanyl-l-proline 49 with the ethyl ester of 3-benzoylacrylic acid 48 that affords the product 50, in which *via* hydrogenation in the presence of a RANEY® as catalyst eliminates the protective benzyl moiety, affording the desired prescribed drug enalapril 51.^91^ Some other alternative approaches to obtain enalapril have been also advocated (Scheme 7).^92–96^

**Scheme 8:**
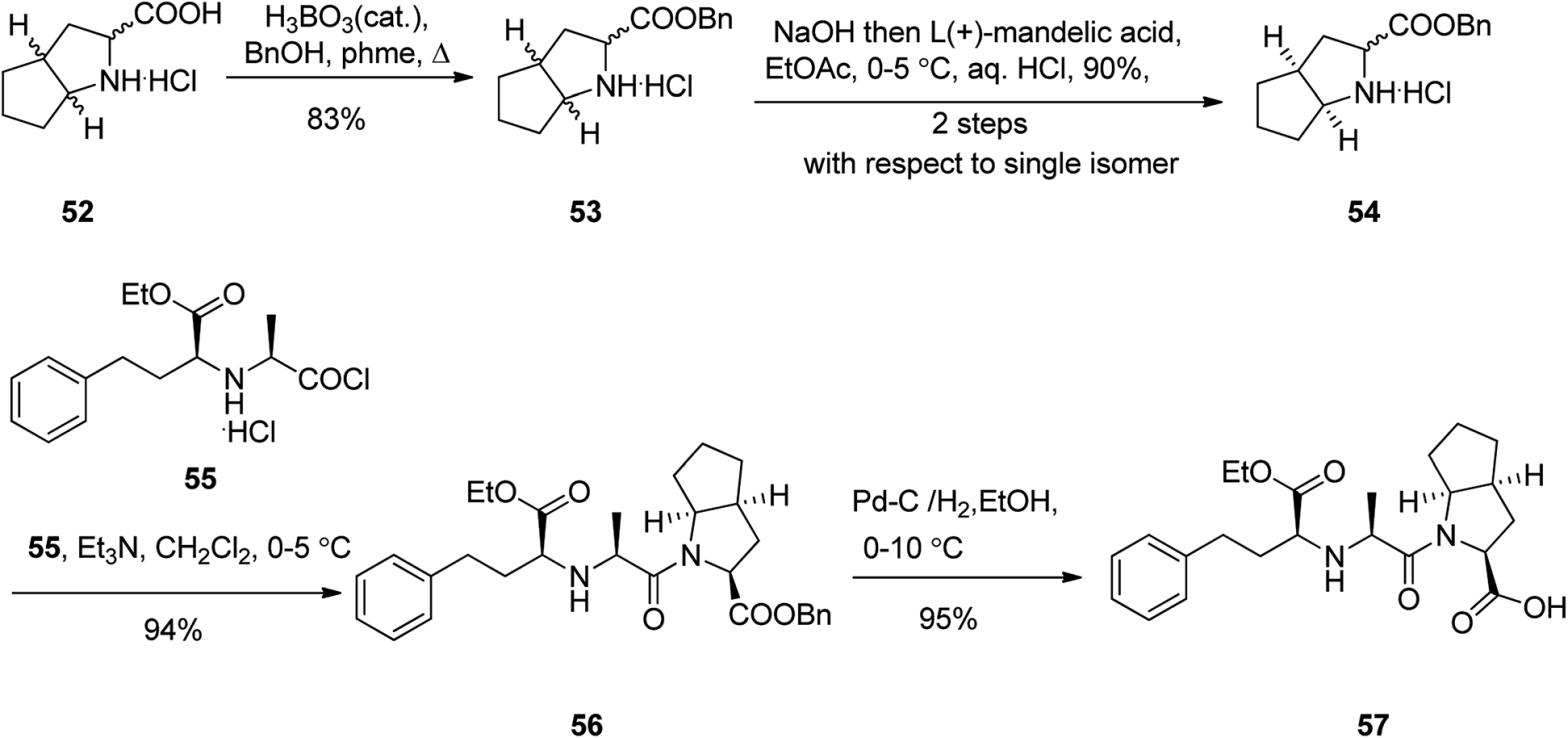
Synthetic route to ramipril 57.

Ramipril 57, is accessible in pharmacies under the brand name, Altace® as capsules.^97^ Ramipril 57 with a chemical name of 2-aza-bicyclo-[3.3.0]-octane-3-carboxylic acid is placed in angiotensin converting enzyme (ACE) inhibitors type drug,^98^ that are utilized as hypertensive, treatment of congestive heart failure. Just a few references can be found in literature describing the synthesis of ramipril 57 in detail.^99^ A highly operative, cost-effective, and more importantly enantioselective synthesis of ramipril was achieved and reported using an environmentally benign process. It started with esterification of racemic 2-aza-bicyclo-[3.3.0]-octane-3-carboxylic acid hydrochloride 52 with benzyl alcohol in refluxing toluene in the presence of boric acid as a catalyst, followed by a fully-bodied resolution using cheap and recyclable l-(+)-mandelic acid as vital steps to give the ester, the (*S*,*S*,*S*)-2-aza-bicyclo-[3.3.0]-octane-3-carboxylic acid benzyl ester 54 in 83%. The latter was then coupled with benzyl *N*-(2*S*-carbethoxy-3-phenyl propyl)-*S*-alanine acid chloride 55 in the presence of Et_3_N in CH_2_Cl_2_ to provide ramipril benzyl ester 56 in 94% yield. Lastly, ramipril benzyl ester 56 was hydrogenated over Pd/C in EtOH to afford, the desired target, optically pure ramipril 57 in 95% chemical yields (Scheme 8).^100^

**Scheme 9:**
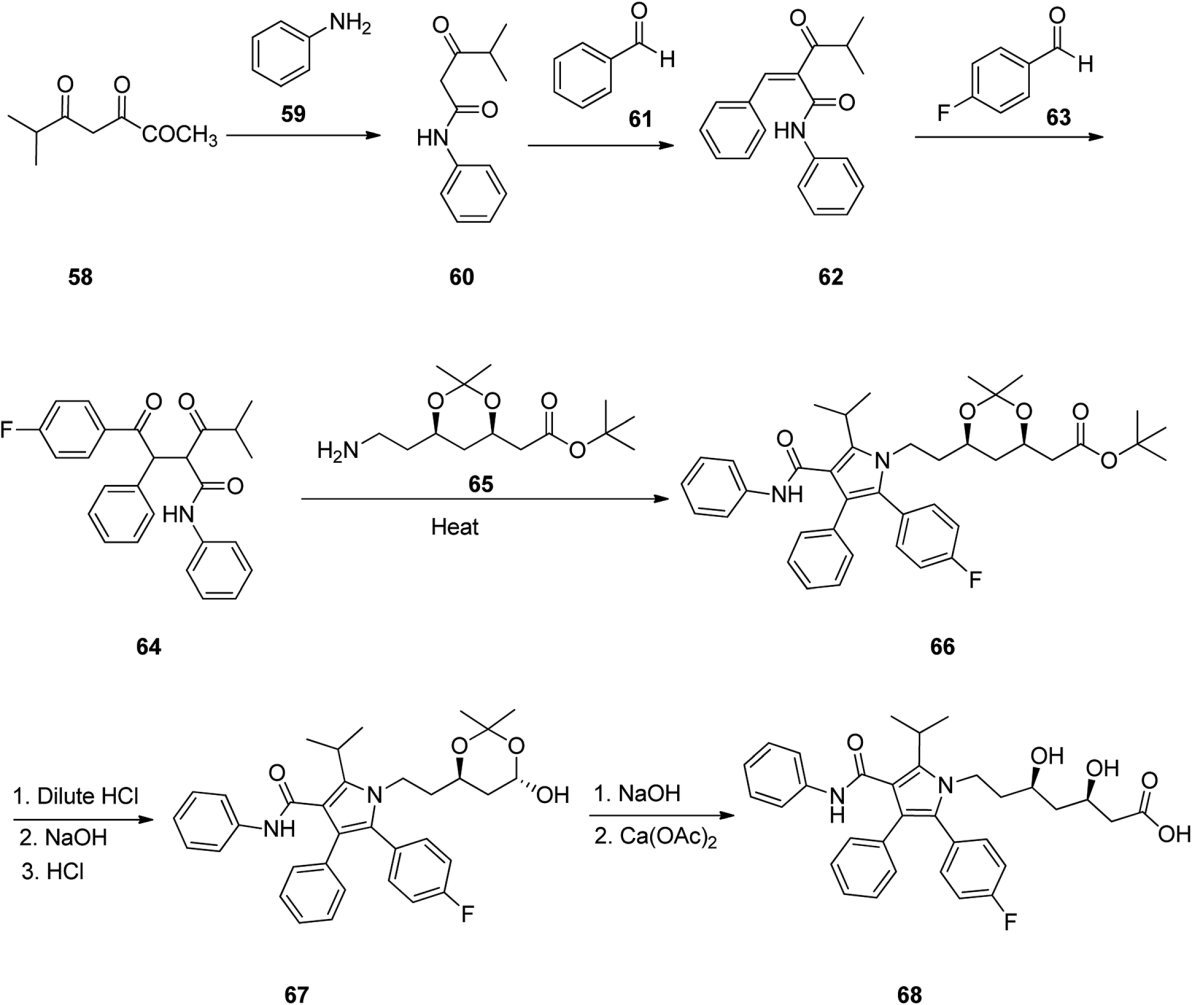
Total synthesis of atorvastatin 68.

Atorvastatin 68 is placed among other suggested prescribed oral statin drugs, is sold under the brand name Lipitor. It is known to inhibit cardiovascular sickness by decreasing levels of low density lipoprotein (LDL) cholesterol level in blood. Atorvastatin was patented in 1986, and gained approval for being prescribed in US in 1996^101^ and currently is accessible as a generic drug.^102^ In 1989, Butler and co-workers, achieved and reported a successful synthetic approach for the atorvastatin 68 for the first time, comprising of six steps.^103^ This multistep approach started with 4-methyl-3-oxopentanoic acid methyl ester 58 which upon heating with aniline and ethylene diamine in toluene as solvent afforded 4-methyl-3-oxo-*N*-phenylpentanamide 60. The latter is reacted with benzaldehyde in hexane in the presence of catalytic amount of β-alanine and glacial acetic acid *via* Knoevenagel condensation provided 4-methyl-3-oxo-*N*-phenyl-2-(phenylmethylene)pentanamide 62. The latter was reacted with 4-fluorobenzaldehyde in the presence of catalytic amount of 3-ethyl-5-(2-hydroxyethyl)-4-methylthiazolium bromide and Et_3_N in ethanol at 80 °C to give diketone 64. The latter was then reacted with (4*R-cis*)-1,1-dimethyl-6-(2-aminoethyl)-2,2-dimethyl-1,3-dioxane-4-acetate 65 in the presence of pivalic acid as catalyst in toluene-heptane as co-solvent system, to give poly-substituted Paal–Knorr pyrrole 66. Upon deprotection of 66 using dilute HCl and subsequent treatment of deprotected diol intermediate, with sodium hydroxide for the removal of *tert*-butyl ester group followed by acidification using HCl under mild heating lactone 67 was obtained. Lastly, treatment of the latter with sodium hydroxide, initially resulted in the cleavage of the lactone ring, followed by additional treatment of the corresponding sodium salt intermediate with 0.5 equivalent of calcium acetate to provide the desired target, atorvastatin calcium salt 68 (Scheme 9).^104^

**Scheme 10:**
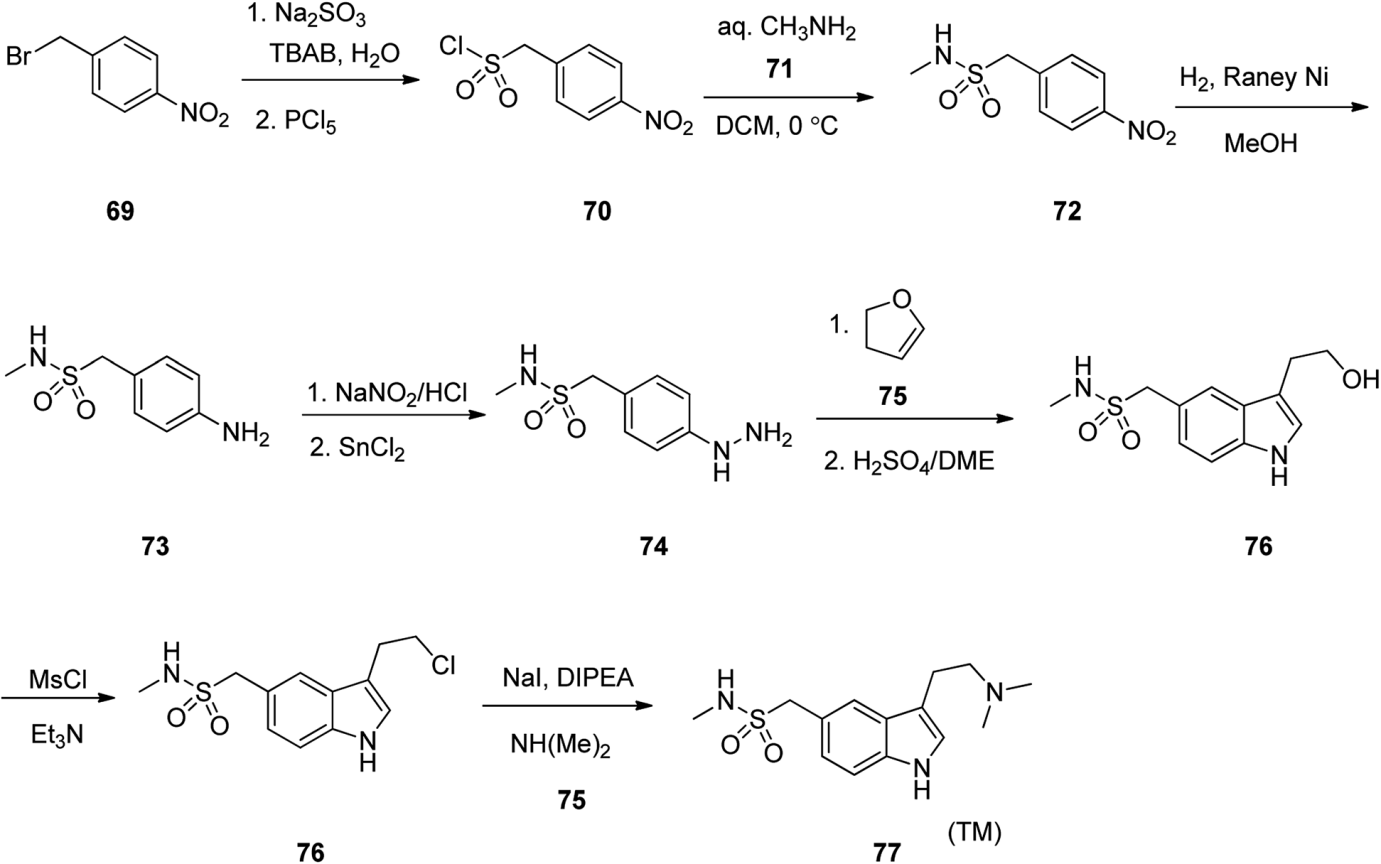
Synthesis of sumatriptan 77.

Sumatriptan, sold under the brand name Imitrex among others. Sumatriptan was patented in 1982 and approved for medical use in 1991.^105^ Sumatriptan (TM) 79 is a highly efficient and selective serotonin (5-HT_1d_) receptor agonist that is used on the treatment of migraine attacks.^106^ Literature survey revealed a plethora of information regarding the synthesis of sumatriptan, mostly patented^107–112^ In an attempt, starting with 1-(bromomethyl)-4-nitrobenzene 69, it was treated with Na_2_SO_3_ in TBAB with subsequent reaction of the resultant with PCl_5_ (4-nitrophenyl)methanesulphonyl chloride was obtained 70. The latter was then reacted with methyl amine 71 in dichloromethane to afford the corresponding sulfonamide 72. The latter upon catalytic hydrogenation over RANEY® transformed –NO_2_ group to –NH_2_ group gave compound 73. The latter was then treated with NaNO_2_/HCl, followed by reduction with SnCl_2_ gave the corresponding aryl hydrazine 74 as a key intermediate. The aryl hydrazine 74 was next, reacted with dihydrofuran 75 with subsequent treatment with anhy·H_2_SO_4_/DEM gave the corresponding indolyl alcohol 76. The latter was then treated with MsCl/Et_3_N to afford the corresponding chloride 77 which was subjected to amination with dimethyl amine 78 to give sumatriptan 79 (Scheme 10).^113^

**Scheme 11:**
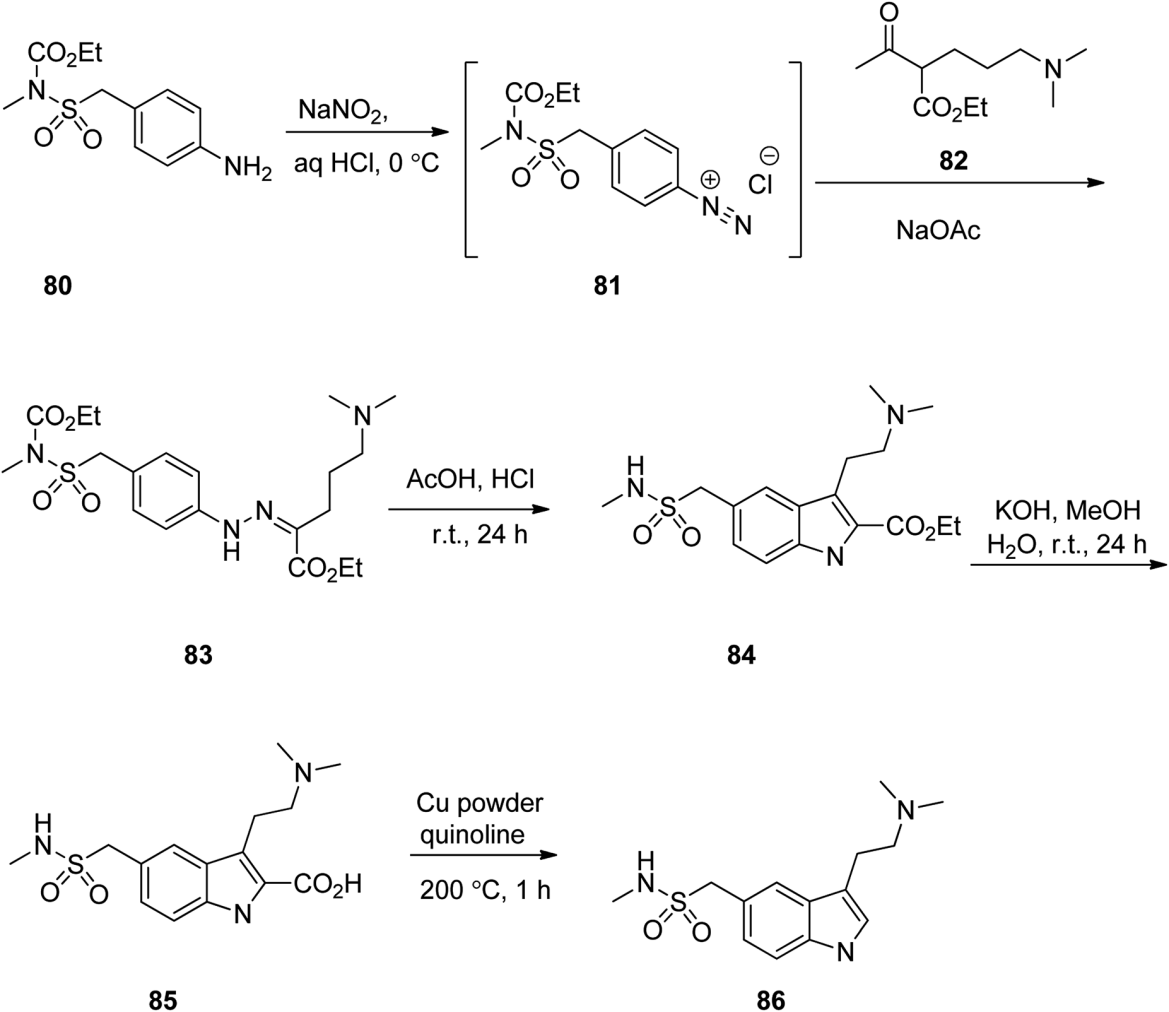
Preparation of sumatriptan 86*via* combination of Japp–Klingemann and Fischer-indole.

Sumatriptan (TM) could also be synthesized, starting with compound 80 which first was converted to 83*via* reaction with NaNO_2_/HCl at 0 °C to give the corresponding diazonium salt 81, followed by direct reaction of the resultant with a β-ketoester under Japp–Klingemann reaction conditions in one-pot manner. Hydrazine 83 was subjected to intramolecular Fischer indole synthesis upon treatment with AcOH/HCl at room temperature to provide the expected corresponding indole derivative 84. The ester 84 upon treatment with KOH/MeOH at ambient temperature was hydrolyzed to the corresponding acid 85. The latter was then decarboxylated in the presence of Cu powder in quinolone at 200 °C to provide the desired sumatriptan in 80% yield (Scheme 11).^114^

**Scheme 12:**
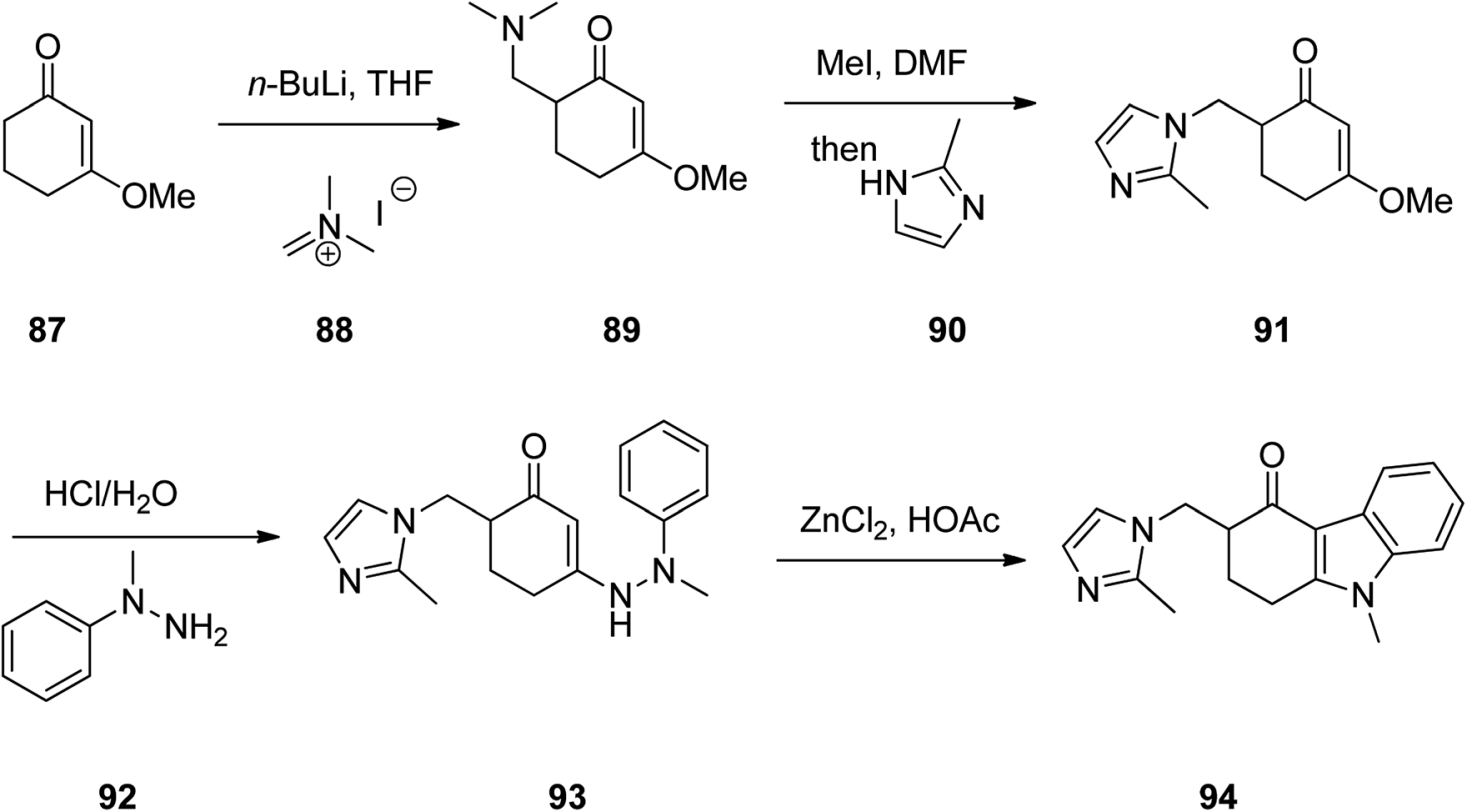
Fischer indole synthesis of ondansetron 94.

Ondansetron, 1,2,3,9-tetrahydro-9-methyl-3-(2-methyl-1*H*-imidazol-1-ylmethyl)-4*H*-carbazol-4-one hydrochloride dihydrate 94, commercialized under the brand name Zofran. Ondansetron was patented in 1984 and approved for medical use in 1990.^115^ It is a medication used to prevent nausea and vomiting caused by cancer chemotherapy, radiation therapy, or surgery.^116,117^ The pharmacologic and therapeutic applications of ondansetron have extensively been reviwed.^118–124^ It has been synthesized in five steps starting from 3-methoxycyclohex-2-en-1-one 87 which initially treated with CH_2_N^+^(CH_3_)_2_I^−^88 in the presence of *n*-BuLi in THF to provide 6-((dimethylamino)methyl)-3-methoxycyclohex-2-enone 89 in 54% yield. Then, the latter was reacted with MeI in DMF and then reacted with 2-methyimidazole 90 to give 3-methoxy-6-((2-methyl-1*H*-imidazol-1-yl)methyl)cyclohex-2-enone 91 in one-pot fashion in 83% yield. The latter was first treated with HCl/H_2_O and then reacted with phenyhydrazine 92 to provide 6-((2-methyl-1*H*-imidazol-1-yl)methyl)-3-(2-methyl-2-phenylhydrazinyl)cyclohex-2-enone 93. The latter was finally subjected to Fischer indole synthesis at the presence of ZnCl_2_/HCl in which phenyl-methyl hydrazine and a cyclic 1,3-dione derivative are intramolecularly were cyclized to provide the fully substituted tricyclic core of the desired ondansetron 94 (Scheme 12).^125^

Tadalafil 99 under the brand name of Cialis acts is also used in erectile dysfunction (ED). The core structure in talalafil is a tetrahydro-β-carboline. Its synthesis is relatively straightforward and is relied on the work of Anand^126^ and co-workers *via* the straightforward multistep synthesis using four key framework, namely, d-tryptophan methyl ester 95, market accessible piperonal 96, chloroacetyl chloride, and methylamine.^127^

**Scheme 13:**
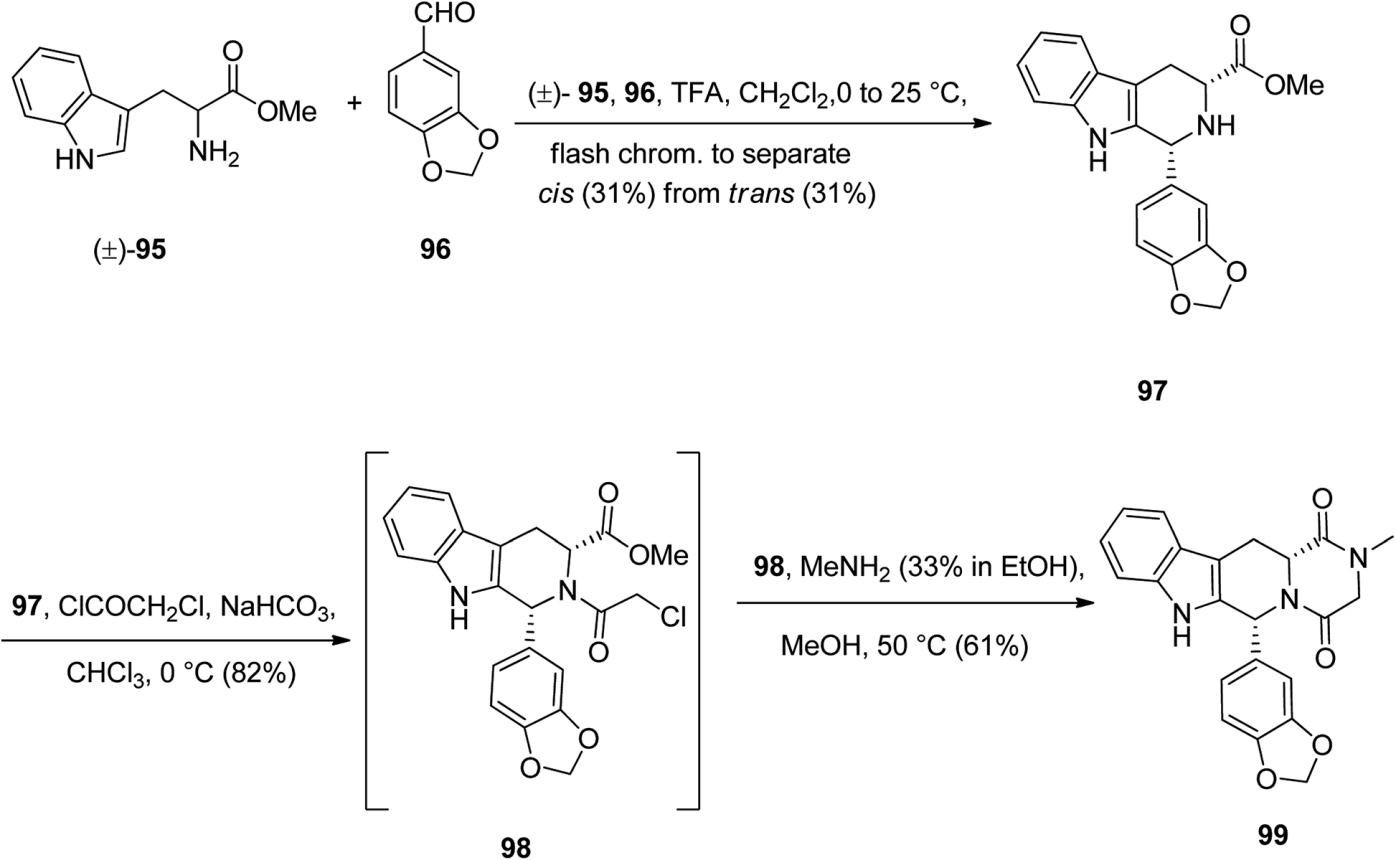
Synthetic pathway to tadalafil 99.

The pathway to provide tadalafil for clinical use is depicted in Scheme 13.^128,129^ Initially, commercially available tryptophan methyl ester 95 as racemic mixture was reacted with 96 under intermolecular Pictet–Spengler type reaction conditions at room temperature to give 97 as a mixture of the *trans*-isomer (31%) and the desired lower melting *cis*-isomer (31%), which was separated by flash chromatography. Noticeably, the later patents claim that the yield could be improved to 42% of the *cis*-isomer, with 28% of the *trans*-isomer if the Pictet–Spengler type reaction is performed at 4 °C instead of room temperature.^130^ The required *cis*-isomer 97 upon acetylation with chloroacetyl chloride afforded 98 which treated with methylamine, to give tadalafil 99 as clinically, pure form.^131^

**Scheme 14:**
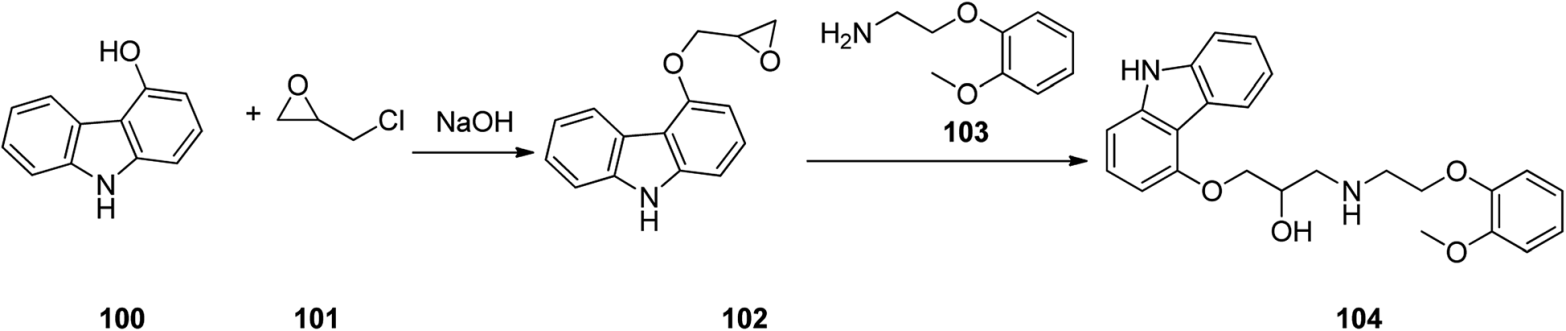
Synthesis of carvedilol 104.

Carvedilol 104, sold in market under brand name of Coreg is actually a third-generation β_1_ and β_2_ blocker that also possesses α_1_-adrenergic-blocking potencies. Carvedilol 104 is actually produced *via* a two-step synthesis through reaction of 4-hydroxycarbazole 100 with epichlorohydrin in the presence of NaOH to provide 4-(2,3-epoxypropoxy)carbazole 101 which after isolation, treated with 2-(2-methoxyphe-noxy)ethanamine 103 to afford the desired target, carvedilol 104 (ref. 132) (Scheme 14). Alternative approaches for the formation of carvedilol have also been reported.^133–138^

**Scheme 15:**
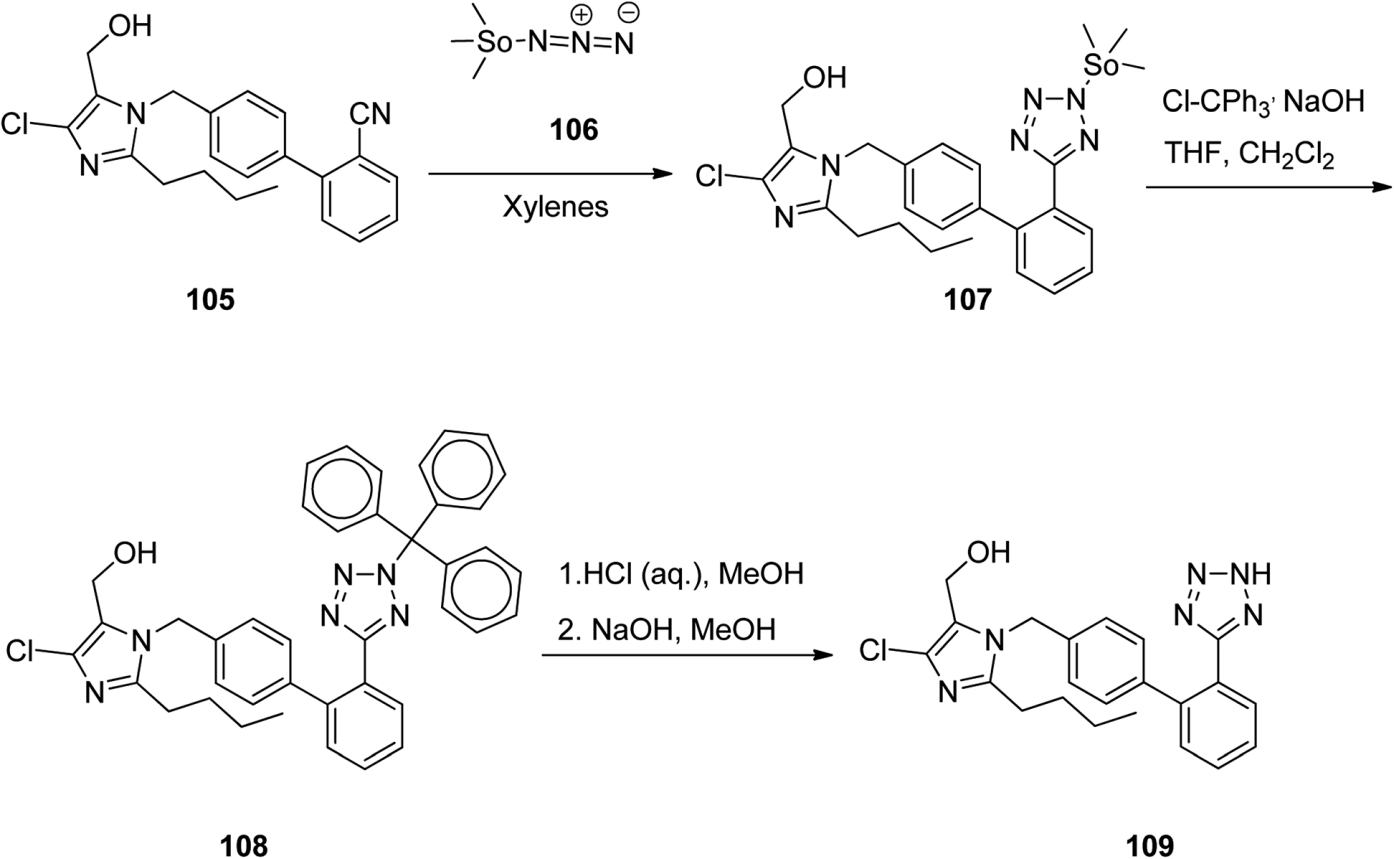
Preparation of losartan 109.

Losartan 109, marketed under the trade name Cozaar among other names, patented in 1986, and approved by FDA for being prescribed in 1995.^139^ It is a drug, chiefly prescribed for the treatment of high blood pressure. As illustrated in Scheme 15,^140^ the tetrazole ring of losartan 109 is constructed by treating of 1-[(2′-cyanobiphenyl-4-yl)methyl]-2-butyl-4-chloro-5-hydroxymethylimidazole 105 with trimethyltin azide 106. The aforementioned reaction affords a trimethylstannyl-substituted tetrazole compound 107, straightly. The trimethylstannyl motif is eliminated from the intermediate 107 by treating with trityl chloride. This reaction leads to the introduction of the trityl group to the tetrazole ring. In the final step, the trityl group is lost under acidic conditions to afford losartan 109. In this way, losartan 109 was obtained in 88.5% yield and 98.8% ee.^141^

**Scheme 16:**
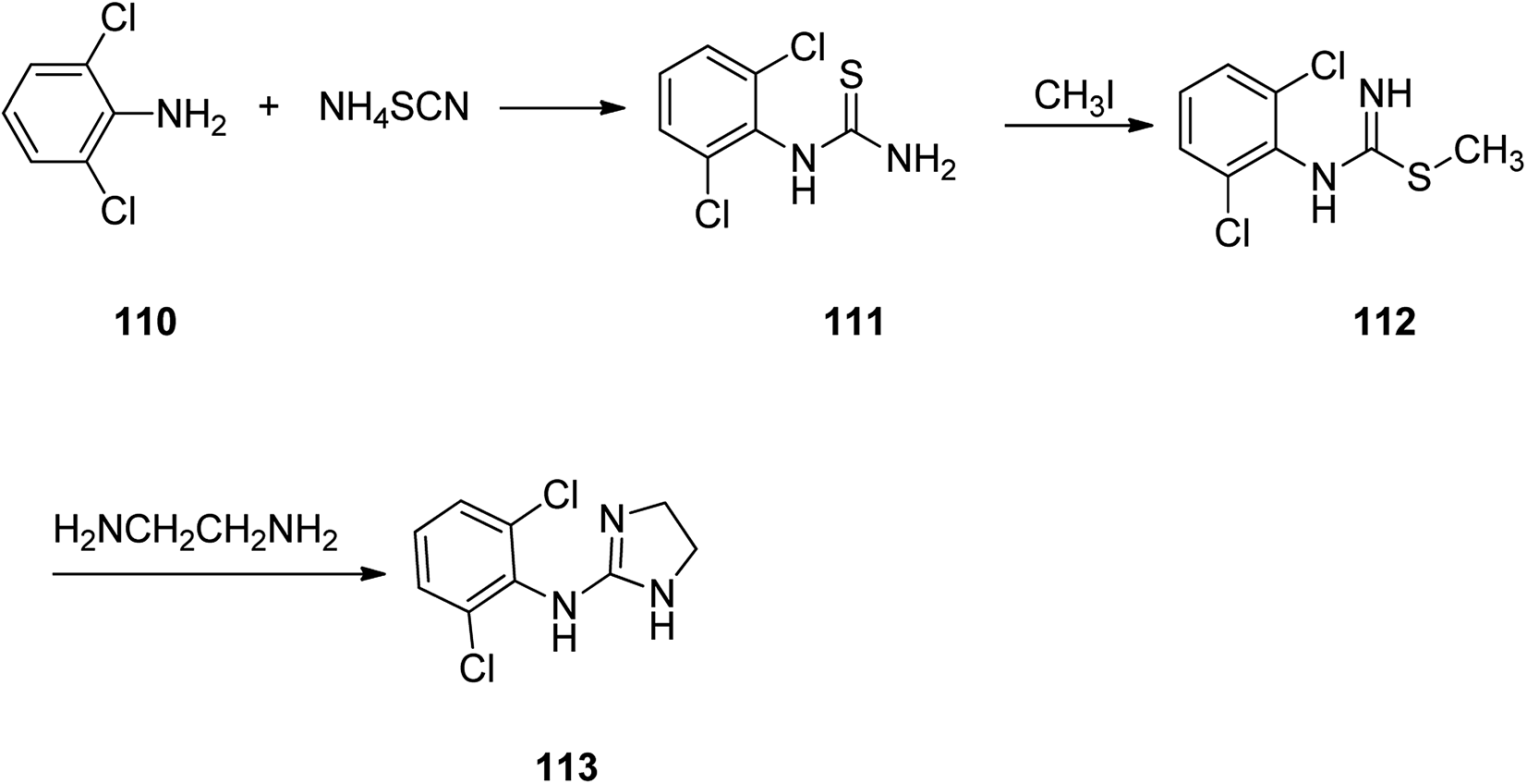
Synthesis of clonidine 113.

Clonidine, 2-(2,6-dichlorophenylamino)imidazoline 113, is also on market under the brand name Catapres. It is commonly prescribed for the treatment of high blood pressure, attention deficit hyperactivity disorder. Clonidine was patented in 1961 approved and came to market in 1966.^142–144^ It is an *anti*-hypertensive medicine with a clear-cut central site of act. The pharmacology of clonidine 113 has extensively been covered by Kobinger^145^ and Walland.^146^ Worthy to mention that introduction of the two chlorine atoms in *ortho* positions onto the 2-(arylimino)imidazolidine was found being vital for the biological activity of clonidine (Scheme 16).

**Scheme 17:**
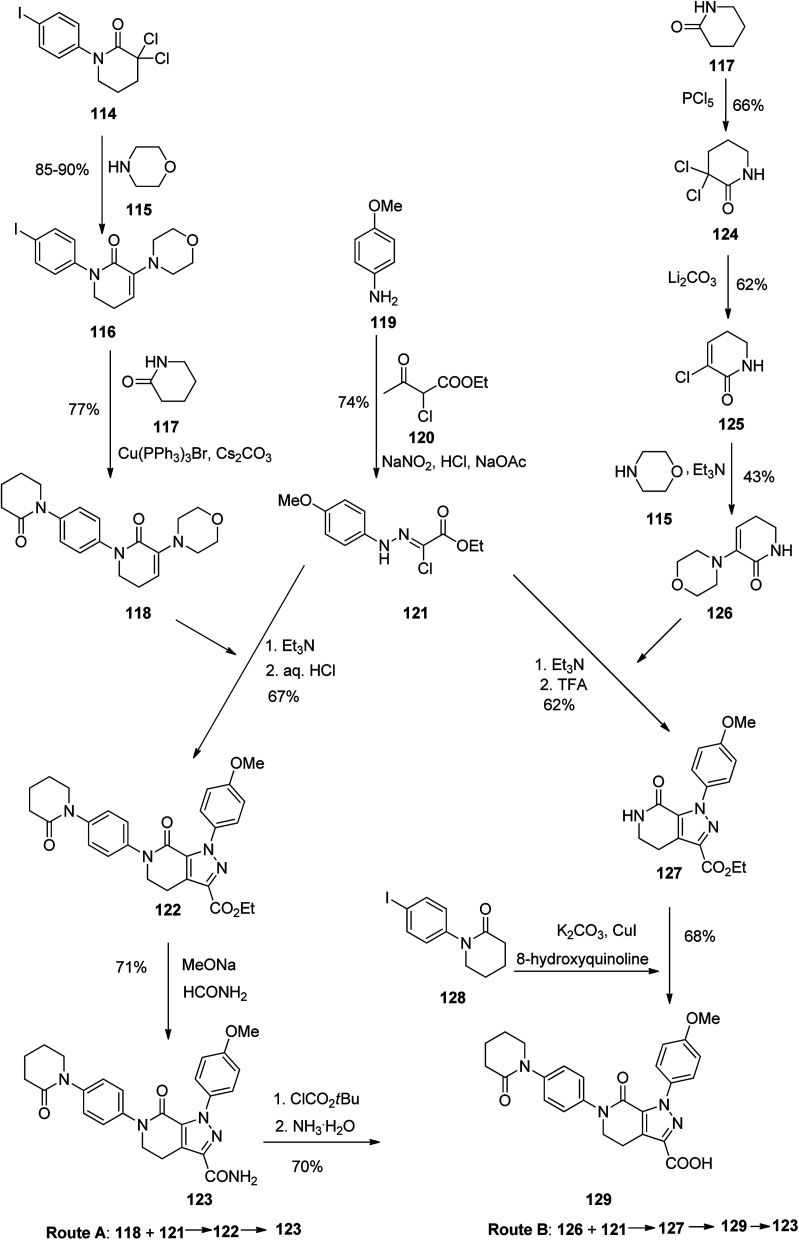
Tow routes for the formation of apixaban 123.

Apixaban, sold under the brand name eliquis was pataned in 2012 but approved in December 2019. Apixaban 123 is a powerful, selective, and orally bioavailable inhibitor of blood coagulation factor Xa (fXa).^147,148^ In addition, it has also exhibited promising in treatment of severe coronary syndrome (ACS),^149^ cerebrova scular ischemia, and cancer.^150^ Various pathways for the formation of apixaban 123 have been demonstrated, chiefly based on the application of expensive organic iodide.^151–153^ In the early age of drug discovery, 2003, Zhou and co-workers^151^ achieved and reported two approaches for the synthesis of 123 (Scheme 17, pathways A and B). In pathway A, initially, hydrazine 121, as a vital intermediate, was provided in two steps through the diazotization of 4-methoxyaniline 119 with subsequent Japp–Klingemann reaction with ethyl 2-chloroacetoacetate. Next, hydrazine 121 was exposed to an addition–elimination sequence with *N*-phenylvalerolactam 118 to afford pyrazolecarboxylate 122. Lastly, aminolysis of the latter with 10 equiv. of formamide and sodium methoxide (MeONa) resulted in the desired target 123. Particularly, *N*-phenylvalerolactam 118 was produced *via* an Ullmann reaction that treated with iodide 116 in 77% yield but organic cuprous compound Cu(PPh_3_)_3_Br as catalyst was needed. In pathway B, pyrazolecarboxylic acid 129 upon treatment with isobutyl chloroformate gave a mixed anhydride, which with subsequent aminolysis using ammonia, affording the desired target 123. In an alternative method, enamine 126 was reacted with key intermediate 121*via* a sequential addition–elimination to afford pyrazololactam 127 which was subsequently undergone an Ullmann coupling reaction with organic iodide 128 using CuI to form 129 in 68% yield (Scheme 17).^154^

**Scheme 18:**
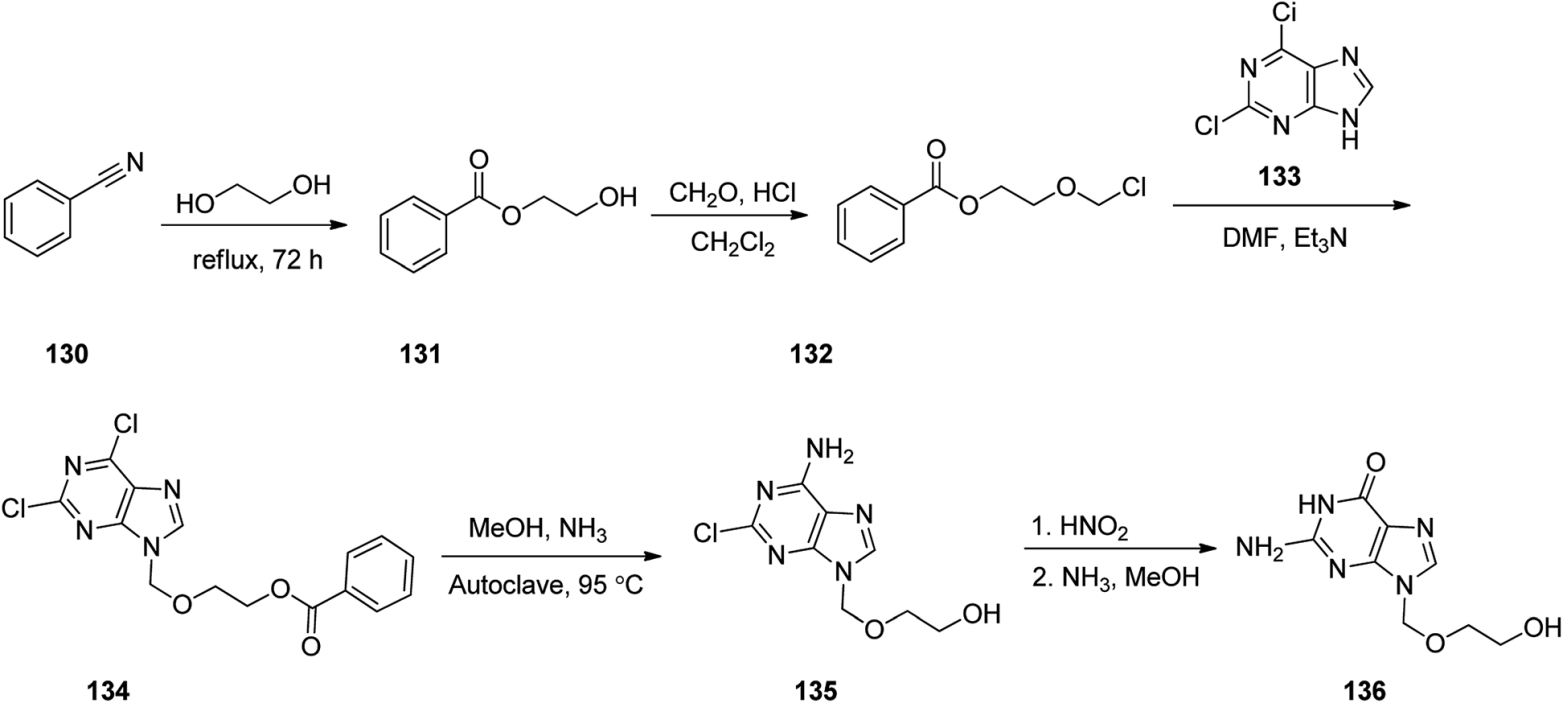
Synthesis of acyclovir 136.

Aciclovir 136, was patented in 1974, and approved by FDA for medical use in 1981.^155^ It is an antiviral medicine,^156^ functioning by decreasing the production of the virus's DNA. It is prescribed for the treatment of herpes simplex virus infections, chickenpox, and shingles.^157^ Acyclovir 136 with chemical name of {9-[(2-hydroxyethoxy)methyl]guanine} (ACV), sold in market under the brand name of Zovirax.^158^ It functions selectively on herpes cells by specific inhibitory effects on repetition of herpes virus. In fact, acyclovir 136, the first effective antiviral agent and as mentioned above is a nucleoside analog.^159^ The synthetic pathway for acyclovir 136 was first disclosed in 1978.^158^ and then described in detail later.^160^ The synthetic route began with benzonitrile 130 which reacted with refluxing ethylene glycol to provide ethylene glycol monobenzoate 131. A cold mixture of the latter and paraformaldehyde in dry CH_2_Cl_2_ was saturated with hydrochloric acid, providing 1-benzoyloxy-2-chloromethoxyethane 132. Addition of the latter to a solution containing, 2,6-chloropurine 133 and Et_3_N in DMF, gave 2,6-chloro-9-(2-benzoyloxye-thoxymethyl)purine 134 in pure form. A solution of 134 in ammonia/methanol solution was heated in autoclave at 95 °C to give 2-chloro-9-(hydroxyethoxymethyl)adenine 135. As a matter of fact, the latter is the result of the known variances in the chemical reactivity in the 2- and 6-positions in the pyrimidine ring, leading to selective substitution of the 6-chloro group along with simultaneous deprotection of the side chain. The latter upon treatment with nitrous acid, followed by reaction of deaminated intermediate with methanolic ammonia to substitute the 2-chloro group, provided the desired target, acyclovir 136 a moderate yield (Scheme 18).^161^

**Scheme 19:**
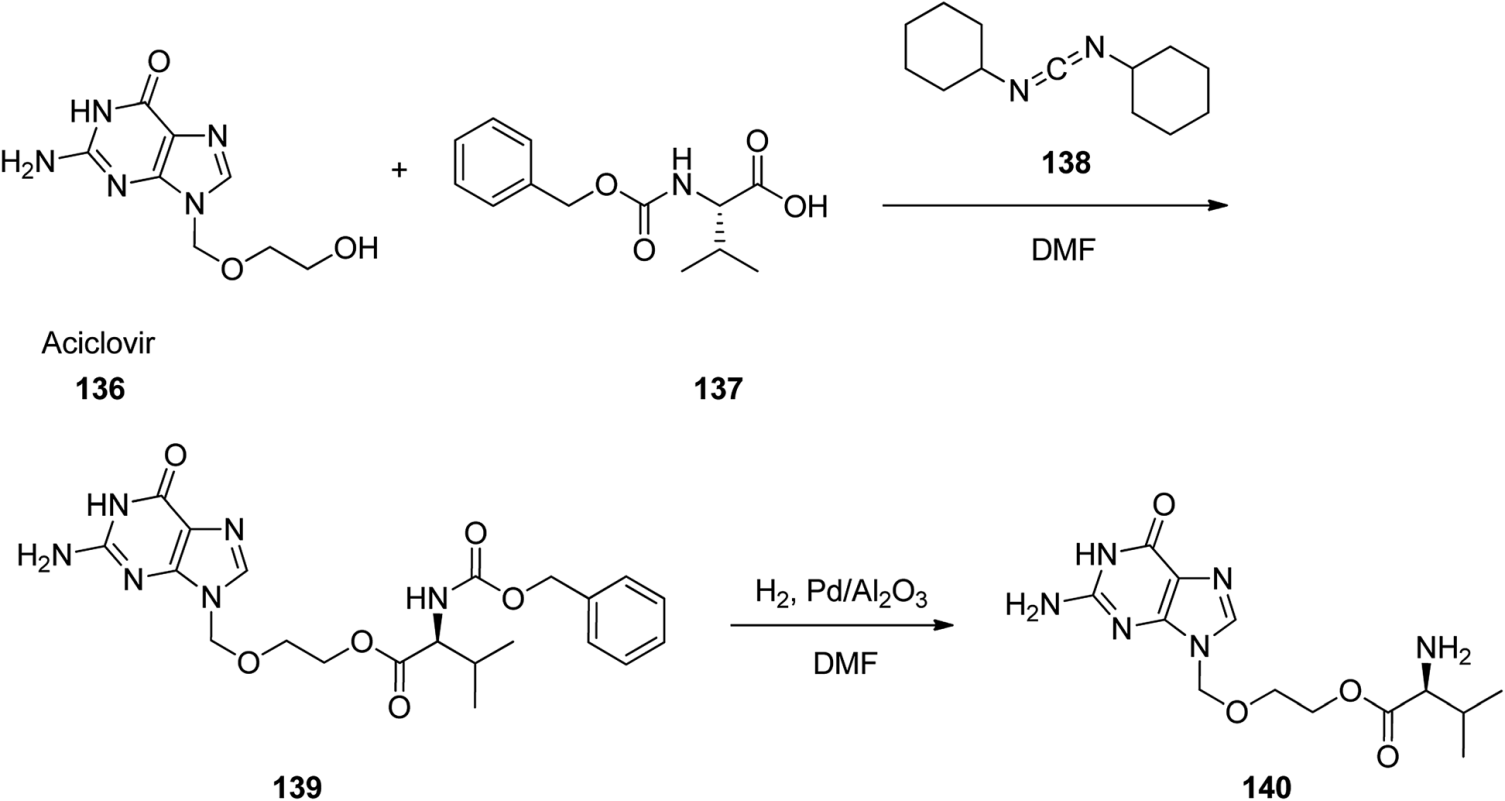
Synthesis of valacyclovir 140.

Valacyclovir, l-valine, 2-[(2-amino-1,6-dihydro-6-oxo-9*H*-purin-9-yl)methoxy]ethyl ester, monohydrochloride 140 which is also available in market under the brand name vitrax. Valaciclovir was patented in 1987, approved by FDA, came into medical use in 1995.^162^ Valacyclovir 140 is a prodrug derived by esterifying acyclovir with l-valine. It is rapidly absorbed and well tolerated.^163–168^ A facile and multipurpose synthesis of valacyclovir 140,^169,170^ acyclovir 136 was condensed with *N*-carbobenzyloxy-l-valine 137 in the presence of dicyclohexylcarbodiimide in dimethylformamide to *N*-carbobenzyloxy-protected valacyclovir 139 that was subjected to palladium catalyzed deprotection (palladium/aluminium oxide in dimethylformamide) to provide valacyclovir 140. Later, an effective and scalable process was established, as illustrated in Scheme 19.^171^ The same method was performed with another masking group on an amino acid scaffold. Thus, valacyclovir was synthesized *via* reaction of *N*-(Boc)-l-valine with acyclovir employing 1-(3-dimethyl-aminopropyl)-3-ethylcarbodiimide hydrochloride (EDC) as coupling agent and hydrochloric acid in the deprotection step.^172^

**Scheme 20:**
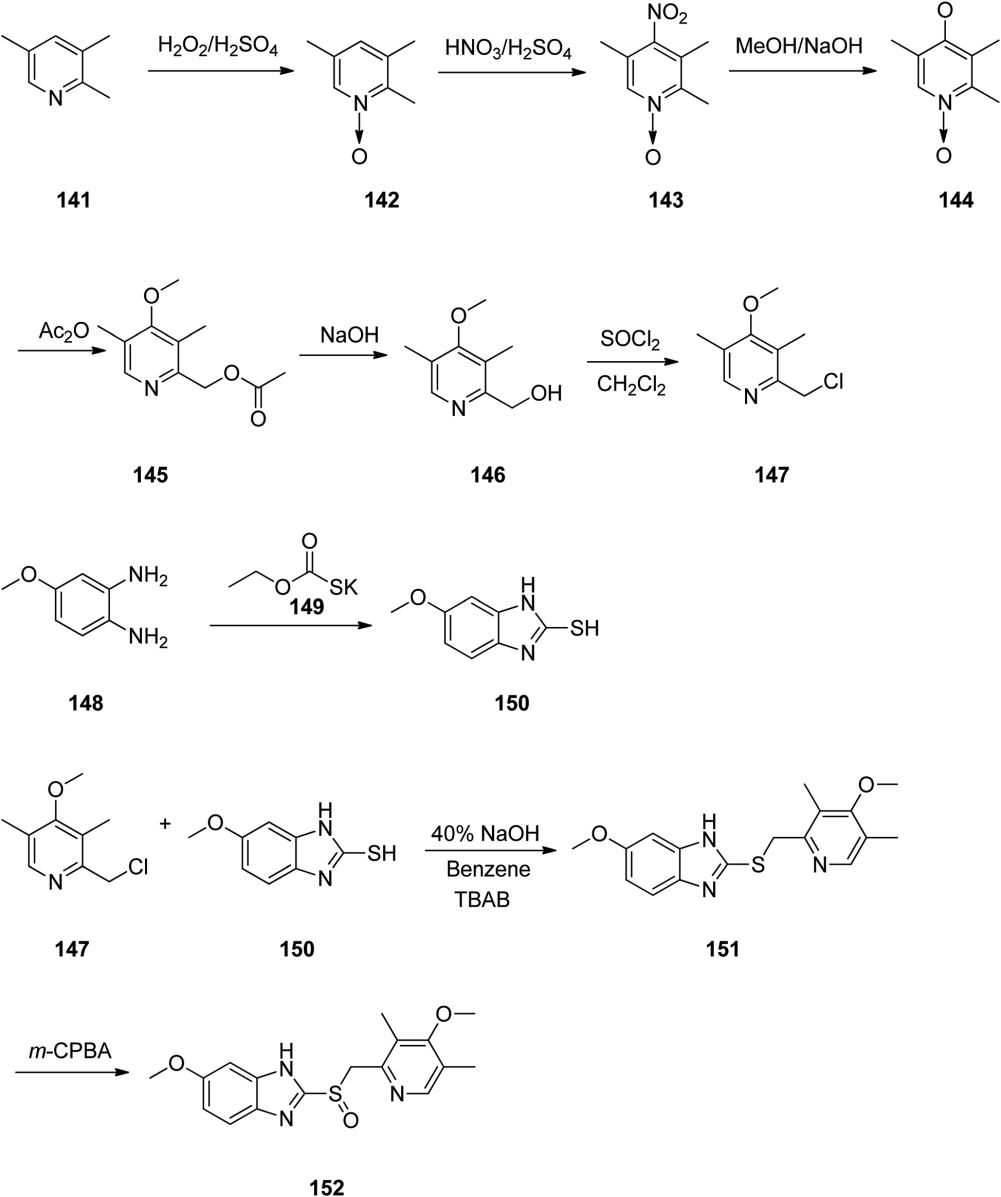
Synthesis of omeprazole 152.

Omeprazole as a racemic mixture was the first recognized proton pump inhibitor (1979) having been marketed under the brand name Prilosec in 1988. As a proton pump inhibitor, omeprazole, like the others (such as lansoprazole, pantoprazol and rabeprazole) share the core structure of pyridinylmethylsulfinyl benzimidazoles.^173^ Different approaches have been reported for the synthesis of omeprazole 152.^174–182^ However, these approaches of synthesizing omeprazole are very close to each other and mainly relied on the first patent where its synthesis was revealed.^183^ The multi-step synthesis of omeparazole was started with 2,3,5-collidine 141 and was oxidized by H_2_O_2_ in HAOc to provide the *N*-oxide 142. The latter was nitrated in a mixture of nitric acid and sulfuric acids to provide the corresponding 4-nitro derivative 143. Then, the nitro group in 143 was substituted by OMe group in methanol/sodium hydroxide to give 144.^184^ The latter was heated in acetic anhydride that concurrently reduced the ring followed by acetylation (Boekelheide rearrangement) to provide the hydroxymethyl-pyridine acetyl derivative 145. In the following, the corresponding alcohol 146 was generated upon treatment with sodium hydroxide, with subsequent conversion to chloride-2-chloromethyl-4-methoxy-2,3,5-trimethylpyridine 147 employing SOCl_2_. On the other hand, condensation of 4-methoxy-*o*-phenylendiamine 148 with potassium ethylxantogenate 149 in the conventional fashion provided 2-mercapto-5-methoxy benzimidazole 150. Reaction of the latter with 2-chloromethylpyridine derivative 147 in the presence of sodium hydroxide in H_2_O/EtOH under reflux, or being performed under phase transfer catalysis conditions (benzene 40% sodium hydroxide, tetrabutyl ammonium bromide) provided thioether-5-methoxy-2-[((4-methoxy-3,5-dimethyl-2-pyridinyl)methyl)thio]-1*H*-benzimidzole 151 (pyrmetazole) that upon oxidation by 3-chloroperbenzoic acid in CH_2_Cl_2_ or H_2_O_2_ gave the corresponding sulfoxide, omeprazole 152 (Scheme 20). Several improved routes for the formation of omeprazole have been reported.^185–193^

**Scheme 21:**
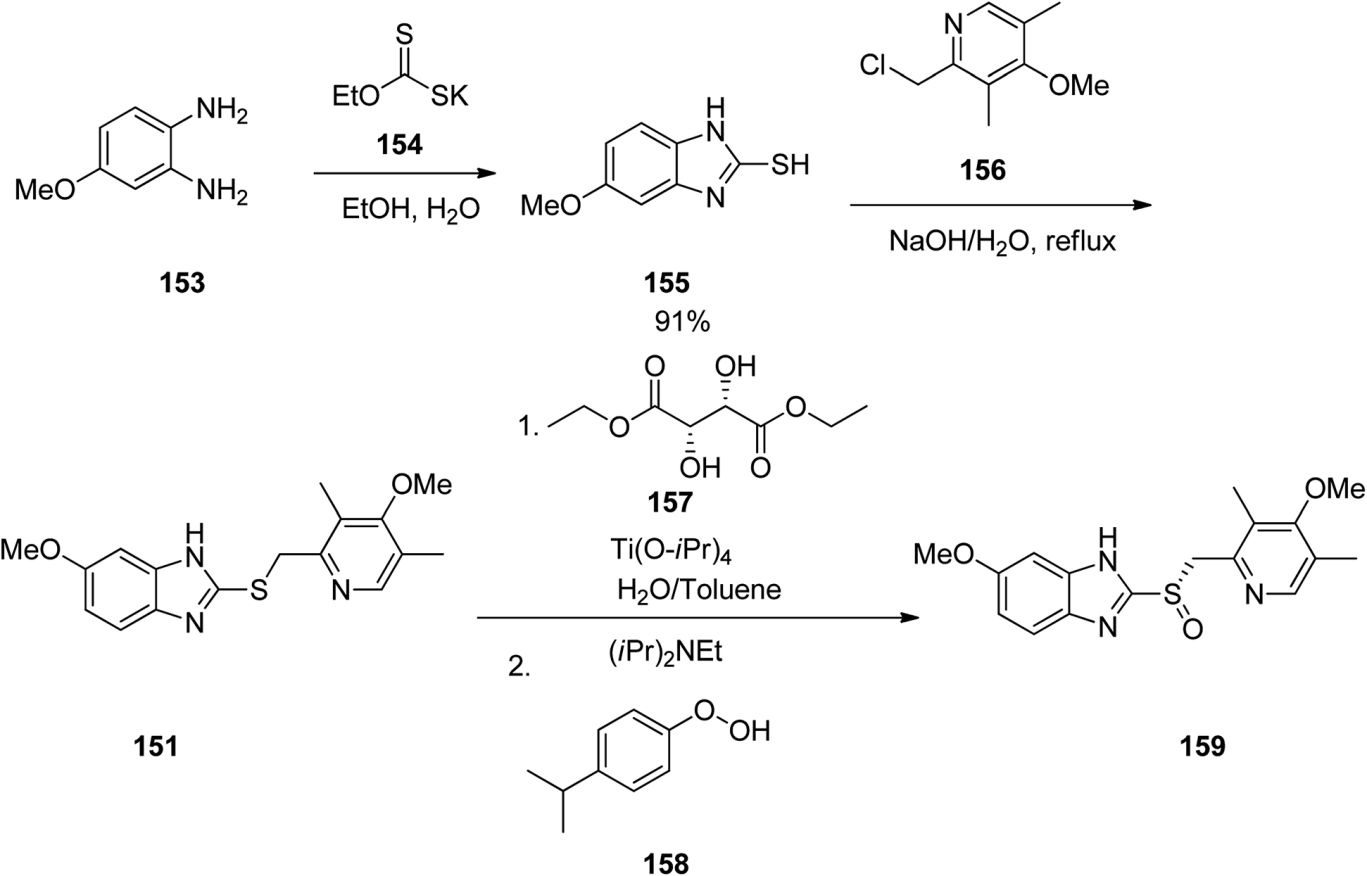
Asymmetric synthesis of esomeprazole 159.

Esomeprazole 159 is existed in the market under the brand names nexium among others, is a medicine which decreases the amount of stomach acid. It was patented in 1993, approved for medical use by FAD in 2000 and is available as a generic pharmaceutical.^194,195^ Structurally, esomeprazole is the (*S*)-(−)-isomer of omeprazole. It functions through blocking H^+^/K^+^-ATPase in the parietal cells of the stomach.^196^ Various methods have been reported for the synthesis of esomeprazole.^197–200^ The asymmetric synthesis of esomeprazole was achieved. The conjoint piece in their synthetic approach is the double condensation of a 1,2-diaminobenzene 153 with potassium ethylxanthate 154.^201^ An archetypal synthesis of the methoxybenzimidazole moiety existed in esomeprazole (omeprazole) is depicted in Scheme 21. For the synthesis of esomeprazole the succeeding steps comprise an *S*-alkylation as well as an asymmetric oxidation of the recently generated thioether.^202,203^ Practically, pyrmeprazole 151 was suspended in toluene and this solution was added to a water solution of (*S*,*S*)-diethyl tartrate and Ti(iso-proxide)_4_. To this solution, *N*,*N*-diisopropylethylamine and cumene hydroperoxide were added. In this way esomeprazole was obtained in 92%, chemical yield and the enantiomeric excess (ee) of crude sulphoxide was about 94%. The obtained esomeprazole was converted to its sodium salt as a solid with an enantiomeric excess of 99.5%, using conc. NaOH solution and CH_3_CN.

**Scheme 22:**
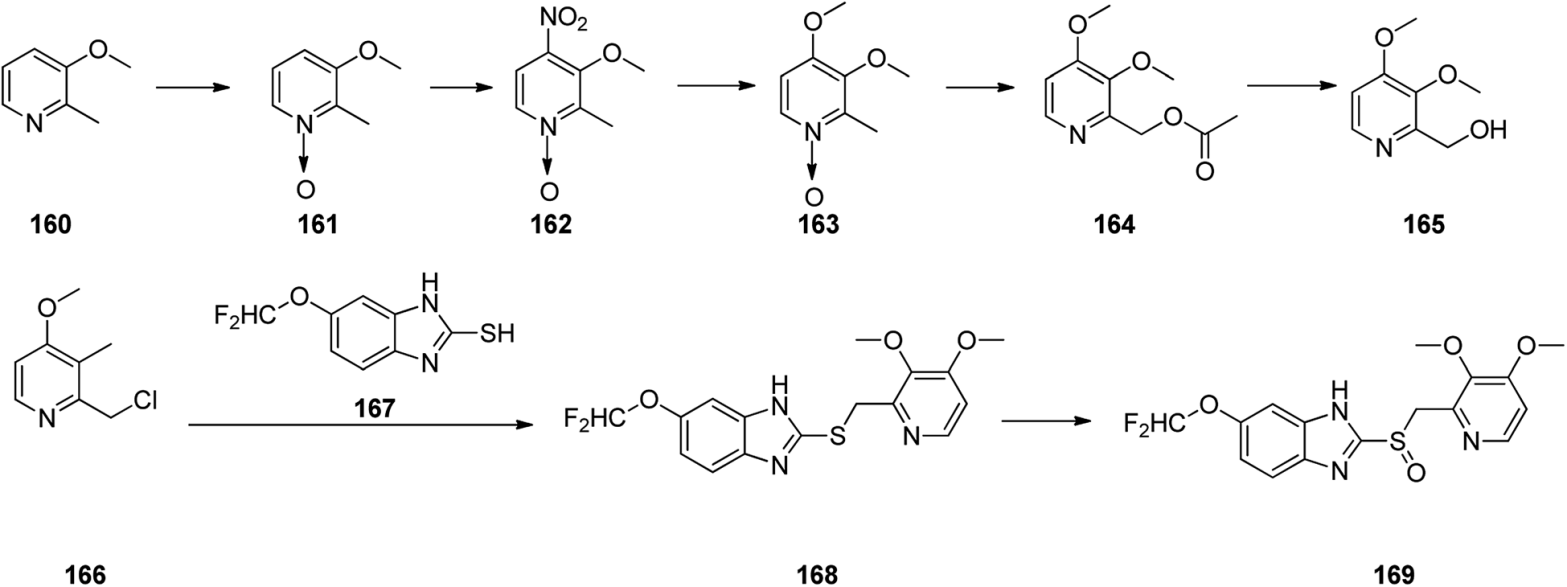
Sequence of reactions for the formation of pantoprazole 169.

Pantoprazole 169 is the third proton pump inhibitor to be propelled for the treatment of peptic acid diseases. Research on pantoprazole commenced in 1985, and commercialized as an approved medication in Germany in 1994.^204^ Form that tine it has been listed as a generic medication and sold under the brand name Protonix among others. Pantoprazole 169 can be synthesized through the same approaches which employed for the above-mentioned synthesis for omeprazole and pantoprazole.^205–207^ Initially, sequential reaction is shown in Scheme 22, including the oxidation of 3-methoxy-2-methylpyridine 160 gave *N*-oxide 161, which upon selective nitration in the fourth position afforded 162. Then the nitro group in 162 was replaced by the methoxy group to give 163 which upon isomerization (Boekelheide rearrangement) furnished 164 which was hydrolyzed to give 2-(hydroxy)-3,4-dimethoxypyridine 165. The latter, was next converted to one of the vital starting materials, 2-(chloromethyl)-pyridine 166. Condensation of the latter with(difluoromethoxy)-2-mercapto-l*H*-benzimidazole 167 led to 2-((pyridin-2-ylmethyl)thio)-1*H*-benzo[*d*]imidazole derivative 168, which, after oxidation, provided the desired drug pantoprazole 169 (Scheme 22). The variation of suggested approach was also revealed in the chemical literature.^208^

**Scheme 23:**
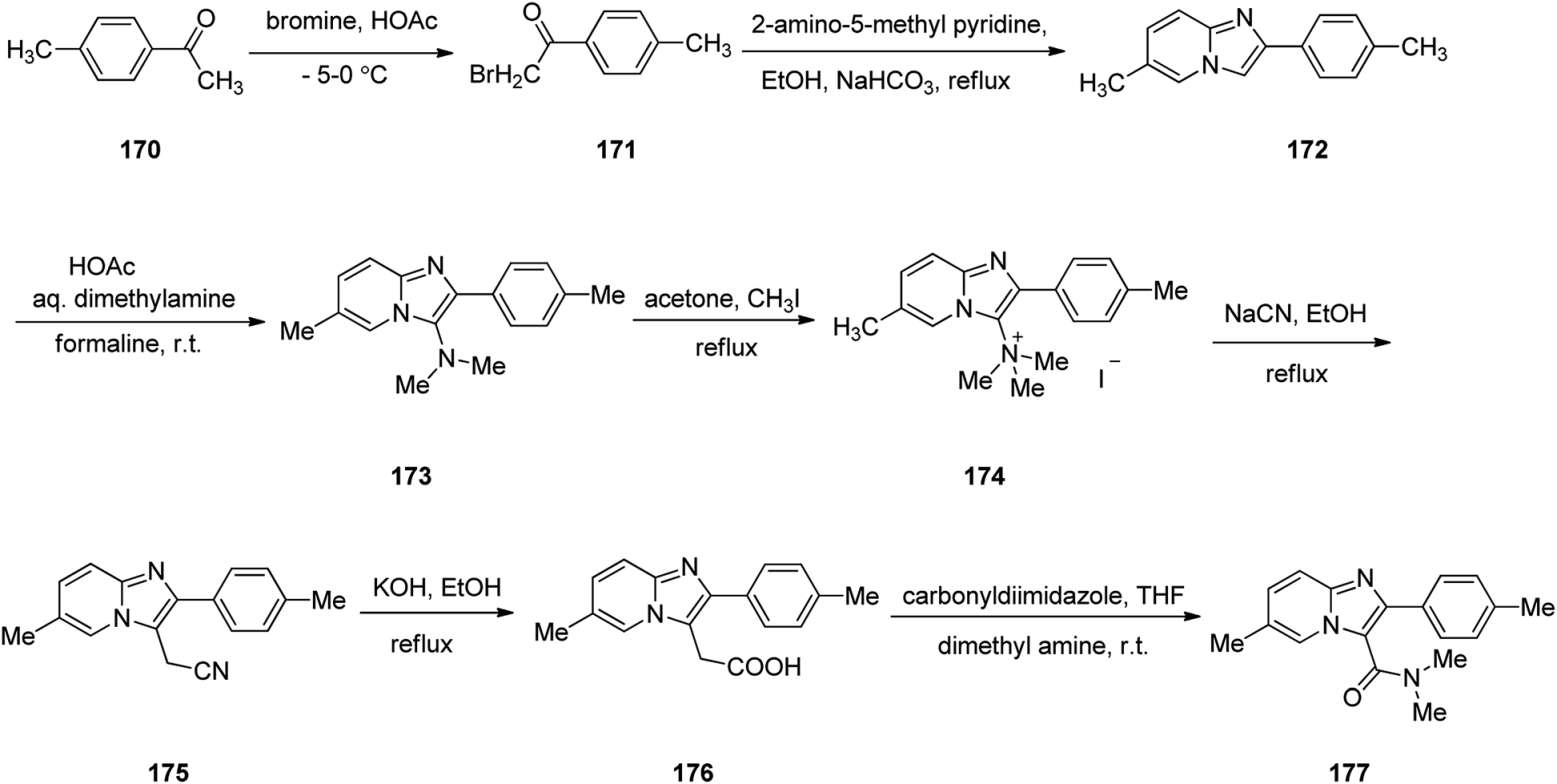
Synthesis of zolpidem 177.

Zolpidem, sold under the brand name Ambien, among others, is a medication primarily used for the short term treatment of sleeping problems. Zolpidem was approved for being prescribed by FDA in 1992 but became accessible as a generic medication in 2007.^209,210^ It decreases the time to sleep onset by about 15 minutes and at larger doses helps people stay asleep longer.^211^ Common side effects include daytime sleepiness, headache, nausea, and diarrhea. Other side effects include memory problems, hallucinations, and abuse.^211^ Zolpidem 177 is a short stand-in, non-benzodiazepine imdazopyridine hypnotic medicine, sold as its tartrate salt.^212^ In addition, zolpidem 177 also have anxiolytic and anticonvulsant properties.^213^ The common synthesis of zolpidem 177 starts by the bromination of 4-methyl acetophenone 170 by bromine in acetic acid to afford its corresponding bromo derivative 171. The latter is condensed with 2-amino-5-methyl pyridine^214^ in the presence of NaHCO_3_ in refluxing EtOH to afford an imidazopyridine intermediate 172 that is subjected to Mannich reaction^215^ of dimethylamine and formaline in acetic acid at room temperature to afford the corresponding *N*,*N*-dimethyl aminoimidazopyridine 173. The latter was then transformed into its quaternary salt 174 upon treatment with CH_3_I in acetone under reflux. The latter was treated with sodium cyanide NaCN, in refluxing ethanol to afford cyano methylimidazopyridine derivative 175. Upon alkaline hydrolysis, the cyano compound 175, provided a pyridine acetic acid compound 176.^216^ Lastly, this essential intermediate 176 treated with carbonyl diimidazole followed by amidation using anhydrous dimethyl amine in THF provided the desired medicine zolpidem 177 (Scheme 23).^217^

**Scheme 24:**
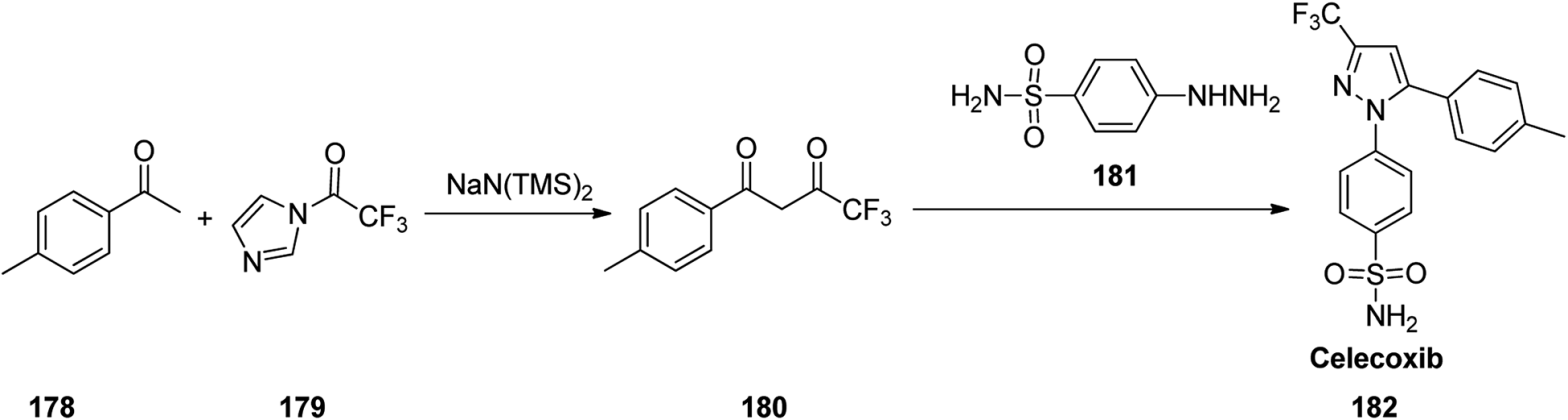
Synthesis of celecoxib 182.

Celecoxib, came to market under the brand name celebrex among others. Celecoxib was patented in 1993 and approved for being used as medicine in 1999.^218^ Celecoxib 182, in fact is a 1,5-diarylpyrazole moiety integrates a sulfonamide or methylsulfonate pharmacophore at *para* position of *N*-aryl segment.^219–221^ It is a selective COX-II inhibitor established by Pfizer company and sold under the brand name of Celebrex®.^222–224^ Various approaches were reported for the formation of pyrazoles mostly based on 1,3-dipolar cycloadditions^225–231^ or condensation reactions^232–236^ as a key stage. It can be prepared by a reaction of 4-methyl-acetophenone 178 with *N*-(trifluoroacetyl)imidazole 179 in the presence of sodium bis(trimethylsilyl) amide to give the 4,4,4-trifluoro-1-(*p*-tolyl)butane-1,3-dione 180.^222^ Upon the reaction of the latter with 4-sulfamoylaphenylhydrazine 181 the desired compound, celecoxib 182 can be obtained (Scheme 24).^237^

**Scheme 25:**
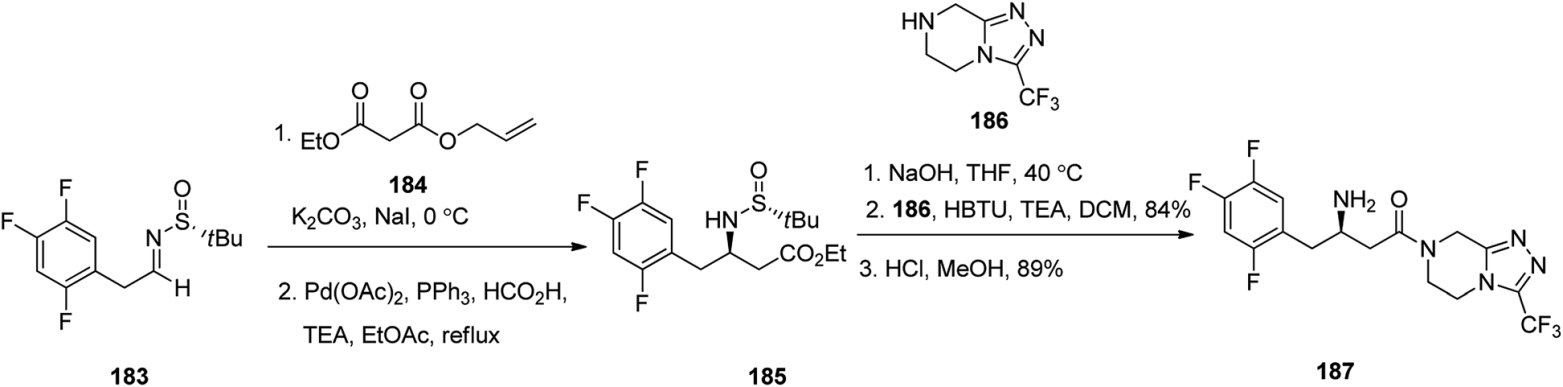
Synthetic pathway to sitagliptin 187.

Sitagliptin 187, which is also in market under the trade name Januvia among other brand names, is a medicine used for the treatment of diabetes mellitus type 2. Sitagliptin 187, formerly named MK-0431, was developed by Merck Company and approved by FDA in 2006.^238^ Sitagliptin 187 was developed by Merck company and approved by FDA in 2006 then, delivered to market as phosphate salt under brand name of Januvia. Sitagliptin 187 is antihyperglycemic drug, is used in the treatment of type II diabetes.^238^ Notably, it is itemized as less favored than metformin or a sulfonylurea in the United Kingdom.^239^ Literature survey from 2005, revealed several synthetic formation for the preparation of sitagliptin 187.^240,241^ In 2017, Suh *et al.*^242^ achieved and reported a highly stereoselective approach for the satisfactory synthesis of sitagliptin. They commenced with *tert*-butyl sulfinyl aldimine 183 that was converted into β-amino-ester 185, as a sole stereoisomer *via* stereoselective enolate addition followed by palladium-catalyzed decarboxylation. Subsequently, the latter was subjected to saponification of the terminal ester and then peptide-like coupling with the piperazine scaffold 186 to afford the desired prescribed drug sitagliptin 187, with high chemical yield and excellent optical purity (Scheme 25).^243^

**Scheme 26:**
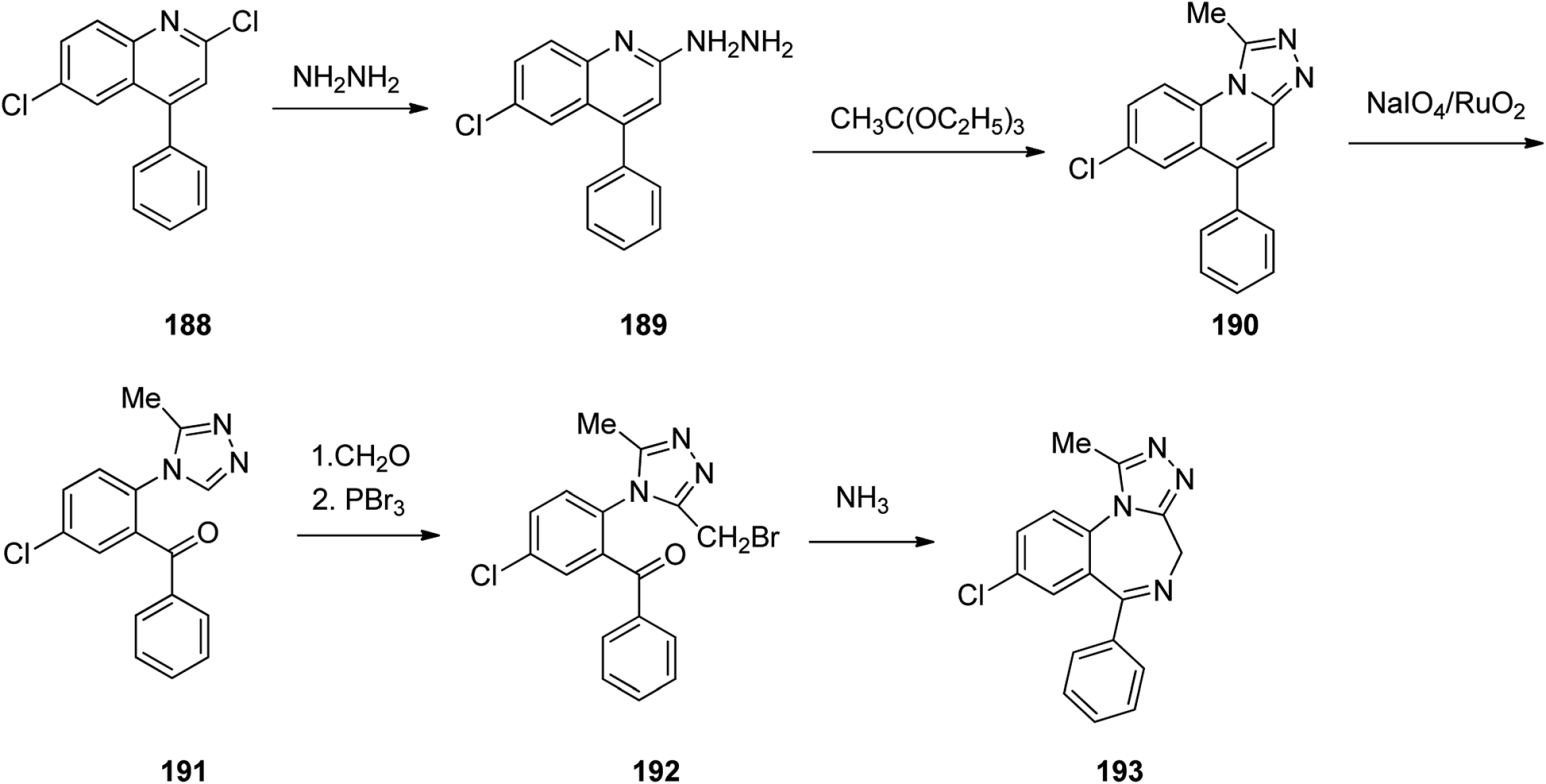
Synthesis of alprazolam 193.

Benzodiazepines are a well-recognized class of compounds with a broad spectrum of central nervous system (CNS) related activities.^244^ They exhibited various kinds of biological potencies, for example antitumoral and anticonvulsive activities.^245^ One of the common intermediates for synthesis of benzodiazepines is aminobenzophenone.^246^ Alprazolam and diazepam are typical of this class of compounds. Benzodiazepines, such as alprazolam and diazepam with approved anxiolytic action and poorly expressed sedative-hypnotic potencies. Furthermore, alprazolam and diazepam are effective in treatment of panic disorders and agoraphobia.^247^ Clinical data proved that alprazolam is also helpful for treatment of depression. Alprazolam is short-lasting sedative taken orally in conditions of nervousness, panic disorders, anxiety which also treats depressive syndrome.^248^ In general, several compounds with promising sedative and toxic activities contain the 1,4-benzodiazepine framework.^249^ This class of active compound is obtainable through ring expansion of quinazolines, by ring contraction of benzoxadiazocines, and *via* synthesis starting from 2-aminobenzophenones.^250^ Alprazolam 193, sold under the brand name Xanax, among other trade names. Alprazolam was patented in 1971 and approved for being prescribed by FDA in 1981.^251–253^ It is most frequently taken orally for the short term controlling of anxiety disorders, specially panic disorder or general anxiety disorder (GAD).^254^ Alprazolam, is actually 8-chloro-1-methyl-6-phenyl-4*H-s*-triazolo[4,3-*a*][1,4]benzodiazepine 193.^255^ The similar strategy that is employed to prepare triazolam can be applied to synthesis alprazolam, with the exclusion which it starts from 2-amino-5-chlorobenzophenone as starting material.^256–258^ Worthy to notice a non-typical approach for the synthesis of alprazolam starting from 2,6-dichloro-4-phenylquinoline, has also been proposed. In this approach, 6-chloro-2-hydrazino-4-phenylquinoline 189 is treated with hydrazine and heating of this mixture with triethyl orthoacetate in xylene results in the corresponding triazole 190*via* the heterocyclization. The latter is subjected to oxidative cleavage utilizing sodium periodate and ruthenium dioxide in an acetone/water as solvent to afford 2-[4-(3′-methyl-1,2,4-triazolo)]-5-chlorobenzophenone 191. Treatment of the latter with formaldehyde, followed by substitution of the resultant hydroxyl group by PBr_3_, affords 2-[4-(3′-methyl-5′-bromomethyl-1,2,4-triazolo)]-5-chlorobenzophenone 192. Replacement of the bromine atom in the latter with an amino group employing ammonia and the impulsive, intermolecular heterocyclization affords alprazolam 193 (Scheme 26).^259–261^

**Scheme 27:**
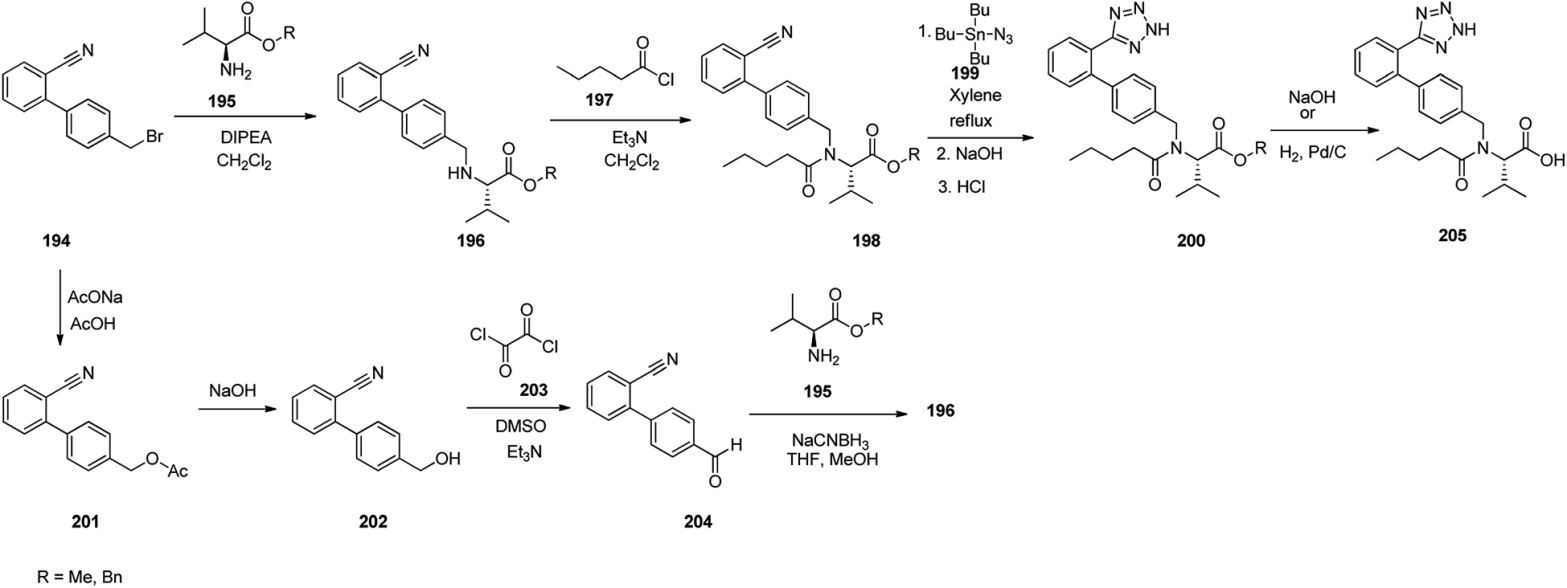
Synthesis of valsartan 205.

Valsartan 205 as the free acid, is a medication commercialized under the brand name ValsaDiovan®. Among other trade names, is prescribed, for the treatment of high blood pressure, heart failure, and diabetic kidney disease. Valsartan was patented in 1990, and came into medical use in 1996. Valsartan 205 is a non-peptide AT-II antagonist.^262–264^ Several approaches for the synthesis of valsartan 205 have been reported.^265,266^ An efficient synthetic pathway started with biphenylbromomethylnitrile 194 which upon alkylation using l-valine methyl or benzyl esters provided amines 196 which subsequently acylated with valeroyl chloride to provide *N*-valeryl derivatives 198. The latter was then refluxed with tributyltin azide in xylene affording tributyltin terazoles which subsequently was conventionally hydrolyzed to afford terazoles 200 in basic or acidic media. At the end, in the case of methyl ester, it was subjected to hydrolysis under basic conditions or in benzyl ester form, it was submitted to hydrogenation over the Pd catalyst, to provide the desired drug, valsartan 205. Alternatively, the same biphenylbromomethylnitrile 201, that was transformed into the corresponding acetate 201 which subsequently hydrolyzed to the corresponding benzyl alcohol 202. The latter under Swern oxidation condition (COCl_2_, DMSO, Et_3_N) was converted to aldehyde 204. Upon reductive amination of the latter using amino component, such as l-valine methyl ester and reductive agent such as sodium cyanoborohydride provided biphenyl nitrile 196. The latter by the same sequence of reactions (196 to 198 to 200) as mentioned above, provided the desired prescribed drug valsartan 205 (Scheme 27).^267^

**Scheme 28:**
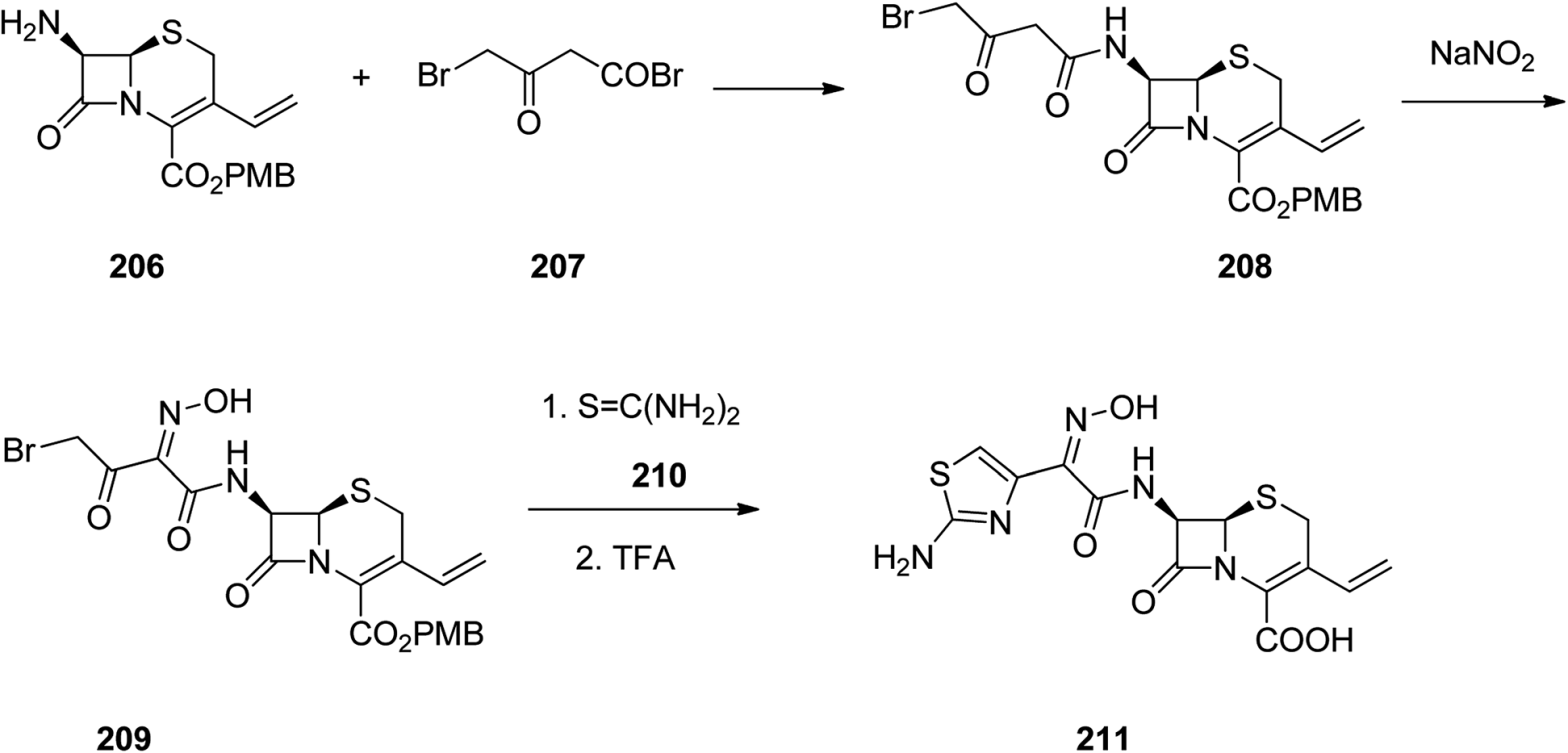
Synthesis of cefdinir 211.

Cefdinir was patented in 1979 approved by FDA, for medical use in 1991, marketed under the brand name Omnicef, among others. It is a third-generation, semisynthetic cephalosporin antibiotic showing a broad spectrum of antibacterial activities.^268–270^ Cefdinir 211 is pinpointed by a vinyl group at C-3 position and a (*Z*)-2-(2-aminothiazol-4-yl)-2-(hydroxyimino)acetyl moiety at C-7 position, leading to a noticeable proliferation in its antimicrobial potency against Gram-positive and Gram-negative bacteria.^271^ It was synthesized through reaction of the primary amine 206 with 4-bromo-3-oxobutanoyl bromide 207 which resulted in the formation of the amide 208. Then, the active methylene group in the latter is nitrosated using sodium nitrite. The initial product impulsively is tautomerized to generate the oxime 209. The bromoketone assortment in this intermediate establishes a classical starting function for assembly of thiazole heterocycles. Reaction of oxime 209 with thiourea resulted in the formation of an aminothiazole moiety.^272^ Thus in this way the antibiotic cefdinir can be synthesized 211 (Scheme 28).^269,272–274^

**Scheme 29:**
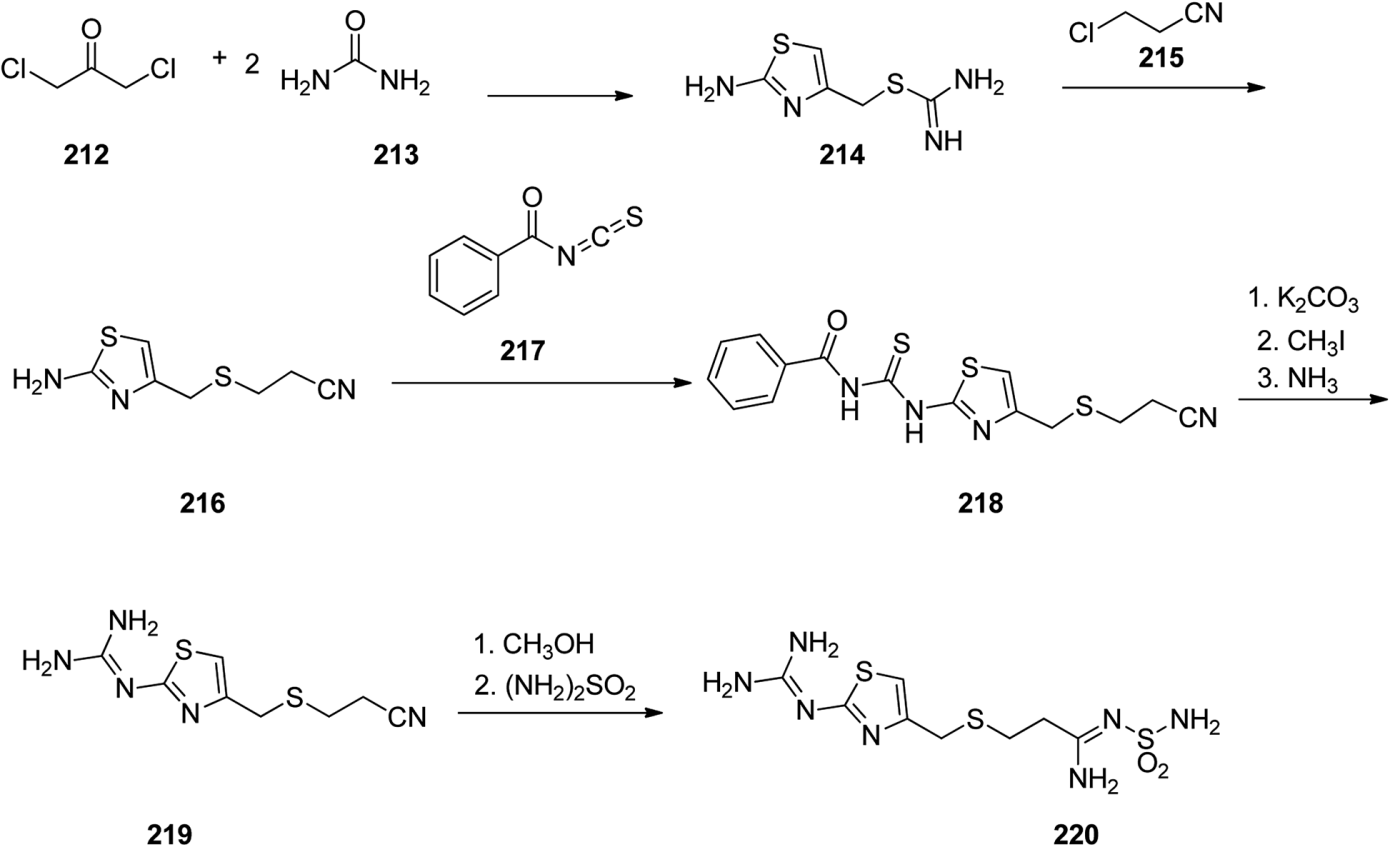
Synthesis of famotidine 220.

Famotidine 220, commercialized also under the trade name Pepcid among other brands, is a medicine that decreases stomach acid production. Famotidine 220 was patented in 1979 and passed clinical trials, approved by FDA in 1985.^275,276^ Fomatidine which is actually, 3-[[2-[(aminomethyl)amino]-4-thiazolyl]methyl]thio]-*N*-(aminosulfonyl)propanimidamide 220, can effectively be prepared in accordance with synthetic partway as illustrated in Scheme 29. 1,3-Dichloroacetone was reacted with two molecules of thiourea during which a thiazol ring is formed and the chlorine atom is substituted, providing an intermediate, 2-amino-5-chlormethylthiazol 214. The latter upon treatment with 2-chlorpropionitrile affords *S*-(2-aminothiazol-4-yl-methyl)-2-cyanoethane 216. The latter was then treated with benzoylizthiocyanate to afford benzoylthiourea derivative 218. This compound 218 was initially subjected into *S*-methylation using methyliodide and further submitted to cleavage by ammonia to afford 3-[[[2-(aminomethyl)amino]-4-thiazolyl]-methyl]thio]ethylcyanide 219. Sequential methanolysis of the nitrile group followed by reaction of the resulting iminoether with sulfonamide gives famotidine 220.^277–282^

**Scheme 30:**
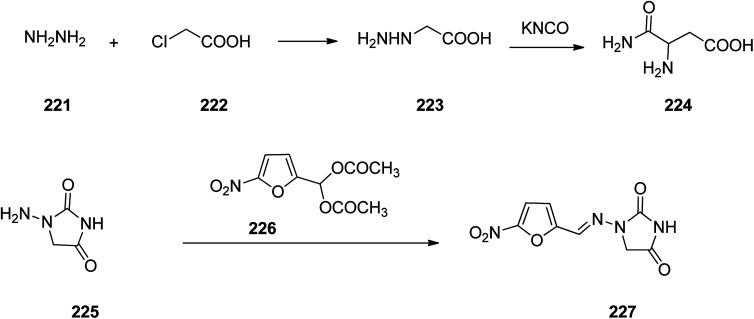
Synthesis of nitrofurantoin 227.

Nitrofurantoin 227, which was commercialized in 1953 under the trade names Macrobid®, Macrodantin®, and Furadantin®, is an antibiotic employed for the treatment of bladder and urinary tract infections. In spite of the development of a wide variety of new generation of antibiotics, nitrofurantoin vestiges a forefront for the treatment of easy urinary tract (pyelitis, pyelonephritis, cystitis, urethritis).^283^ Nitrofurantoin, 1-(5-nitrofurfurylidenamino)hydantoin 225, can deductively be prepared from hydrazinoacetic acid 223, that is provided upon treatment of chloroacetic acid with hydrazine. Treatment of the latter with potassium cyanate affords the semi-carbazidoacetic acid 224 that upon heating was cyclized into 1-aminoidantoin 225. When the latter was reacted with diacetylacetal of 5-nitrofurfurol the desired prescribed drug, nitrofurantoin 227 was obtained (Scheme 30).^284–298^

**Scheme 31:**
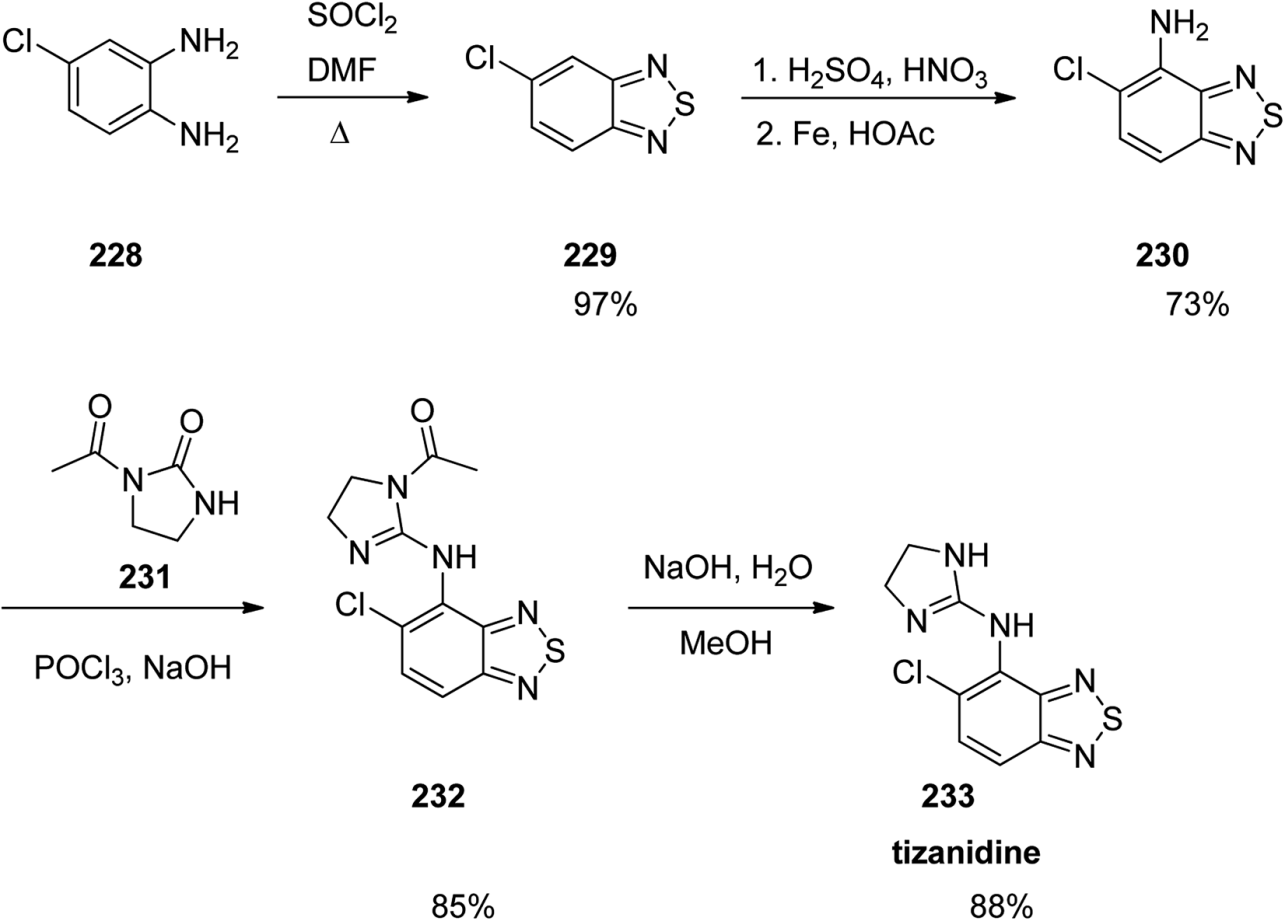
Synthesis of tizanidine 233.

Tizanidine 233, came to market under the trade name Zanaflex among other brand names. It is a medicine which prescribed for the treatment of muscle spasticity because of spinal cord injury or multiple sclerosis.^289^ Tizanidine was approved for being prescribed by FD in 1996.^289^ It functions similar to baclofen or diazepam.^290^ Tizanidine is actually a substituted-1,3-benzothiadiazole 233. Treatment of an aromatic diamine 228 with SOCl_2_ in dimethylformamide gave the corresponding benzothiadiazole 229. The latter upon selective nitration followed by an Fe-mediated reduction gave the respective aniline 230 that is subjected to a nucleophilic substitution with 2-chloro-3,4-dihydroimidazole (produced *in situ via* the reaction of the urea 231 and POCl_3_). Elimination of the acetate group of the latter under basic conditions led to the desired medication tizanidine 233 (Scheme 31).^291^

**Scheme 32:**
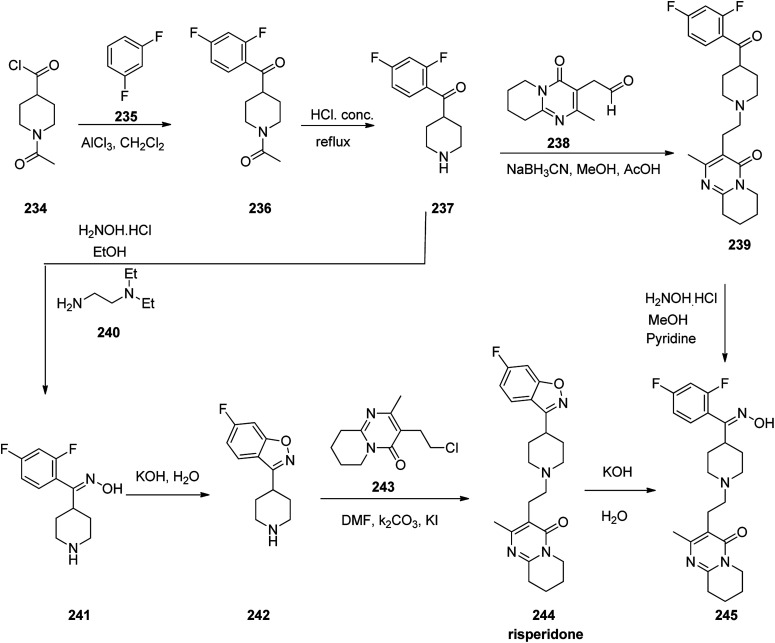
Synthesis of risperidone 244.

Risperidone 244, approved and came to market in 1993 under the trade name Risperdal among others.^292^ Risperidone is also active for Alzheimer's dementia, and substance abuse disorders.^293–298^ Risperidone 244 was prepared starting from 1-acetyl-4-piperidine-carbonyl chloride 234 that was employed to acylate 1,3-difluorobenzene 235 in CH_2_Cl_2_ in the presence of AlCl_3_ as Lewis acid. This reaction afforded 1-(4-(2,4-difluorobenzoyl)piperidin-1-yl)ethan-1-one 236. The protecting acetyl group of the latter was cleaved *via* hydrolysis in 6 N HCl under reflux condition which afforded (2,4-difluorophenyl)(piperidin-4-yl) methanone 237. The resultant product 237 was then transformed into respective oxime 241 upon treatment with NH_2_OH/HCl in EtOH using *N*,*N*-diethylenethanamine. The latter was then cyclized to 6-fluoro-3-(piperidin-4-yl)benzo[*d*]isoxazole 242 using 50% KOH solution of H_2_O under reflux condition. Finally, the latter was alkylated with 3-(2-chloroethyl)-2-methyl-6,7,8,9-tetrahydro-4*H*-pyrido[1,2-*a*]pyrimidin-4-one 243 upon heating at 85–90 °C in DMF using Na_2_CO_3_ and KI to furnish the desired product, risperidone 244.^299,300^ Alternatively, compound 237 was transformed into 244 upon reductive alkylation of (2,4-difluorophenyl)(piperidin-4-yl)methanone 237 with aldehyde 238 using NaBH_3_CN which afforded compound 239, which was reasonably transformed into oxime 245 and further to the desired target compound, risperidone 244 (Scheme 32).^301^

**Scheme 33:**
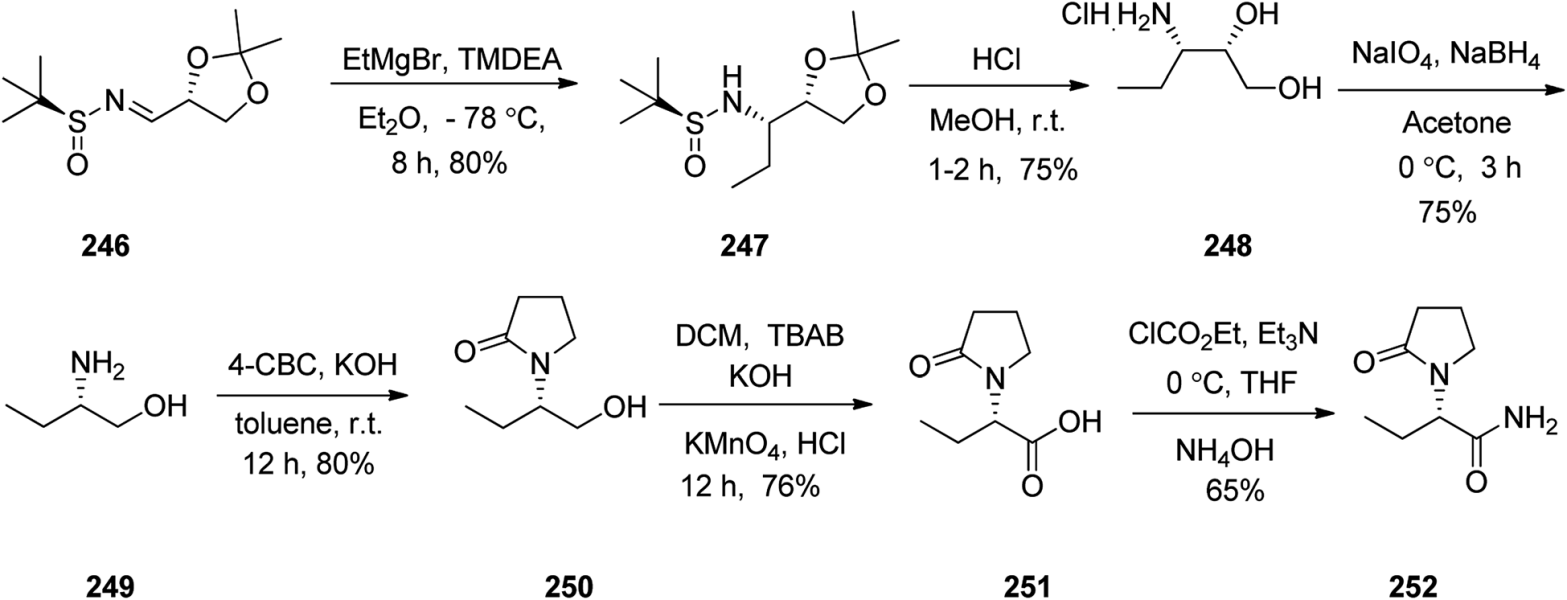
Synthesis of (−)-levetiracetam 252.

Levetiracetam 252, marketed under the trade name Keppra was approved as a medication for the treatment of epilepsy in 1999. An enantioselective synthesis of (−)-levetiracetam 252 was achieved and reported in six steps commencing from multipurpose novel optically active *N*-sulfinimine 246. In this strategy, the key step is asymmetric 1,2-addition of ethylmagnesium bromide (EtMgBr) to optically active *N*-sulfinimine prepared from (*R*)-glyceraldehyde acetonide and (*S*)-*t*-BSA, which afforded the respective sulfonamide 247 in high diastereoselectivity. Concurrent deprotection and deacetylation with subsequent cleavage using sodium periodate followed by reduction afforded ββ-amino alcohol 249. Subsequent reactions provided the targeted compound levetiracetam 252. The addition of the Grignard reagent to the imines 246 in tetrahydrofuran at −78 °C followed by deprotection of the *t*-butylsulfinyl group and 1,3-dimethylacetalin gave compound 247 which in acidic media (MeOH·HCl) was converted into the respective ββ-aminodiol 248. The latter, upon oxidation followed by reduction of amino diol using sodium periodate/sodium borohydride afforded the corresponding ββ-amino alcohol 249.^302^ The latter upon treatment with 4-chlorobutyryl chloride^303^ afforded 2-pyrrolidonealcohol 250. The latter upon oxidation with potassium permanganate gave 251, which in turn upon amidation provided the targeted (−)-levetiracetam 252. The spectral data of compound 252 was compared with those of previously reported in the literature and found being identical (Scheme 33).^304^

**Scheme 34:**
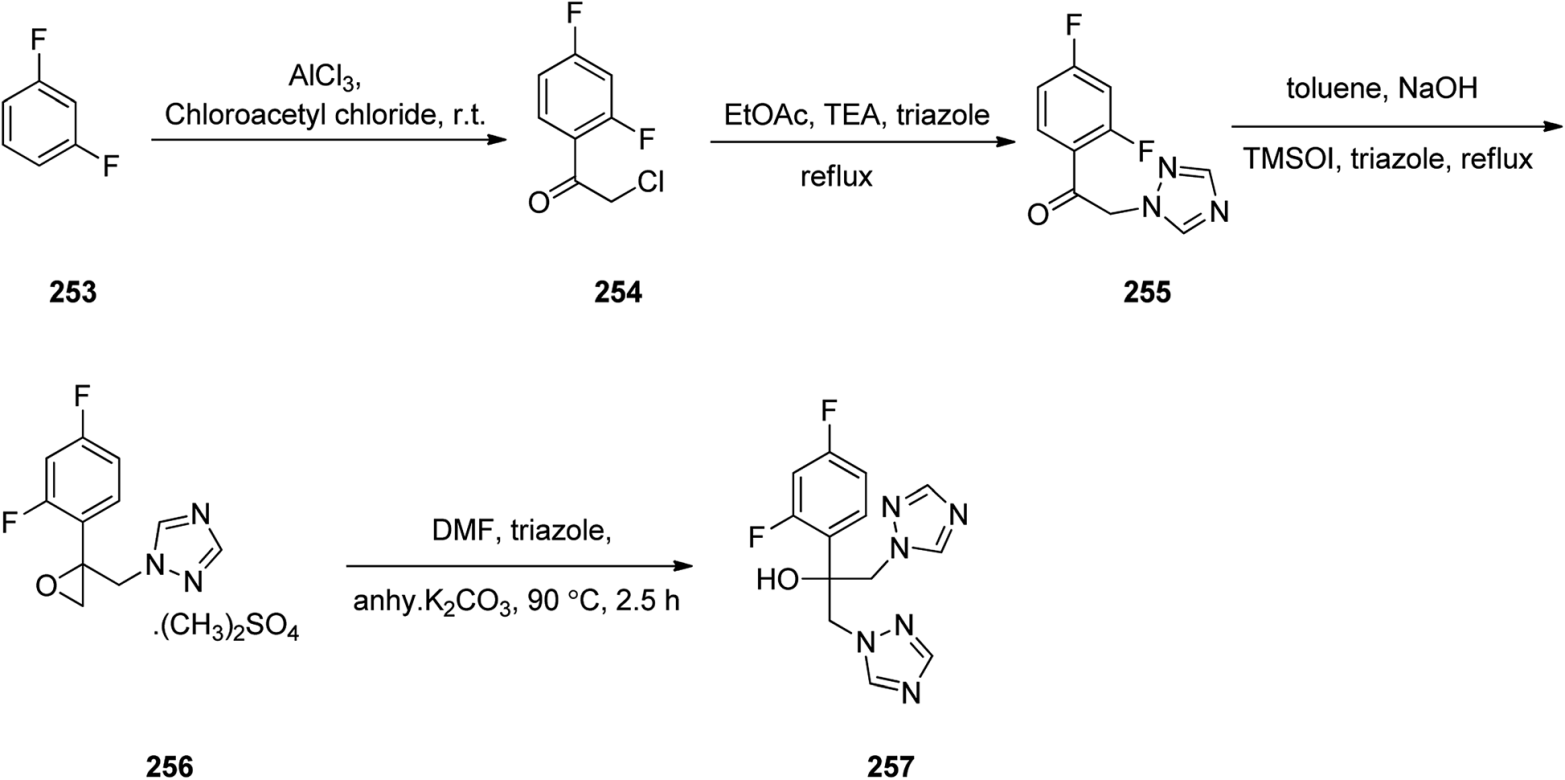
Synthesis of fluconazole 257.

Fluconazole 257, is actually, α-(2,4-difluorophenyl)-α-(1*H*-1,2,4-triazol-1-yl-methyl)-1*H*-1,2,4-triazol-1-ethanol. Fluconazole was patented in 1981.^305^ It is an antifungal medicine prescribed for several fungal infections. Fluconazole was synthesized following a pathway as depicted in Scheme 34. 1,3-Difluorobenzene 253 reacted with chloroacetyl chloride in presence of aluminum chloride *via* Friedel–Crafts acylation reaction provided 2-chloro-1-(2,4-difluoro-phenyl)ethanone 254.^306^ Chloro compound 254 underwent nucleophilic substitution with 1,2,4-triazole in CH_3_COOEt in the presence of Et_3_N under reflux gave 1-(2,4-difluoro-phenyl)-2-[1,2,4]triazol-1-yl-ethanone 255. The latter upon treatment with trimethylsulfoxonium iodide in the presence of catalytic quantity of cetyltrimethylammonium bromide gave respective epoxy derivative 256 which upon the reaction with triazole under basic condition furnished fluconazole 257 (Scheme 34).^307^

**Scheme 35:**
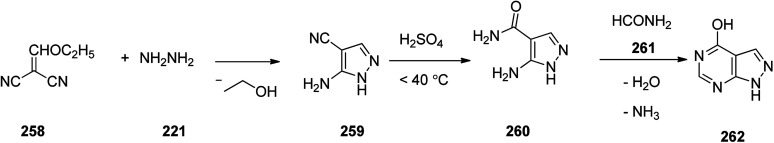
Synthesis of allopurinol 262.

Allopurinol 262, is actually 1,5-dihydro-4*H*-pyrazolo[3,4-*d*]pyrimidin-4-one. It is marketed under the trend name Zyloprim and approved for being prescribed in the United States in 1966. It is a medication used to decrease high blood uric acid levels.^308^ Allopurinol 262, can be synthesized *via* a three steps pathway involving initial condensation of hydrazine 221 with ethoxymethylenemalononitrile 258 to afford 3-amino-4-cyanopyrazole 259, which, upon hydrolysis in acidic media (H_2_SO_4_) gave the corresponding amide 260. In last step, the latter was reacted with excess of formamide 261 under heating to furnish the desired medicationin, allopurinol 262 (Scheme 35).^309^

**Scheme 36:**
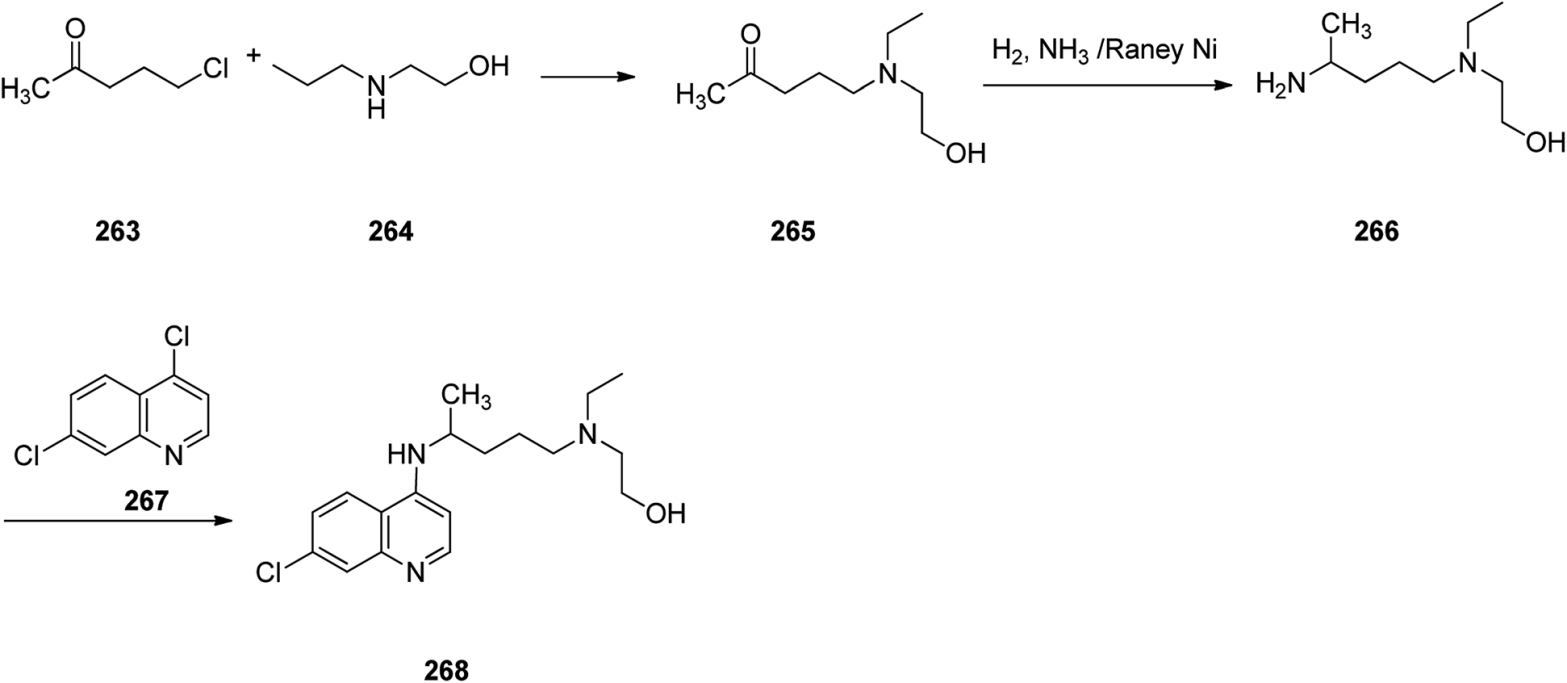
Synthesis of hydroxychloroquine 268.

Hydroxychloroquine (HCQ) 268, which came to market under the trade name, Plaquenil, is a medication used for both prevention and treatment of certain kinds of malaria (chloroquine-sensitive malaria). Hydroxychloroquine was approved for being prescribed in the United States in 1955. Sometimes it is prescribed for the treatment of rheumatoid arthritis, lupus, and porphyria cutanea tarda.^310^ Interestingly, it is being used as an experimental medication for possible treatment for notorious coronavirus disease 2019 (COVID-19) which has very recently broken out and turned pandemic, in short period of time.^311^ Hydroxychloroquine 268, which is actually 7-chloro-4-[4-[ethyl(2-hydroxyethyl)amino]-1-methylbutylamino]quinoline 268, was synthesized in three steps starting from commercially available 1-chloro-4-pentanone as depicted in Scheme 36. Reaction of 1-chloro-4-pentanone with 2-ethylaminoethanol afforded the respective aminoketone 265 that was subjected to reductive amination to furnish 4-[ethyl(2-hydroxyethyl)amino]-1-methyl-butylamine 266. Reaction of the latter with 4,7-dichlroquinoline 267 gave the desired hydroxychloroquine 268.^312,313^

Pioglitazone 275, is a medication prescribed for the treatment of type 2 diabetes.^314^ It decreases insulin resistance in adipose tissue and liver.^315^ In addition, pioglitazone shows positive influences on lipid metabolism, regulate blood pressure, and endothelial function.^316–322^

**Scheme 37:**
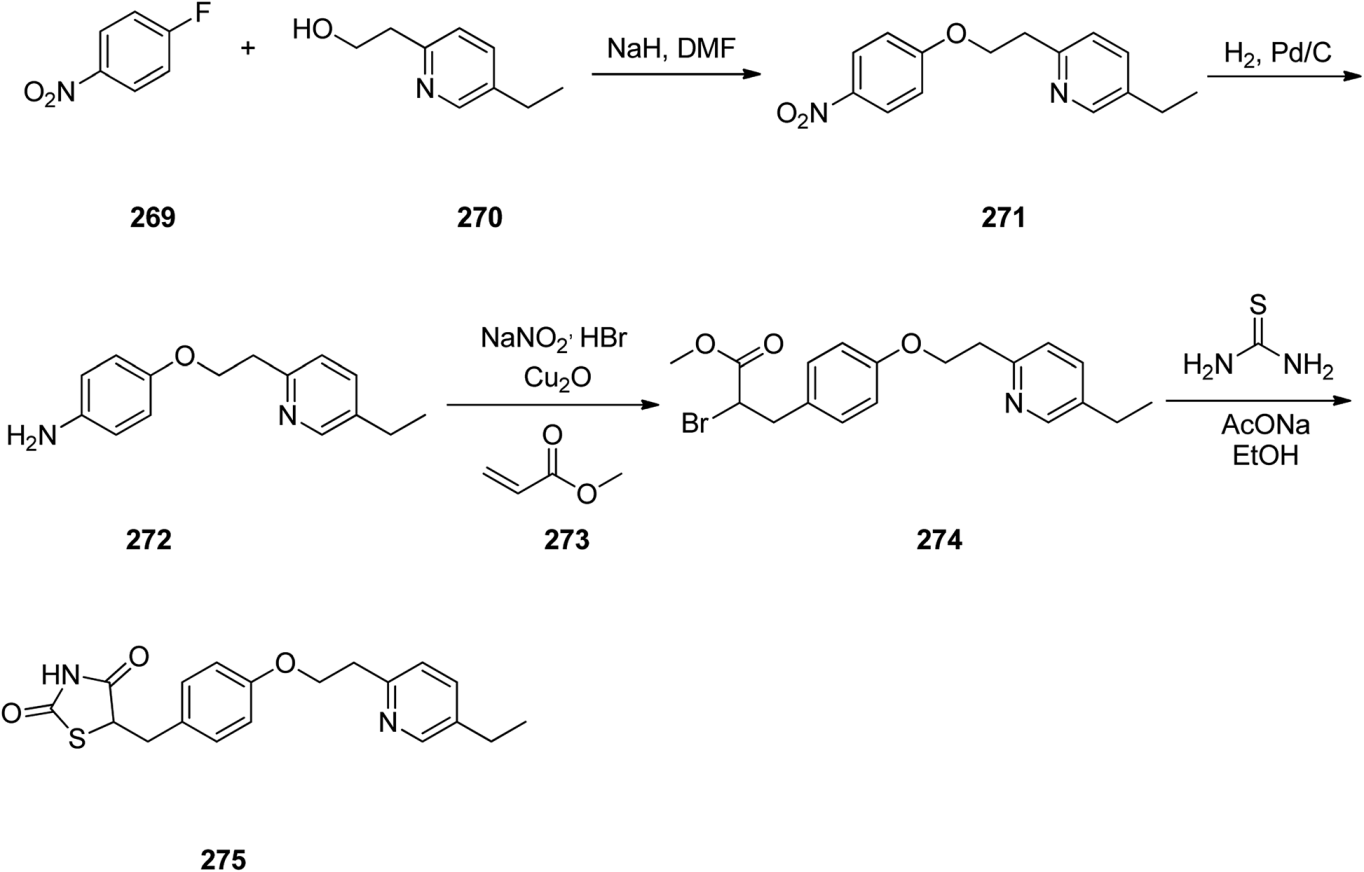
Synthesis of pioglitazone 275.

Pioglitazone 275 was synthesized *via* a five step pathway, commencing from commercially available 1-fluoro-4-nitrobenzene 269. Condensation of the latter with 2-(5-ethyl-2-pyridyl)ethanol 270 provided pyridylethoxybenzene 271 that subsequently was hydrogenated using Pd on charcoal as catalyst to provide the anticipated aromatic amine 272. The latter upon diazotization in a mixture of acetone/methanol followed the workup with HBr, and coupling with methylacrylate in the presence of Cu_2_O (the Meerwein arylation) provided the methyl 2-bromo-propanoate derivative 274. The latter was subjected into cyclocondensation with thiourea, gave an imino compound as intermediate which upon hydrolysis provided the desired target pioglitazone 275 (Scheme 37).^323,324^

Lansoprazole 283, which reduces stomach acid, was patented in 1984 and approved for medical use in 1992.^195^ It is also known under the trade name Prevacid and prescribed for the treatment of peptic ulcer disease as well as Zollinger–Ellison syndrome.^194^ The most common method for the formation of lansoprazole reported by Nohara and Maki.^325^

**Scheme 38:**
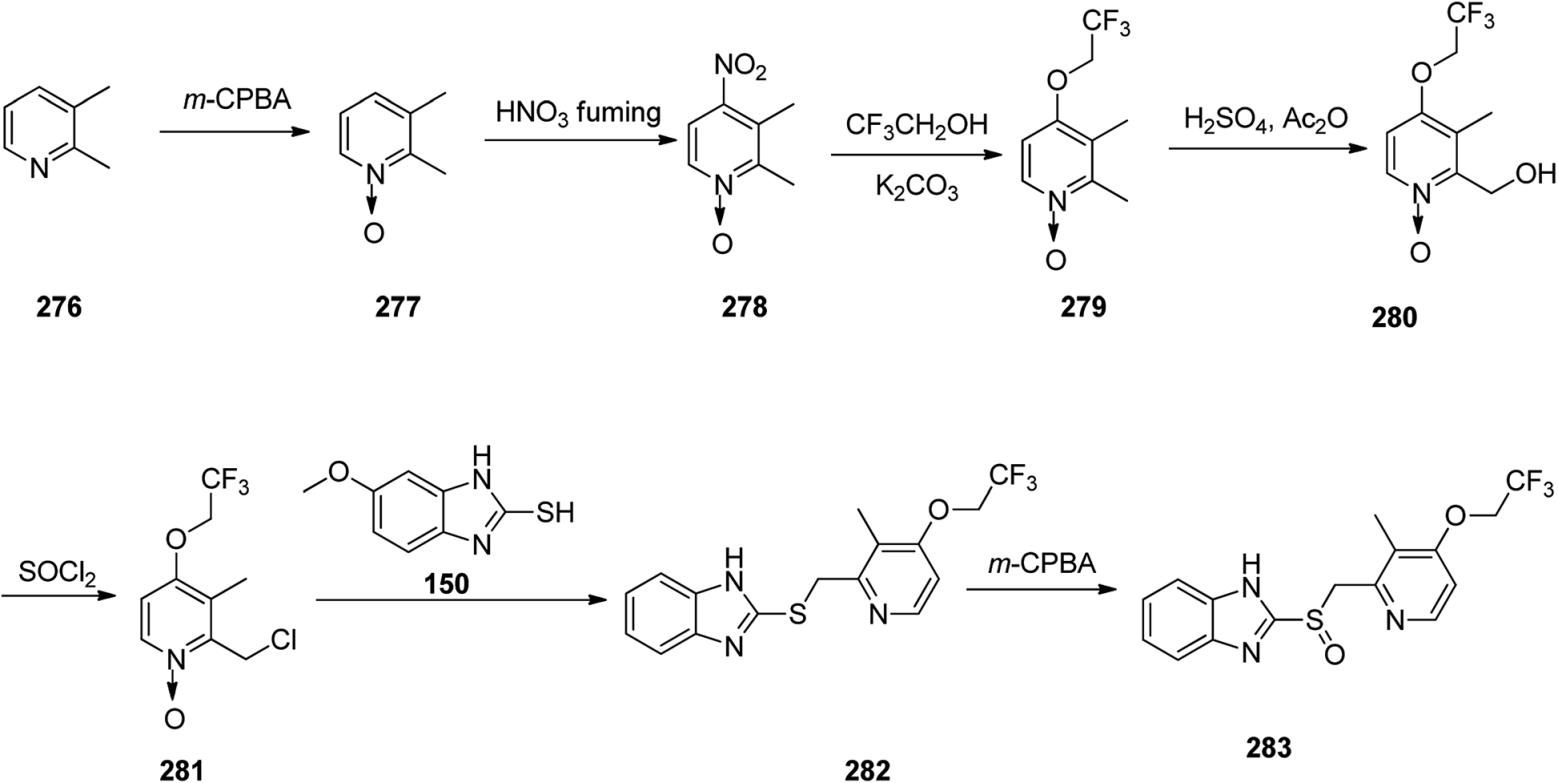
Synthesis of lansoprazole 283.

This method was improved later and patented.^326–328^ The synthetic pathway for lansoprazole is depicted in the Scheme 38. In principle, it is the synthetic route of omeprazole, only divergent in details and characteristics, for instance, instead of 2,3,5-collidine as a starting material, 2,3-lutidine 276 was chosen, and the methoxy moiety in the fourth position of pyridine ring was changed by the 2,2,2-trifluoroethoxy moiety.

**Scheme 39:**
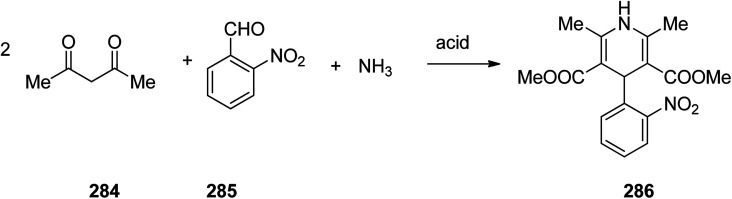
Synthesis of nifedipine 286.

Nifedipine 286 is a famous medication used to regulate high blood pressure. It is also a calcium channel blocker of the dihydropyridine type. Nifedipine 286 was patented in 1967 and commercialized by Hofmann La Roch in 1981 under the trade name of Adalat among others. It is taken orally and comes in fast and slow release formulations.^329^ Nifedipine, actually is dimethyl ether 1,4-dihydro-2,6-dimethyl-4-(2′-nitrophenyl)-3,5-piridindicarboxylic acid 286, which is produced in large scale *via* Hantzsch 1,4-dihydropyridines (1,4-DHPs) synthesis. A multicomponent reaction, comprising two molecules of a β-dicarbonyl compound-methyl acetoacetate, 2-nitrobenzaldehyde and ammonia in acidic media and in one pot fashion produces the desired nifedipine 286. The sequence of the generation of intermediate has not been completely recognized (Scheme 39).^330–333^

**Scheme 40:**
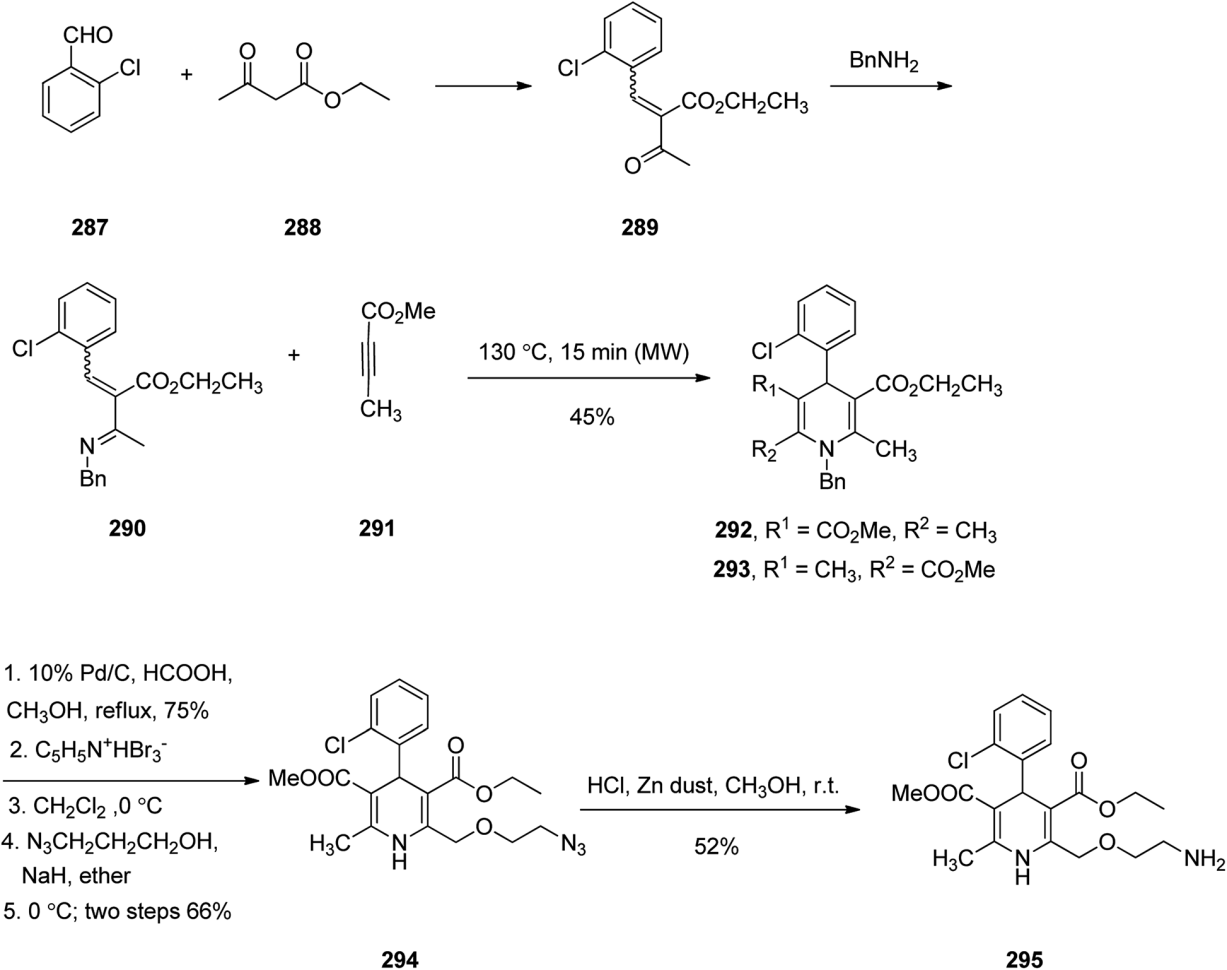
Synthesis of amlodipine 295.

Amlodipine 295, is an approved drug used to treat high blood pressure and coronary artery disease. It is also presently sold under the brand name Norvasc.^334^ Amlodipine acts partially by swelling the size of arteries. It is in fact an efficient calcium channel blocker of the dihydropyridine category such as nefidipine which is a 1,4-dihydropyridine Ca^2+^ channel blockers and substantial antihypertensive drug.^335,336^ Amlodipine 295 was first patented in 1982 but approved as the prescribed drug in 1990.^337^ 1,4-Dihydropyridines are frequently synthesized *via* an approach, explored by Hantzsch^338^ in 1882. This name reaction involves a simple procedure and straightforward isolation of product. Amlodipine 295 was synthesized as depicted in Scheme 40. Initially, the reaction of 2-chlorobenzaldehyde 287 with ethyl acetoacetate 288 under conventional heating gave the expected Knoevenagel product 289 as *E*,*Z* mixture in 70% yield. Then, compound 289 was reacted with benzylamine in presence of anhydrous MgSO_4_ under the microwave irradiation (MWI) at 70 °C to furnish imine compound 290 which was not isolated but subsequently the methyl butynoate 291 was added to the reaction mixture in the same vessel and exposed again to MWI to obtain Aza–Diels–Alder products 292 and 293 as a mixture in 45% combined yield. The reverse phase HPLC analysis of the reaction mixture confirmed that compound 292 was formed regioselectively over 293 with ratio of 7 : 3. The structure of chief product 292 was elucidated by its spectroscopic data with those of authentic sample prepared *via* classical Hantzsch reaction followed by *N*-benzylation. Next, the side chain at C-2 in amlodipine 295 was successfully introduced *via* a procedure patented by Pfizer company.^339^ Product 292 obtained by Aza–Diels–Alder reaction upon treatment with formic acid followed by refluxing the reaction mixture in the presence of Pd/C resulted in the removal of benzyl group which was then upon bromination with pyridium tribromide gave the corresponding bromo compound. It was found that only C-2 position was selectively brominated over C-6 position.^340^ Reaction of the bromo compound with 2-azidoethanol in the presence of sodium hydride provided compound 294 in two steps good overall yield. At the end, reduction of azido group using zinc dust to amine was achieved to give the desired amlodipine 295 in satisfactory yield.^341^

**Scheme 41:**
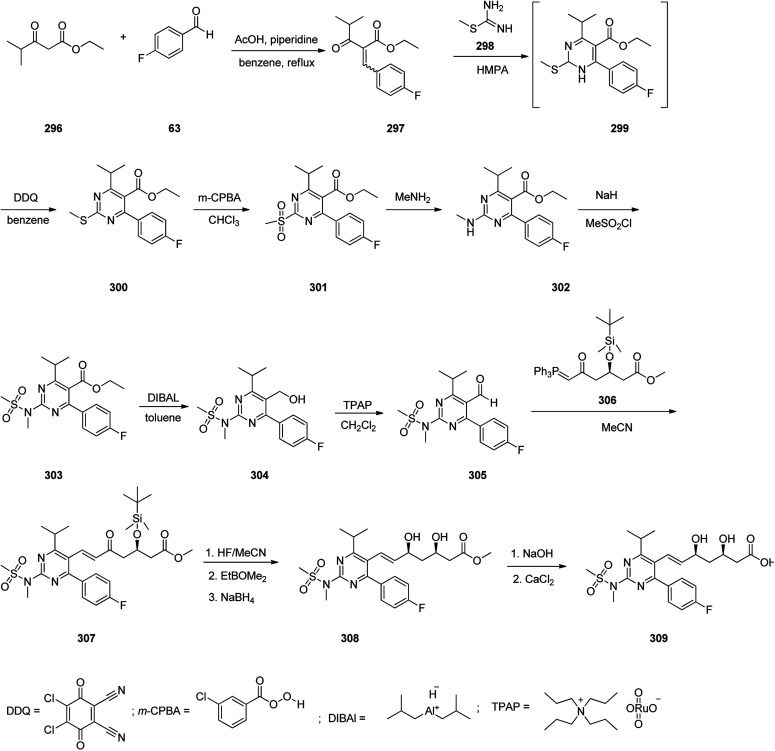
Synthesis of rosuvastatin 309.

Rosuvastatin 309, is drug that inhibits the synthesis of cholesterol and promote the production of LDL-binding receptors in the liver. It was first patented in 1991 and marketed in 2003 under brand name of Crestor.^342^ A common synthesis of rosuvastatin commences from reaction of ethyl isobutyrylacetate 296 with *p*-fluorobenzaldehyde 63 that afforded the corresponding Knoevenagel condensation adduct benzylidene keto ester 297. The latter was upon cyclocondensation with *S*-methylisothiourea 298 in hexamethylphosphoramide (HMPA) at 100 °C provided the intermediate 299, which, without further purification, was subjected to dehydrogenation-aromatization with 2,3-dichloro-5,6-dicyano-*p*-benzoquinone (DDQ) to provide the corresponding pyrimidine derivative 300. The sulfur scaffold in the resultant *S*-methyl pyrimidine 300 was oxidized employing *m*-chloroperbenzoic acid (*m*-CPBA) in CHCl_3_ to provide methylsulfonylpyrimidine 301. The latter was then converted into a methylamino derivative 302 through reaction with methylamine ethanol solution followed by treatment with methyl sulfonyl chloride in the presence of NaH to furnish the sulfonamide 303. Upon reduction of the ester group of the latter using diisobutylaluminium hydride (DIBAL) in toluene the corresponding primary alcohol 304 was obtained which upon oxidation using tetrapropylammonium perruthenate (TPAP) provided the respective aldehyde 305. Then, the latter was subjected to Wittig reaction with the optically active ylide-(3*R*)-3-(*tert*-butyldimethylsilyloxy)-5-oxo-6-triphenylphosphoranylidene hexanoate 306 in boiling acetonitrile to provide heptanoate 307 that was deprotected using hydrofluoric acid in acetonitrile, and the intermediate was reduced to using sodium borohydride in tetrahydrofuran to provide the ester 308 as *syn*-diol, regioselectively, typical for Wittig reaction. At last the latter treated with aqueous sodium hydroxide to provide the respective sodium salt that was converted into desired calcium salt, rosuvastatin 309 (Scheme 41).^343,344^

**Scheme 42:**
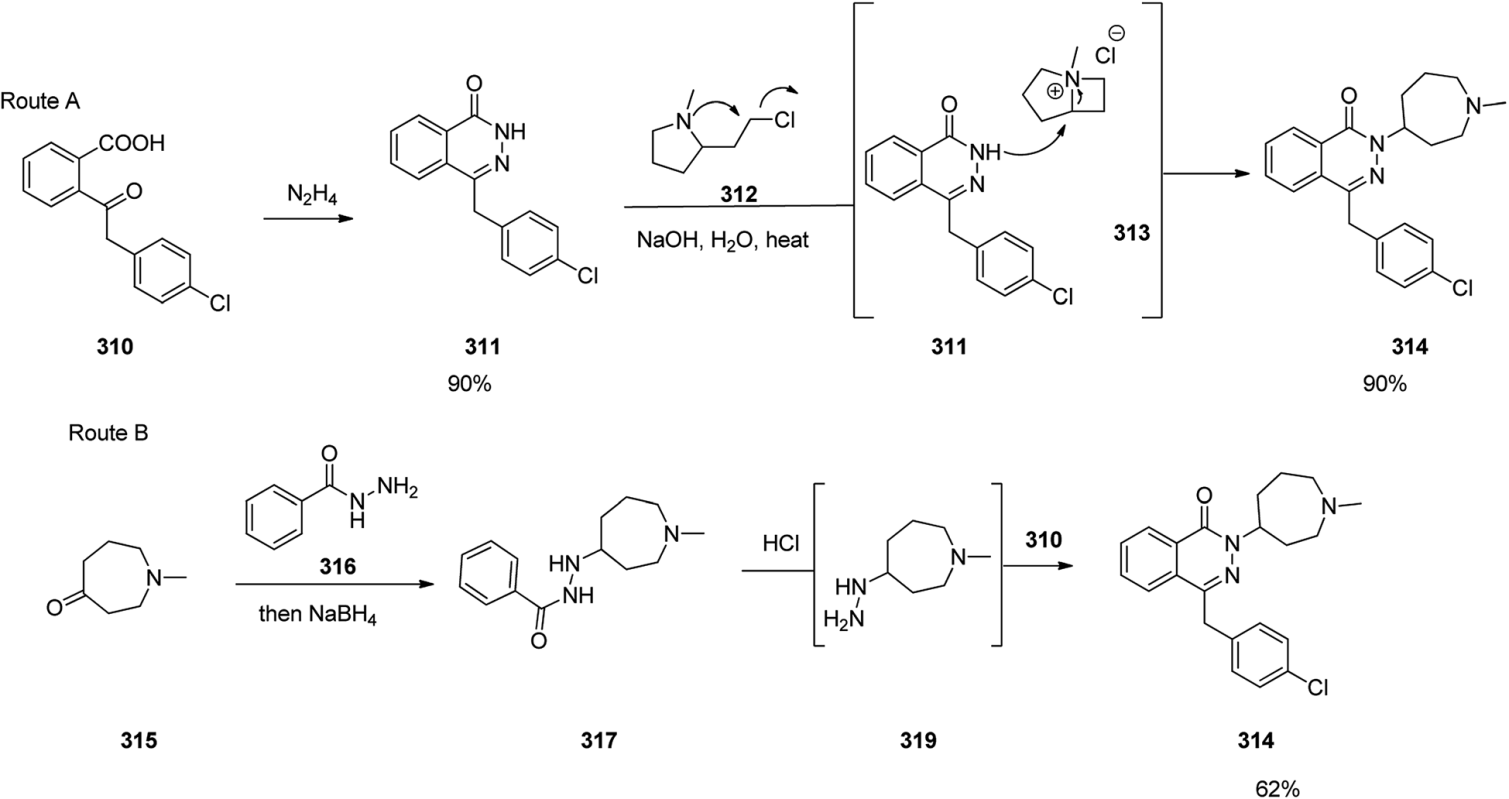
Syntheses of azelastine 314.

Azelastine is a medication which is primarily used as a nasal spray to treat allergic rhinitis (hay fever).^345^ Azelastine was first patented in 1971 and came into medical use under the trade name of Optivar in 1986.^346^ A brief synthesis of azelastine 314 was achieved and reported as shown in Scheme 42. It invoved the reaction of hydrazine with keto-acid 310 to give phthalazinone 311. The latter was reacted with 2-(2-chloroethyl)-*N*-methylpyrrolidine 312 in hot aqueous NaOH provided azelastine *via* a fascinating ring expansion. This ring expansion apparently proceeds *via* the intermediacy of [3.2.0]-framework 313 to afford the desired target azelastine 314 (Scheme 42, route A).^347^ On the other hand, the same keto-acid 310 can undergo condensation with substituted hydrazine 318 that was provided from an acid-mediated hydrolysis of acyl hydrazide 317 to give the desired azelastine 314 (Scheme 42, route B). Notably, by using a solid hydrazide 316 instead of volatile hydrazine this route is longer but it is a safer alternative at large scale production.

**Scheme 43:**
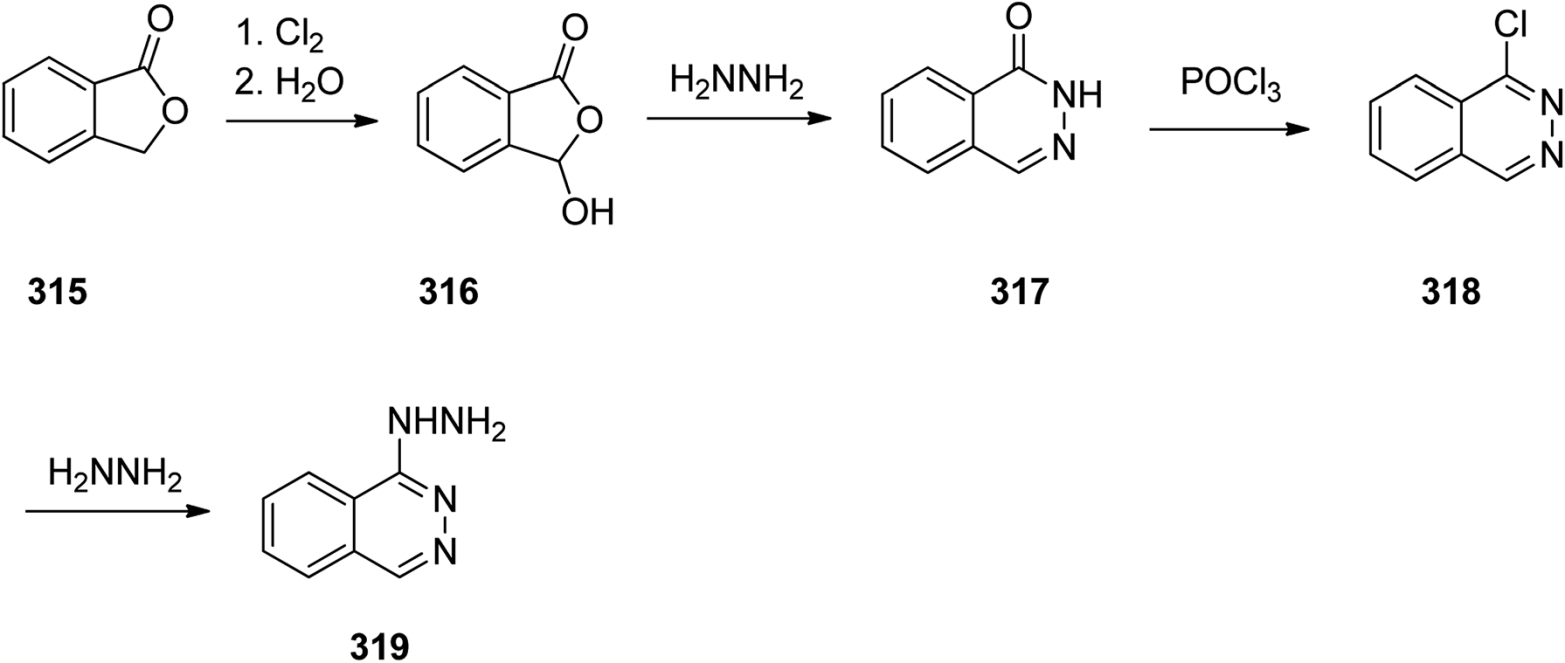
Synthesis of hydralazine 319.

Hydralazine 319, is a medication used to treat high blood pressure and heart failure.^348^ Hydralazine 319, is also sold under the trade name Apresoline.^349^ Hydralazine, 1-hydrazinonaphthalazine 319, was synthesized *via* four steps reaction stating from the oxidative chlorination of phthalide 315 with concurrent hydrolysis of product that leads to hydroxyphthalide 316. The latter was reacted with hydrazine hydrate to give phthalazone 317, which upon treatment with phosphorous oxychloride to give 1-chloroph-thalazine 318. In the last steps substitution of the chlorine atom with hydrazine affords the desired hydralazine 319 (Scheme 43).^350–352^

**Scheme 44:**
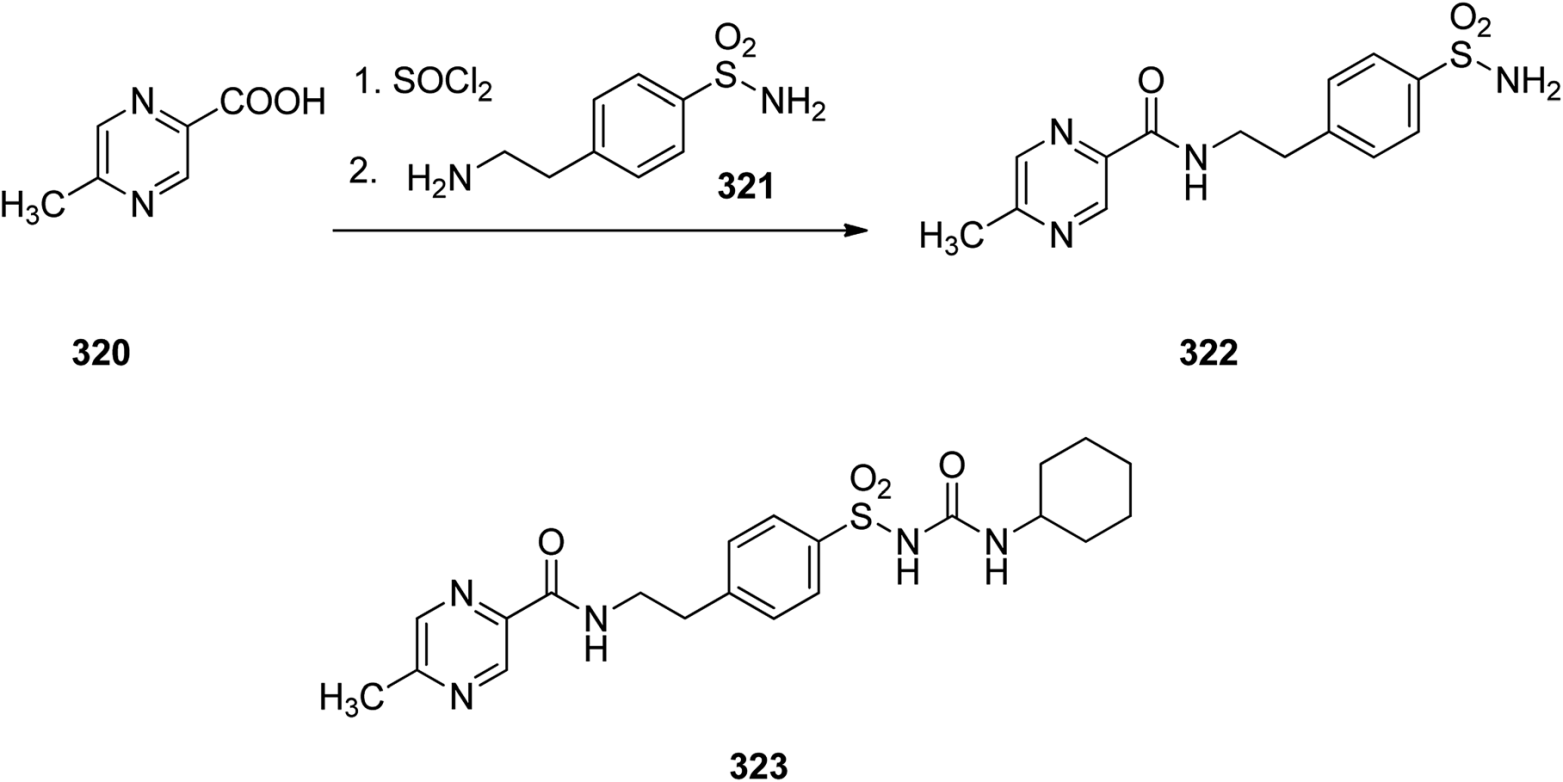
Synthesis of glipizide 323.

Glipizide 323, is an anti-diabetic medication of the sulfonylurea class prescribed for the treatment of type 2 diabetes and is used combined with a diabetic diet. It was approved for medical in 1984 and introduced to market under the brand name Glucotrol.^353^ Glipizide 323 is actually 1-cyclohexyl-3-[[*p*-[2-(5-methylpyrazincarboxamido)ethyl]phenyl]sulfonyl]urea 323. As depicted in Scheme 44, the synthesis of glipizide 323, is started with 6-methylpyrazincarboxylic acid 320 which is initially treated with SOCl_2_, leading to the respective chloride that is further reacted with 4-(2-aminoethyl)benzenesulfonamide 321, giving the expected amide 322. The resulting sulfonamide 322 upon reaction with cyclohexylisocyanate *via* conventional procedure resulted in formation of the desired glipizide 323.^354–356^

**Scheme 45:**
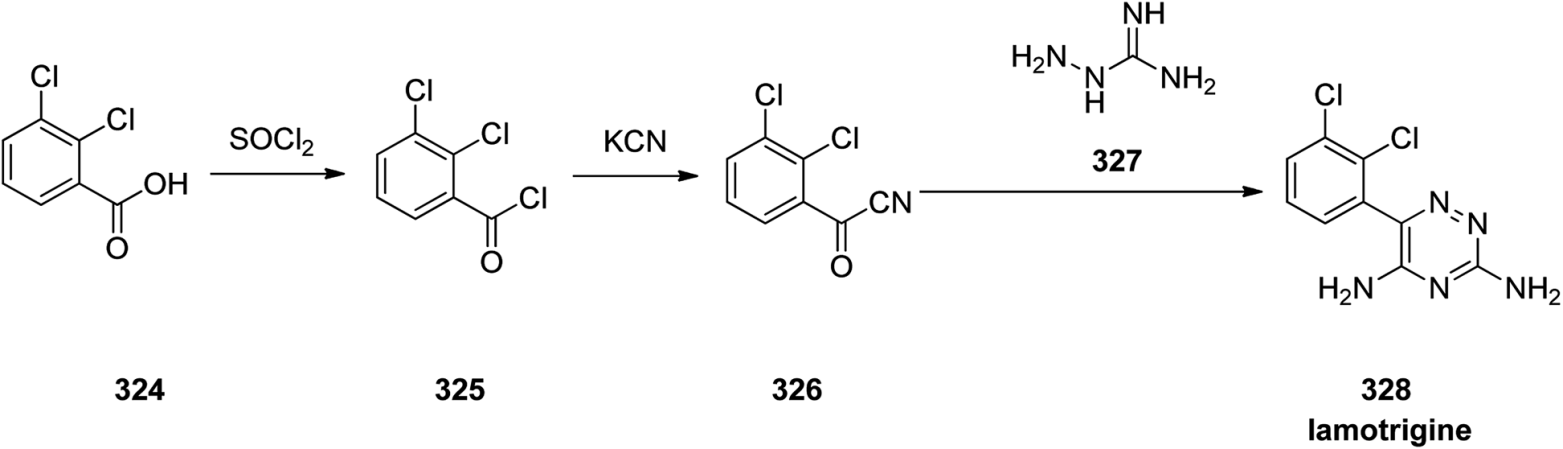
Synthesis of lamotrigine 328.

Lamotrigine 328, came to market in the Great Britain in 1991 as is an anticonvulsant medication prescibed for treatment and epilepsy and bipolar disorder. However, it was approved for being prescribed in the US in 1994.^357^ Nowaday, it is sole under trade name of Lamictal. There are two practical approaches for the formation of lamotrigine 328. The first approach,^358,359^ is relied on condensation of 2,3-dichlorobenzoyl cyanide 326 that is in turn provided by transformation of 2,3-dichlorobenzoic acid 324 to its acid chloride 325 by treatment with tionyl chloride. The latter was then reacted with copper cyanide to provide 2,3-dichlorobenzoyl cyanide 326. Condensation of the latter with aminoguanidine 327, proceeds smoothly to give the desired target lamotrigine 328 in about 16% yield (Scheme 45).^360^

**Scheme 46:**
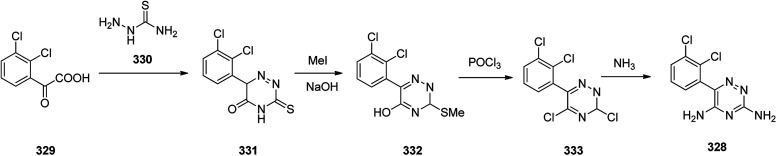
Synthesis of lamotrigine 328.

The alternative approach employed to provide lamotrigine 328 (ref. 361) involved, the reaction of 2-(2,3-dichlorophenyl)-2-oxoacetic acid 329 with thiosemicarbazide 330 to provide 3-thioxo-3,4-dihydro-1,2,4-triazin-5(6*H*)-one derivative 331 which subsequently methylated using MeI in aqueous NaOH provided the corresponding *S*-methylated product 332. The latter upon treatment with especially with phosphorous oxychloride, among other chlorinating agent, gave the *S*-methylated product 332 which was subjected to replacement of its thiomethyl and hydroxyl groups for chlorine, providing compound 333, which, upon treatment with NH_3_, gave the desired target lamotrigine 328 (Scheme 46). Some related methods for the formation of lamotrigines have been underlined in the two useful reviews.^362,363^

**Scheme 47:**
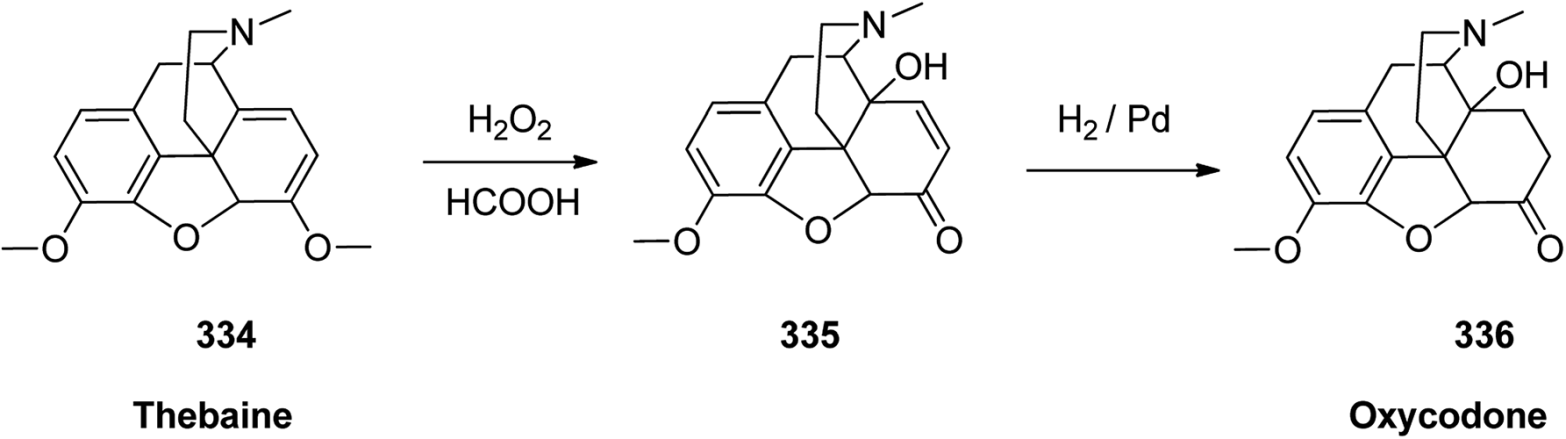
Synthesis of oxycodone 336.

Oxycodone 336, sold under the brand name OxyContin among others, is an opioid medicine used for handle and alleviating of moderate to severe pain.^364^ Oxycodone was first semi-synthesized in Germany in 1916 from a natural product, thebaine, has been a common drug of abuse.^365^ Although the structure of oxycodone is similar to natourios morphine, it has shown better oral bioavailability, making it superior for pain alleviating in some clinical trials.^366^ Oxycodone 336 is prepared from thebaine (paramorphine) 334, that also known as codeine methyl enol ether which is an opiate alkaloid. Thebaine 334, is first converted into intermediate 14-hydroxycodeinone 335 upon oxidation using H_2_O_2_ in formic acid. Upon the selective hydrogenation of the double bond, in 335 the desired target oxycodone 336 was prepared (Scheme 47).^367^

**Scheme 48:**
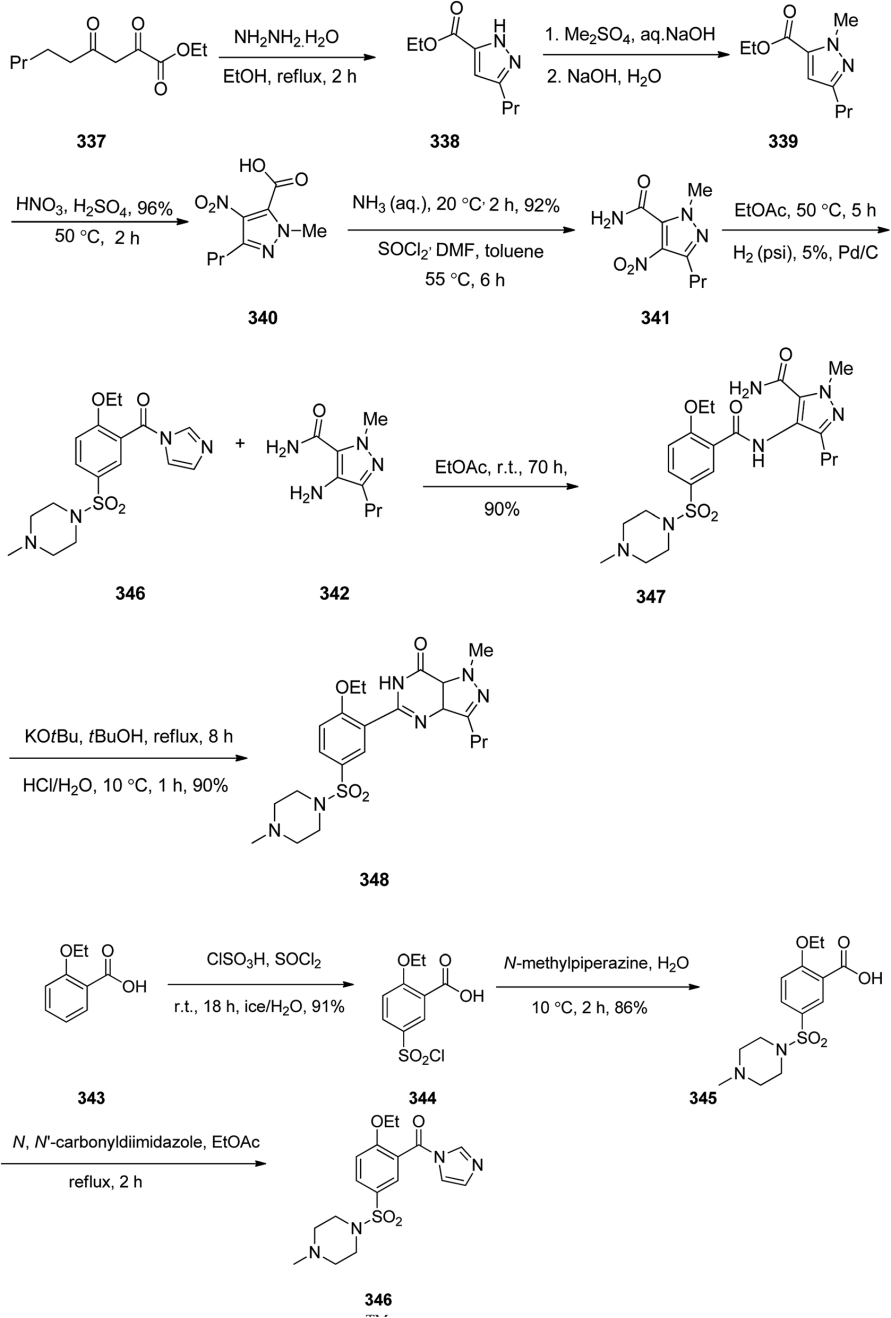
Synthesis of Viagra™ (Sildenafil) in accordance of Phizer procedure.

Sildenafil 348, has been used for the treatment of erectile dysfunction and pulmonary arterial hypertension. As a matter of fact, it was accidentally discovered by Pfizer in 1989 while the researchers were looking for a medication to treat heart-related chest pain.^368^ It was approved for being prescribed in the US and Europe in 1998.^368^ Sildenafil 348 is sold with the brand of Viagra by Pfizer. Sildenafil was initially examined against hypertension with little success but showed promising effects in the male sexual dysfunction.^369^ The synthetic pathway to sildenafil was achieved by the Pfizer research group.^370^ Sildenafil is prepared *via* the reaction of β-diketones with hydrazines. For the synthesis of sildenafil 346, diketone 337 was reacted with hydrazine hydrate in EtOH under reflux. The reaction proceeds through the formation of hydrazone A,^371^ which upon subsequent cyclization and dehydration provides the corresponding pyrazole 338. The latter upon treatment with Me_2_SO_4_, as source of a methyl nucleophile under basic condition (aq. NaOH) gave the corresponding pyrazole 339. The latter was dissolved in conc·H_2_SO_4_, next the fuming nitric acid was mixed with conc. H_2_SO_4_ and added to the pyrazole 339 which concurrently nitrated the pyrazole ring and hydrolysis of the ester moiety to carboxylic acid to give pyrazole 340. The latter was converted to 341 upon treatment with liq. NH_3_ in DMF. Then, the nitro group in 341 was reduced *via* hydrogenation in the presence of Pd as catalyst in ethyl acetate to afford the corresponding pyrazole 342. Sildenafil 348 was synthesized starting from 2-ethoxybenzoic acid 343 in molten form that was added gradually to a mixture of chlorosulfonic acid and thionyl chloride while the reaction temperature was kept below 25 °C. In this way a direct electrophilic aromatic substitution occurred in which the ethoxy group gave a common direction to the electrophile towards the expected *ortho* and *para* position. It was noticed that the addition of thionyl chloride for transformation of the intermediate sulfonic acid into the sulfonyl chloride is essential. In this stage, the reaction was quenched by addition of ice water in which 5-(chlorosulfonyl)-2-ethoxybenzoic acid 344 was precipitated out from reaction mixture. Next, the latter was reacted with *N*-methylpiperazine in water to give 2-ethoxy-5-(4-methylpiperazin-1-ylsulfonyl) benzoic acid 345. The carboxyl group of 345 was activated by a common and effective activating reagent, *N*,*N*′-carbonyldiimidazole (CDI) make it susceptible for nucleaphilic substitution.^372^ Thus the reaction of 345 with *N*,*N*′-carbonyldiimidazole (CDI) in refluxing acetic acid provided (2-ethoxy-5-(4-methylpiperazin-1-ylsulfonyl)phenyl)(1*H*-imidazol-1-yl)methanone 346. The latter was reacted with 342 in ethyl acetate at room temperature to give the desired amide 347 through the usual addition elimination mechanism. In the last step, the primary amide is deprotonated by potassium *tert*-butoxide making it more nucleophilic. This nitrogen as a nucleophile attack the other amide carbon closing the ring. Upon isomerization resulting in the formation of pyrimidone ring the synthesis of sildenafil 348 was completed. Worthy to mention, that the last step includes only water soluble solvents and reagents were used and the final product precipitates out of the aqueous solution upon reaching pH 7.5 to give sildenafil 348 with clinical quality from the filtration in which further purification is non-required (Scheme 48).^373^

**Scheme 49:**
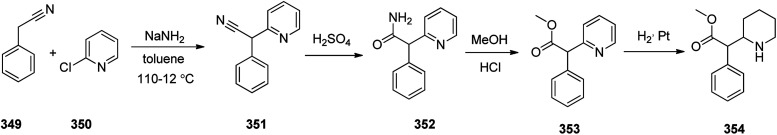
Synthesis of methylphenidate 354.

Methylphenidate 354, is a stimulating medicine used for treatment of attention deficit hyperactivity disorder (ADHD). It is sustaining attention, increases intellectual capacity, and enhance memory.^374,375^ Methylphenidate 354 initially synthesized and patented in 1944. It was first made in 1944 by CIBA and was approved for being prescribed in US in 1955.^376^ It is nowadays sold under the brand name Ritaline by Novartis Corporation.^376^ Methylphenidate 354 can be produced at large scale through reaction of phenylacetonitrile 349 with a 2-chloropyridin 350 at 110–112 °C in toluene in the presence of NaNH_2_ (sodium amide) that afforded 2-phenyl-2-(pyridine-2-yl)acetonitrile 351. The latter was then upon hydrolysis to the respective amide 352 that subsequently treated with hot hydrochloric acid in MeOH afforded methyl 2-phenyl-2-(pyridine-2-yl)acetate 353. The pyridine ring of the latter was hydrogenated to a piperidine ring in HAOc on the platinum or platinum oxide (PtO_2_) as catalyst afforded the desired target methylphenidate 354 (Scheme 49).^377–379^

Loratadine 362, was found to treat several kinds of allergies including allergic rhinitis and hives. It was patented in 1980 and commercialized under trade name Claritin in 1988.^380^ It is also sold in combination with pseudoephedrine, a decongestant, known as loratadine/pseudoephedrine.^381^ As a matter of fact, loratadine, cetirizine and astemizole are second-generation antihistamines that have substituted first-generation antihistamines for example diphenhydramine and ketotifen.^382–388^ Loratadine 362 can be produced *via* various routes. It can be effectively synthesized, based on the formerly reported approaches,^389^ starting from the 8-chloro-5,6-dihydro-11*H*-benzo[5,6]cyclohepta[1,2-*b*]pyridin-11-one ketone 356, which upon treatment with an appropriate Grignard reagent 355 afforded the respective tertiary carbinol that was subsequently dehydrated in acidic media giving the 8-chlorol-1-piperidiylidene derivative 359. The later then was treated with ethylchloroformate under refluxing benzene to afford compound 361 which was transformed to the desired target product, loratadine 362 by refluxing in xylene.^390,391^

Another approach was involved the construction of seven-membered ring scaffold through cyclizing intermediate ketone provided by the reaction of the same Grignard reagent 355 with tailor-made 3-(3-chlorophenethyl) picolinonitrile 357 in a different super acid systems media, preferably, a system comprising HF and BF_3_ resulted in the formation of compound 359 which similarly converted to compound 361 and then by refluxing in xylene was converted to the corresponding its ethyl carbamate which is actually the desired loratadine 362.^392–396^

A third strategy is relied on the use of low-valent titanium catalyzed reductive coupling between the two ketones which are available in hands. They were 8-chloro-5,6-dihydro-11*H*-benzo[5,6] cyclohepta[1,2-*b*]pyridin-11-one 356 and ethyl 4-oxopiperidine-1-carboxylate 358 which upon low-valent titanium assisted reductive coupling gave 8-chloroazatadine 359. The latter was converted to the desired loratadine 362, through above-mentioned two step reaction.^397^

**Scheme 50:**
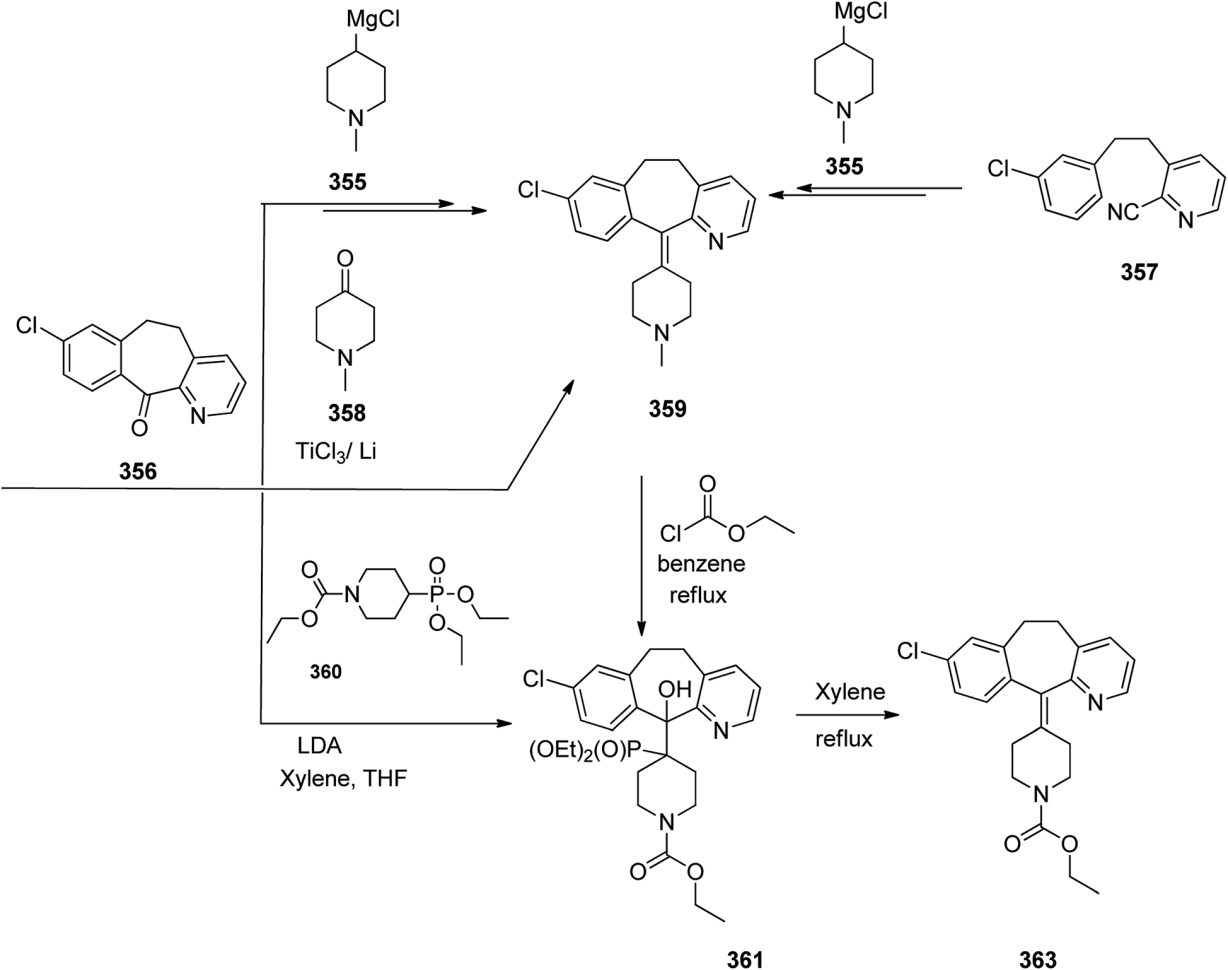
Synthesis of loratadine 362.

Another approach employed the Wittig reaction in which initially ethyl 4-(diethox-ypho sphoryl)piperidine-1-carboxylate 360 was reacted with ketone 356 in presence of lithium diisopropyl amide in xylene-THF media to afford the β-hydroxyphosphonate 361, that was converted to loratadine 362*via* thermal decomposition by being further refluxed in xylene 362 (Scheme 50).^398^

**Scheme 51:**
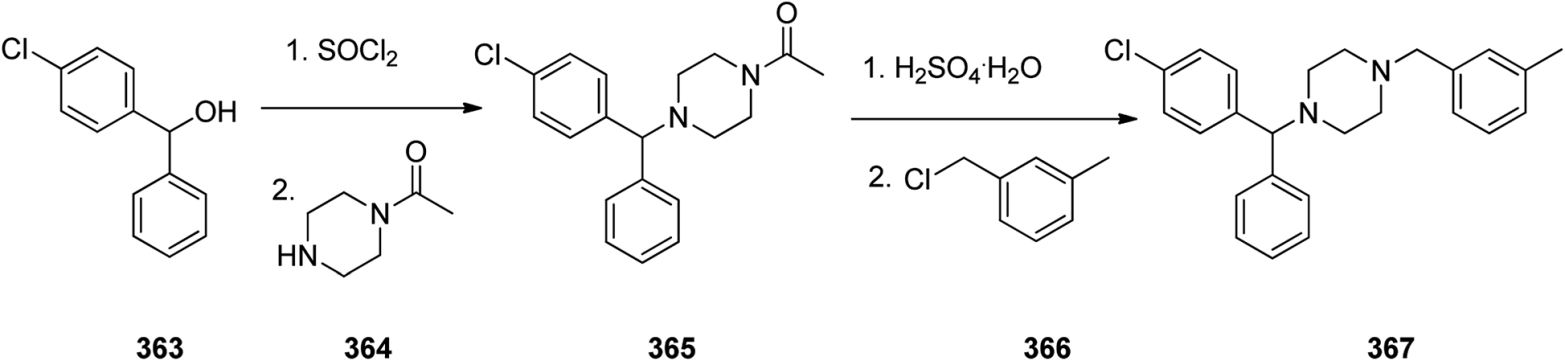
Synthesis of meclizine 367.

Meclizine 367, is also an antihistamine medication employed for treatment of motion disease and the sense like the world is rotating. Meclizine 367 was patented in 1951 and approved for being used as medication under the trade name Bonine in 1953.^399^ (4-Chlorphenyl)-phenylmethanol is halogenated with SOCl_2_ before adding acetylpiperazine. The acetyl group is cleaved with diluted sulfuric acid. An *N*-alkylation of the piperazine ring with 3-methylbenzylchloride completes the synthesis of meclizine 367 (Scheme 51).^400^

**Scheme 52:**
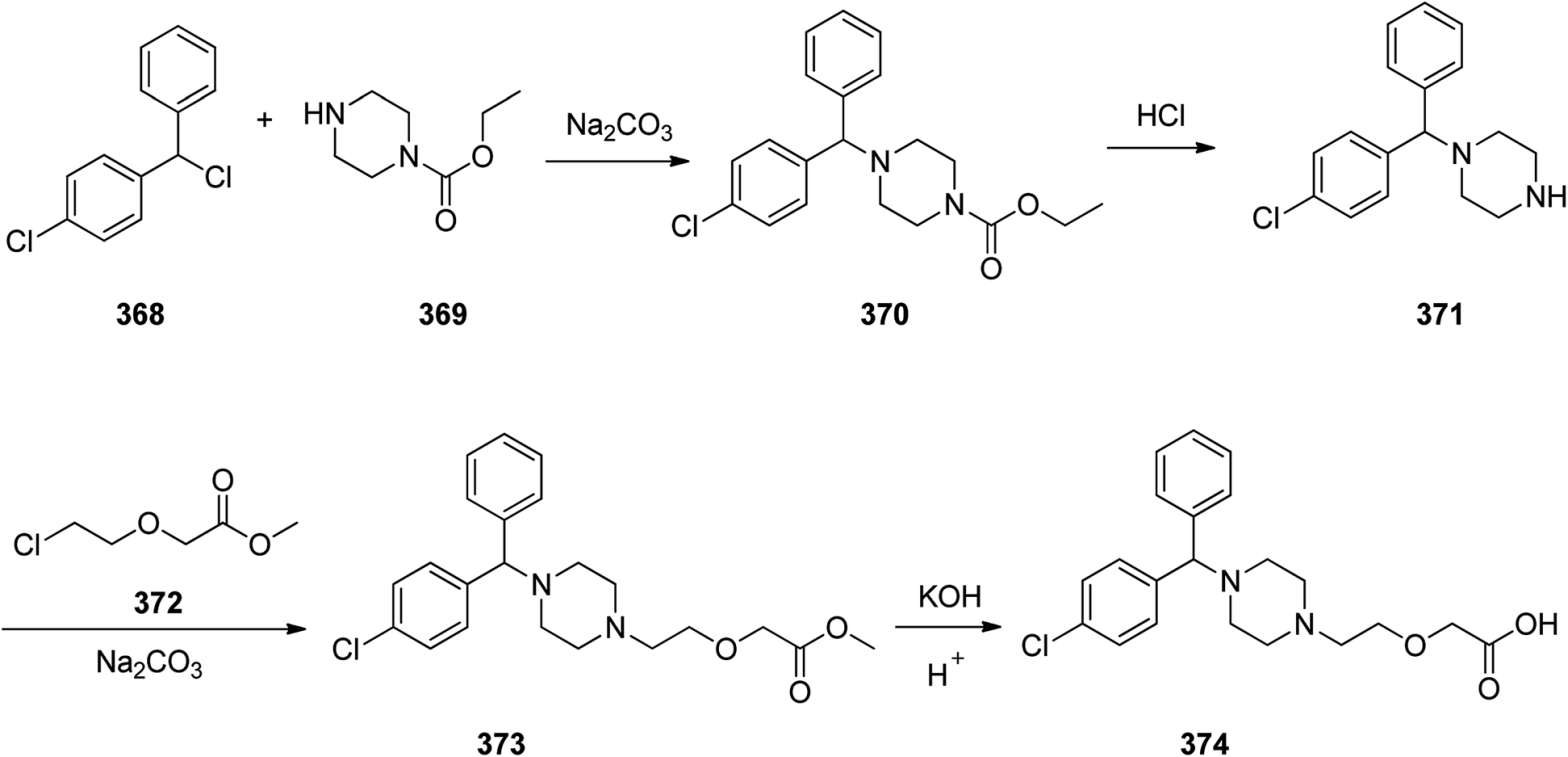
Synthesis of cetirizine 374.

Cetirizine 374, is a second-generation antihistamine used for treatment for allergic rhinitis, dermatitis, and urticarial.^401^ It was patented in 1981 but marketed in 1987 (ref. 402) under the trade name of Zyrtec, (Zyrtec®1, Zirtec®). The pharmacological and medical properties and therapeutic efficiency of cetirizine have already been reviewed.^403–407^ Cetirizine 374 is prepared as a racemic mixture^408,409^ and its isomers can be separated.^409^ The first synthesis of cetirizine 374 commenced from 4-chlorobenzhydrylchloride 378, which was reacted with ethyl piperazine-1-carboxylate 369, in the presence sodium carbonate to provide compound 370. The latter was subjected to acidic hydrolysis (using HCl) to provide the benzhydrylpiperazine derivative 371. Next, the latter was treated with methyl 2-(2-chloroethoxy) acetate in the presence of Na_2_CO_3_ to provide the product 373. The resultant ester was readily subjected to basic hydrolysis to afford the corresponding carboxylic acid as racemate which in fact was the desired target, cetirizine 374 (Scheme 52).^410^

**Scheme 53:**
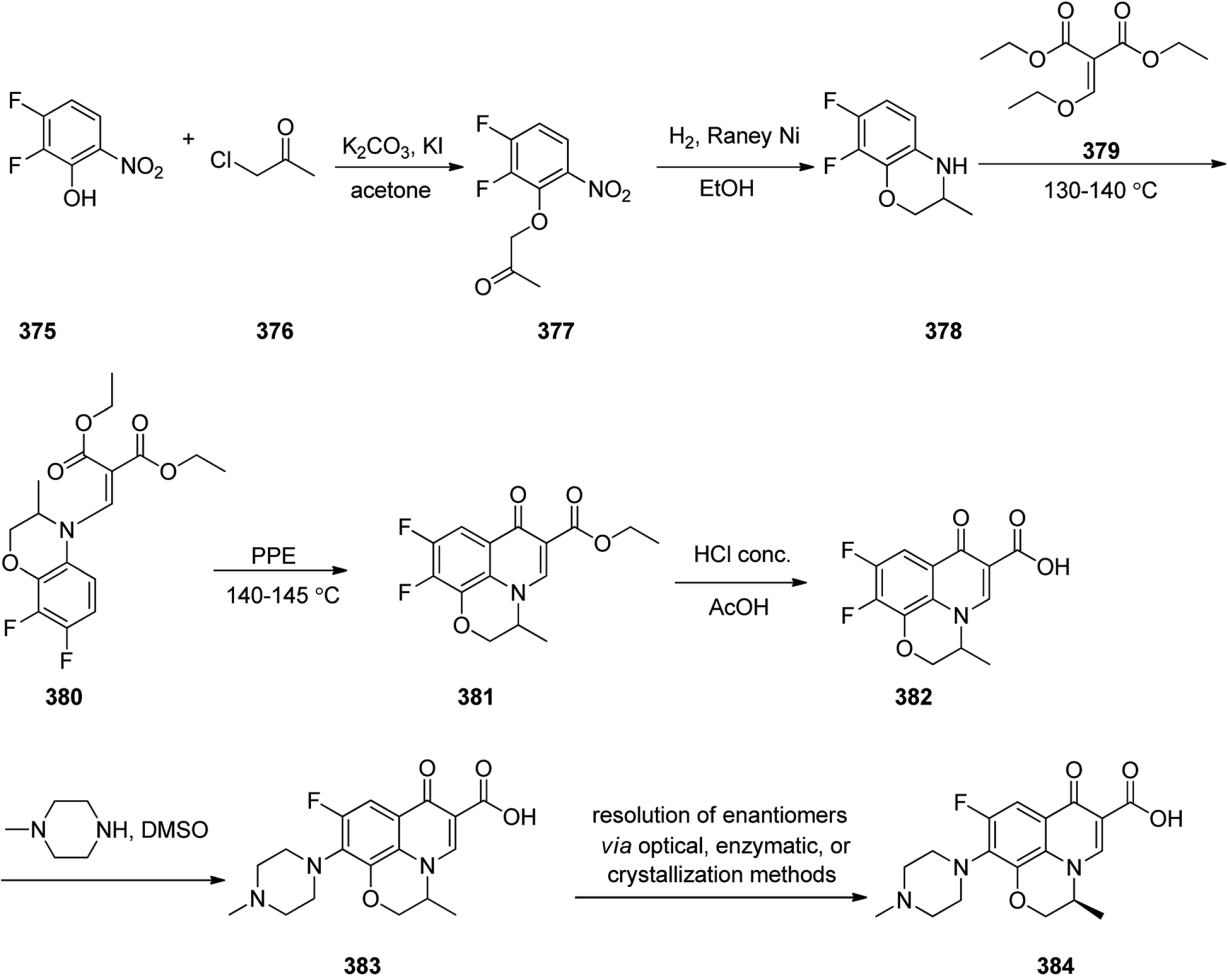
Synthesis of levofloxacin 384.

Levofloxacin 384, is an optically active antibiotic used to treat a number of bacterial infections involving acute bacterial sinusitis. Notably, it is approved for being used in the treatment of community-acquired pneumonia, H. pylori.^411–420^ Levofloxacin was first patented in 1985 and approved for being prescribed in 1996 under the brand name of Levaquin.^421^ Levofloxacin 384 is derived from the typical quinolones which have a more complex fused ring to the oxazinoquinoline core. (−)-Levofloxacin was found being twice as more active than ofloxacin.^422^ The synthetic pathway including resolution of racemic mixture to obtain (−)-levofloxacin 384 was designed and performed as depicted in Scheme 52. Based on this approach, the synthesis began from the reaction of 2,3-difluoro-6-nitrophenol 375 with chloroacetone 376 in the presence of K_2_CO_3_ and potassium iodide to provide 1-(2,3-difluoro-6-nitrophenoxy)propan-2-one 377. Notably, 2,3-difluoro-6-nitrophenol 375 was synthesized from 2,3,4-trifluoro-1-nitrobenzen by replacement of the *ortho* to the nitro group fluorine atom to the hydroxyl group through the reaction with potassium hydroxide in dimethyl sulfoxide. The resultant product 377 was hydrogenated using RANEY® in EtOH to afford a cyclic product, 7,8-difluoro-3-methyl-3,4-dihydro-2*H*-benzo[*b*][1,4]oxazine 378. The latter was reacted with diethyl ethoxymethylenemalonate 379*via* the well-established Gould–Jacobs reaction at 130–140 °C to provide the expected benzoxazinyl methylenemalonate 380 that upon treatment with polyphosphoric ester at 140 to 145 °C provided 9,10-difluoro-3-methyl-7-oxo-2,3-dihydro-7*H*-[1,4]oxazino[2,3,4-*ij*]quinoline-6-carboxylic acid ethyl ester 381. Upon hydrolysis of the latter in HAOc/conc·HCl benzoxazine-6-carboxylic acid was obtained 382. The resultant product 382 was reacted with *N*-methylpiperazine in DMSO resulted in displacement of a secondary amine smoothly, introduces an amino substituent at the C7 position selectively due to the activation by the C4 carbonyl group, giving ofloxacin 383 as racemate. Resolution of racemic mixture of 383 through optical, enzymatic, or crystallization approaches gave (−)-ofloxacin 384 (Scheme 53).^423^

**Scheme 54:**
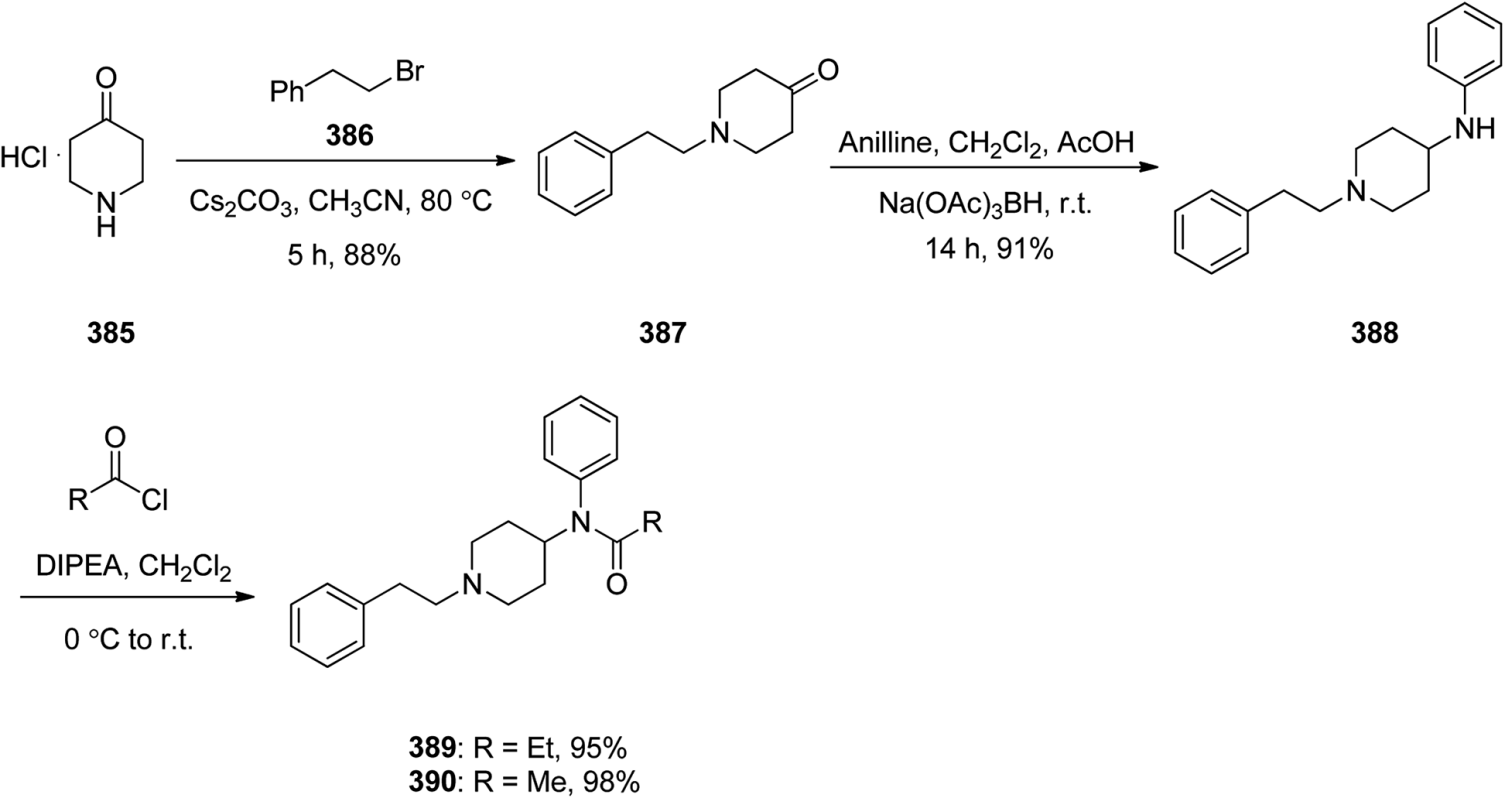
Synthesis of fentanyl 389 and acetylthiofentanyl 390.

Fentanyl 389 is an opioid used as a pain killer and sometimes combined with other medicine for anesthesia. Fentanyl was initially synthesized by Paul Janssen in 1960 and approved for clinical use in the US in 1968. Fentanyl is known more often by its brand name Sublimaze. Their modus operandi is supposed to include the binding to the transmembrane m-opioid receptors on cell surfaces leading to a flow of intracellular signals that finally leads to their biological effect.^424,425^ Several synthetic approaches have been developed for their construction since Janssen's first discovery.^426–429^ The approach is described herein were actually optimized to provide fentanyls in higher yields using a highly effective three-step synthetic method. The multistep synthesis of fentanyl 389, as illustrated in Scheme 54 was started with the alkylation reaction of commercially accessible 4-piperidone monohydrate hydrochloride 385 using 2-(bromoethyl)-benzene in the presence of cesium carbonate in acetonitrile at 80 °C to provide alkylated piperidone 387 in 88% yield. The latter upon reductive amination with aniline in the presence of sodium triacetoxyborohydride in HAOc provided the 4-piperidineamine 388 in excellent yield (91%) as appropriate precursor. Finally, the latter was acylated by propionyl chloride using Hunig's base to produce fentanyl 389 in 95% yield. Similarly, piperidineamine 388 was reacted with acetic anhydride using Hunig's base to produce acetylfentanyl 390 in 98% yield. Transformation of 389 and 390 were easily performed to give their corresponding hydrochloride and citrate salts in almost quantitative yields (Scheme 54).^430^

**Scheme 55:**
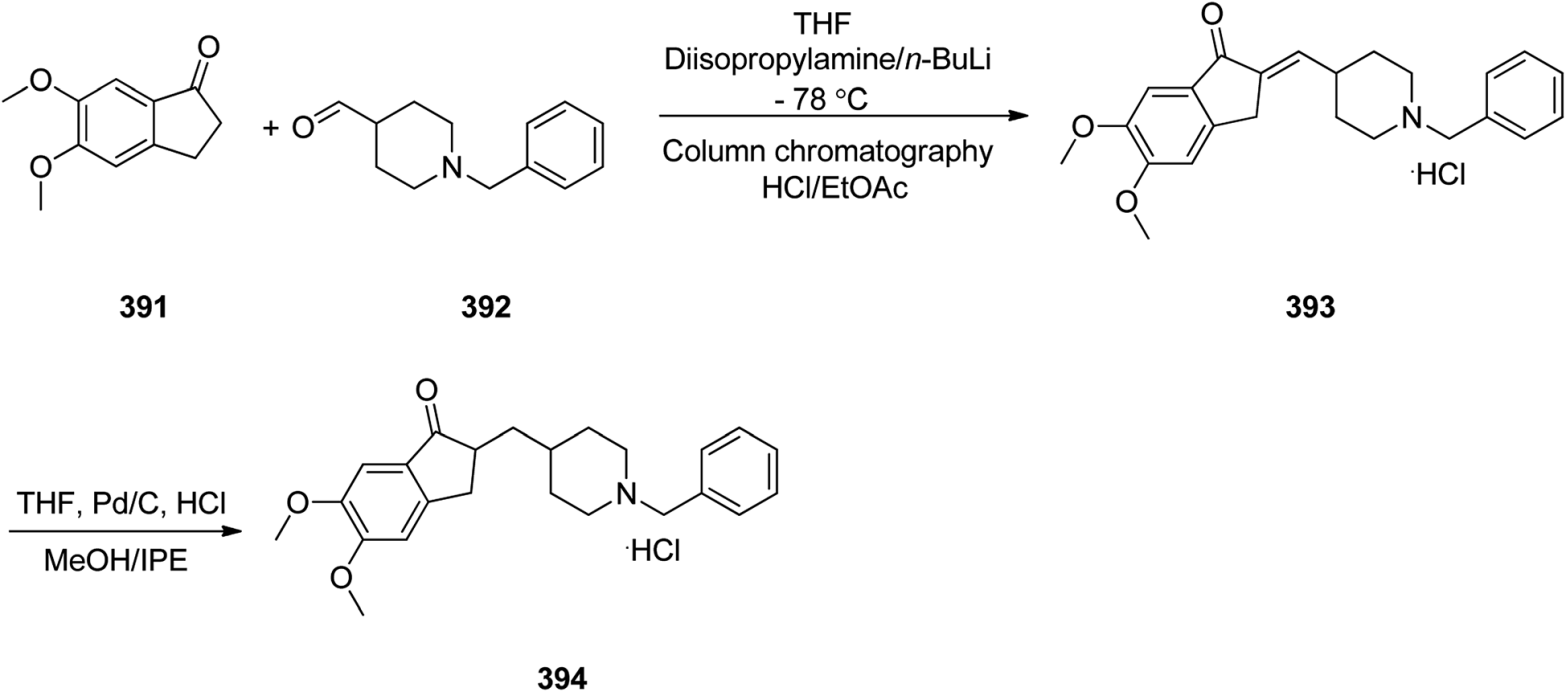
Synthesis of donepezil hydrochloride 394 (Method A).

Donepezil 394, is a medicine prescribed usually to treat Alzheimer's disease. It was confirmed by FDA for being prescribed in 1996 to alleviate to what happens in Alzheimer's disease. Donepezil 394, shows a minor assistance in mental function and ability to function,^431^ but does exhibit any important change in the progression of the disease^432,433^ Donepezil is the most effective inhibitor of the AChE currently accessible on the market, also sold, under the brand name Aricept.^434,435^ Donepezil has been synthesized *via* two different pathways.^436–438^ The synthesis of donepezil was achieved and reported by Sugimoto and co-workers which is a convergent method leading to the production of donepezil hydrochloride 394. It includes aldol condensation of *N*-benzyl piperidine carboxaldehyde 392, with 5,6-dimethoxy indanone 391, under inert atmosphere and in the presence of *n*-butyl lithium and diisopropylamine in hexamethyl phosphoric amide (HMPA) at −78 °C, to produce olefinic compound 393. The double bond of 393 was catalytically reduced in the presence of 10% Pd/C in THF provided, donepezil as free base that was further transformed into its hydrochloride salt 394 upon treatment with HCl/MeOH and isopropyl ether 27% overall yield (Scheme 55, Method A).^439^

**Scheme 56:**
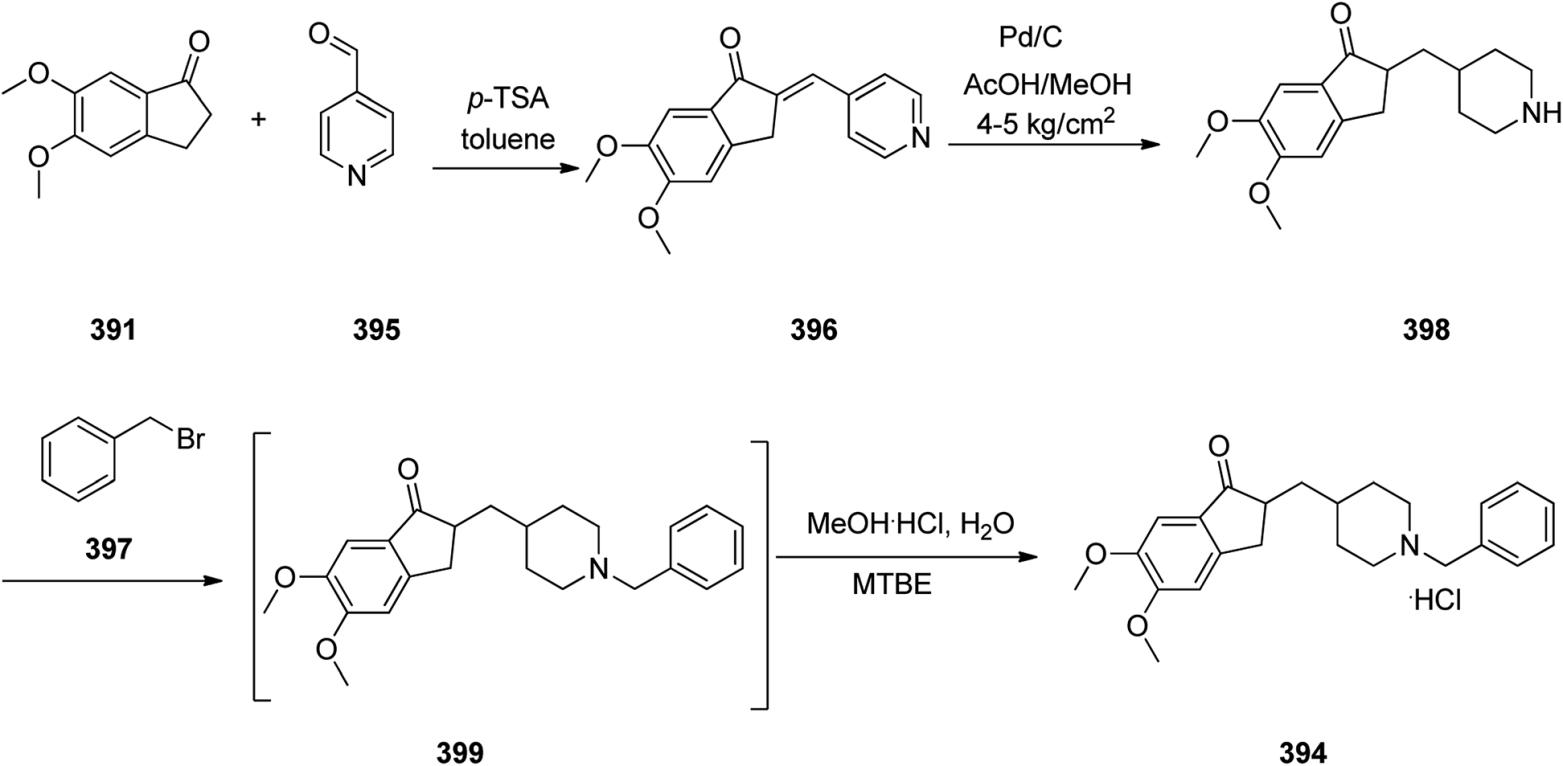
Synthesis of donepezil hydrochloride 394 (Method B).

Alternatively, Elati research group accomplished and reported^440–442^ a route for the synthesis of donepezil hydrochloride 394. In this strategy, initially, 5,6-dimethoxy-1-indanone 391 was condensed with pyridine-4-aldehyde 395 to afford olefinic compound, 5,6-dimethoxy-2-(pyridine-4-yl)methyleneindan-1-one 396. The double bond of the latter was reduced in the presence of Pd carbon catalyst using HAOc in MeOH to produce compound 398 which was further alkylated with benzyl bromide 397 to give donepezil as free base 399. The last was then upon further treatment with HCl in mixture of MeOH, H_2_O and methyl *tert*-butyl ether produced donepezil hydrochloride 394 (Scheme 56, Method B).

**Scheme 57:**
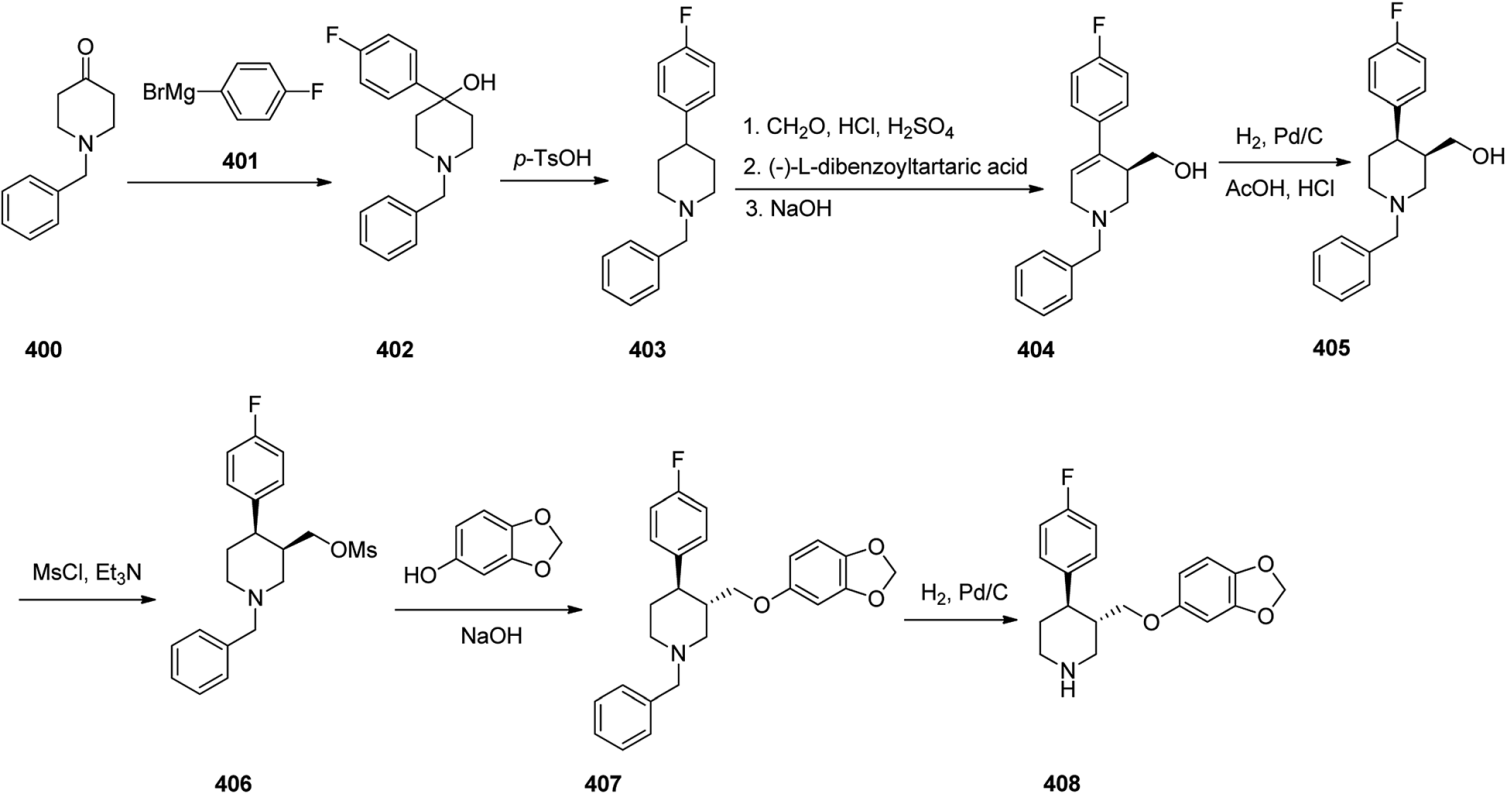
Synthesis of paroxetine 407.

Paroxetine 408, approved for being prescribed in 1992 by FDA, is an antidepressant of the selective serotonin reuptake inhibitor (SSRI) class. It was commercialized under the trade names of Paxil and used for the treatment of social anxiety disorder, major depressive disorder, obsessive, panic disorder, and general anxiety disorder.^443–450^ Several routs for the synthesis of paroxetine were designed and suggested, designated in the recently published review.^451^ One of the rather practical approaches, being used in large-scale production of paroxetine 408 is started with 1-benzyl-4-piperidone 400 which upon the reaction with the Grignard reagent, 4-fluorophenyl magnesium bromide 401 afforded the corresponding tertiary alcohol 402. Upon the treatment of the latter with *p*-toluene sulfonic acid (PTSA), dehydration took place resulting in the formation of the corresponding tetrahydropyridine derivative 403. The latter was subjected to the Prins reaction conditions (using HCOH, HCl, H_2_SO_4_) to give the racemate of tetrahydropyridine-3-methanol that can be resolved using (2)-l-dibenzoyltartaric acid to afford 404. The latter was subjected to the stereoselective reduction over palladium/C catalyst, under acidic conditions in H_2_O resulted in formation of *cis*-(3*R*,4*R*) isomer of piperidine-3-methanol 405, due to retention of *N*-benzyl protective group. The obtained *cis*-alcohol 405 was reacted with methanesulfonyl chloride to give the corresponding *cis*-mesylate 406. The reaction of the latter with sodium sesamolate led to the formation of *trans N*-benzylparoxetine 407 that upon debenzylation upon hydrogenation over palladium/C catalyst afforded the desired target paroxetine 408 (Scheme 57).^451^

**Scheme 58:**
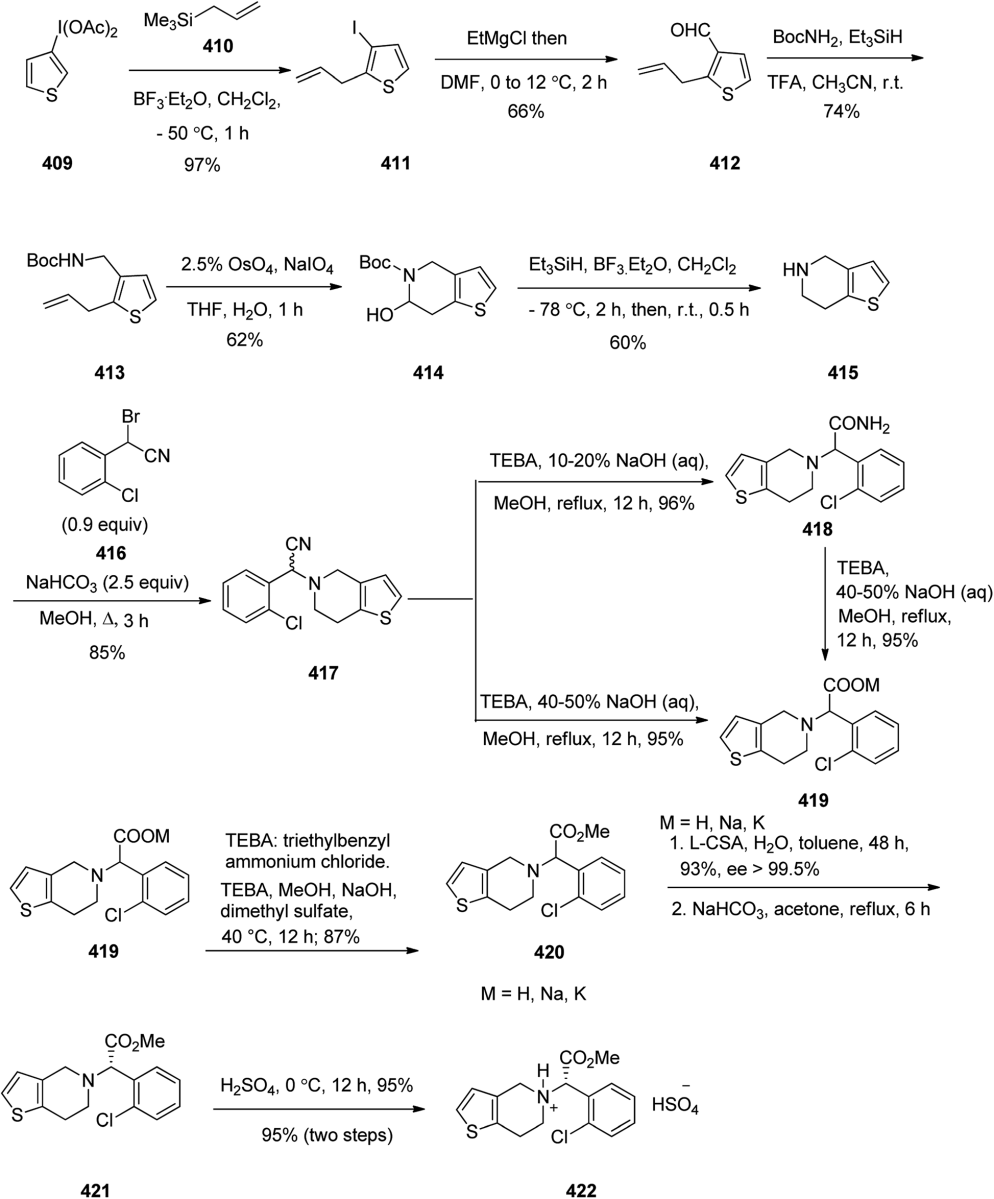
Synthesis of optically pure clopidogrel 422.

Clopidogrel 422, is also an antiplatelet medication prescribed to diminish the risk of heart disease and attacks in those at high risk. Clopidogrel was patented in 1982, and approved by FDA for being prescribed in 1997.^452^ Clopidogrel 422, came to market under the brand name of Plavix. Clopidogrel 422, was synthesized commencing with easily accessible 3-iodothiophene diacetate 409, which upon treatment with allyltrimethylsilane mediated by BF_3_·Et_2_O at −50 °C to give the expected 2-allyl-3-iodothiophene 411 in almost quantitative yield. The latter was then submitted to magnesium–halogen exchange to generate the corresponding heteroaryl magnesium followed by formylation to give aldehyde 412 in good yield. The latter was then underwent reductive amination using *tert*-butyl carbamate mediated by triethylsilane and trifluoroacetic acid to give Boc-protected amine 413 in good yield. Then, the latter was subjected to a dihydroxylation-oxidative cleavage of the terminal olefin in one pot fashion, affording the respective aldehyde which concurrently cyclized to give hemiaminal 414 in satisfactory yield. Ultimately, the last was subjected to reductive amination of hemiaminal 414 mediated triethylsilane and BF_3_·Et_2_O with subsequent *in situ* deprotection of the Boc-carbamate moiety gave the important intermediate 415 in good yield. The latest was then upon treatment with 2-bromo-2-(2-chlorophenyl)acetonitrile 416 mediated by NaHCO_3_ in MeOH gave the vital intermediate 2-(2-chlorophenyl)-2-(6,7-dihydrothieno[3,2-*c*]pyridine-5(4*H*)-yl)acetonitrile 417. Delightfully, the direct alkaline hydrolysis of nitrile 417 to acid 419 was achieved virtually in quantitative yield when it was performed in the presence of phase transfer catalyst and in the mixed solvent and high concentration of inorganic strong base, while by monitoring the concentration of base (<20%), amide 418 can also be created selectively. Apparently, there is no method found in literature concerning the direct alkaline hydrolysis of nitrile 417 to acid 419. Next, metal salt of 419 was reacted with dimethyl sulfate, using TEBA (triethylbenzylammonium) in NaOH/MeOH afforded the desired expected compound 420, which in two steps gave the respective 421. By using 0.45–0.55 equiv. of l-CSA in toluene, a highly selective and efficient kinetic resolution took place giving optically pure clopidogrel with higher than 98.3% ee and 88% chemical yield. Clopidogrel 422 was obtained in even higher optical purity of above 99.5% ee, just by washing it with isopropanol at room temperature (Scheme 58).^453^

**Scheme 59:**
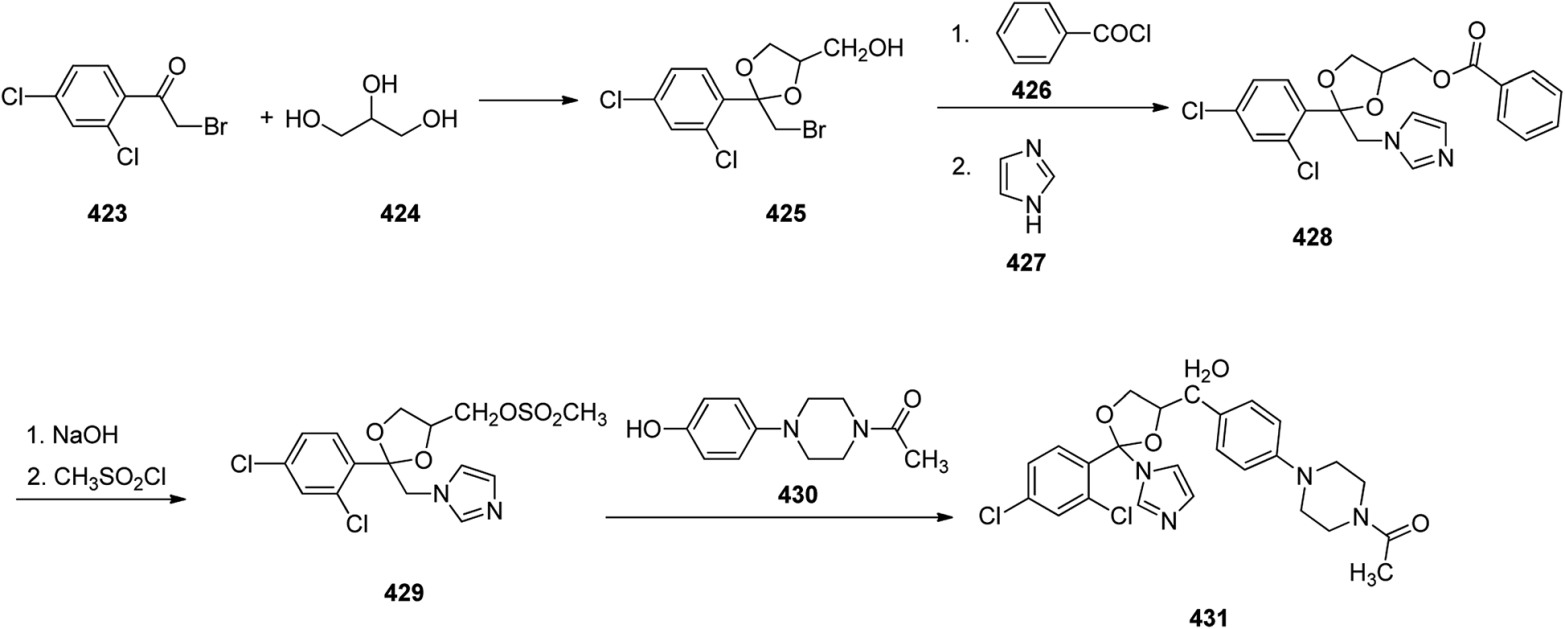
Synthesis of ketoconazole 431.

Ketoconazole 431, was patented in 1977 and approved in 1981 as an antifungal medicine, prescribed commonly for the treatment of several fungal infections. Ketoconazole 431, is mainly used to treat fungal skin infections such as versicolor, dandruff, tinea, seborrheic dermatitis, cutaneous candidiasis and pityriasis.^454^ Ketoconazole 431, is also sold under the trade name Nizoral. Ketoconazole 431, is in fact chemically named, *cis*-1-acetyl-4-[4-[2-(2,4-dichlorophenyl)-2-(1*H*-imidazole-1-ylmethyl)-1,3-dioxolan-4-ylmethyl]phenyl]piperazine 431. It can be synthesized from the reaction of 2,4-dichlorophenacyl bromide 423 with glycerol 424 affording *cis*-2-(2,4-dichlorophenyl)-2-bromoethyl-4-hydroxymethyl-1,3-dioxolane 425. The hydroxyl group of 425 can be benzoylated using benzoyl chloride followed by alkylating the resulting compound using imidazole to furnish, compound 428. Upon the alkaline hydrolysis of the latter which eliminates the benzoyl group and subsequent reaction of the resultant with methanesulfonyl chloride afforded 1-acetyl-4-(4-hydroxyphenyl)piperazine 429. Lastly, upon alkylating the latter with 430 gave ketoconazole 431 in satisfactory yield (Scheme 59).^455–459^

**Scheme 60:**
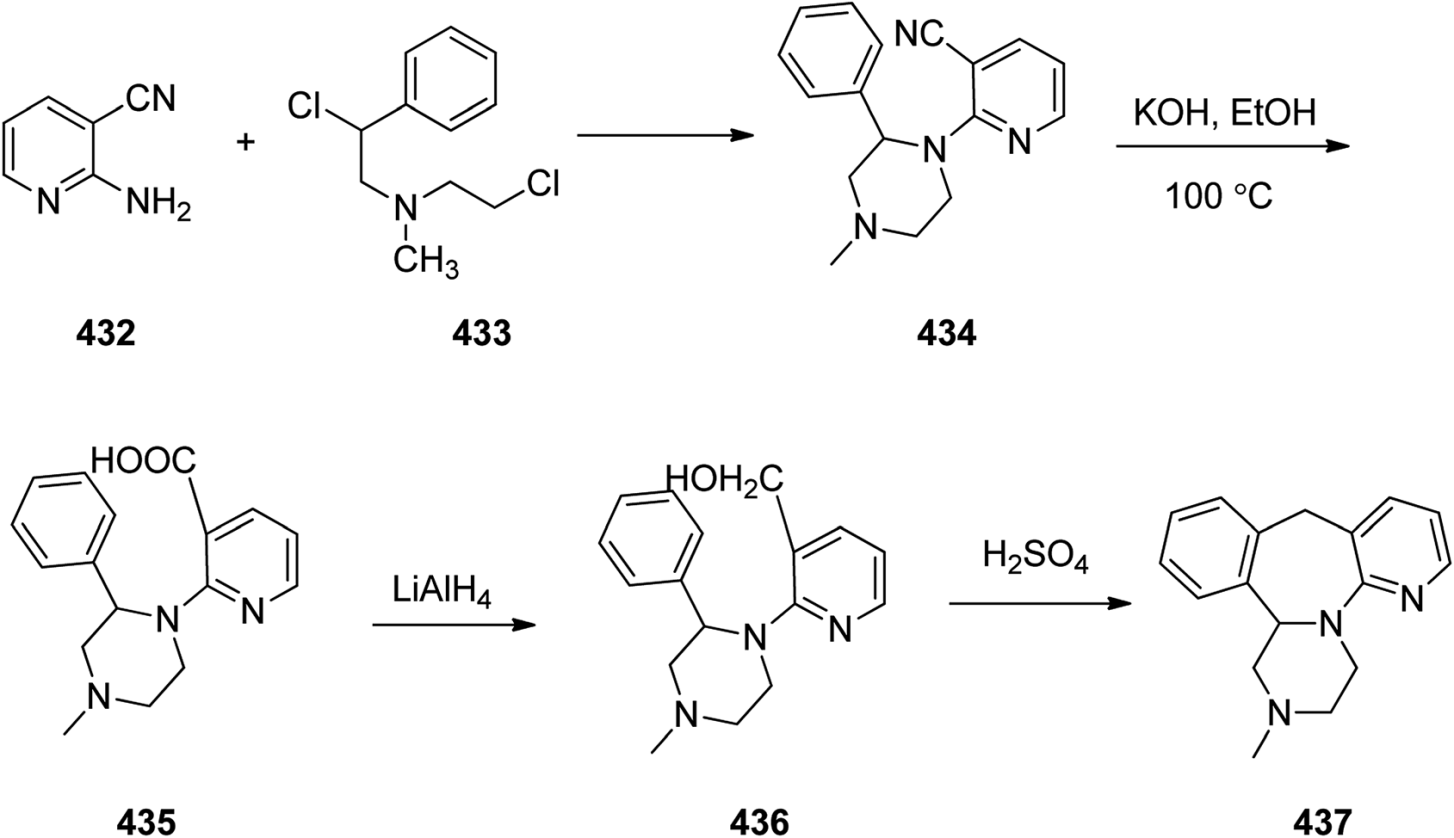
Synthesis of mirtazapine 437.

Mirtazapine 437, was approved by FDA for being prescribed in the United States in 1996 as an antidepressant. It primarily used to treat depression,^460^ depression combined by anxiety or for suffering sleeping.^460,461^ Mirtazapine 437 has a tetracyclic chemical structure distinct to other classes of antidepressants for example, those that are selective serotonin reuptake inhibitors, tricyclics or monoamine oxidase inhibitors.^462,463^ Mirtazapine 437, is in fact chemically known as 2-methyl-1,2,3,4,10,14*b*-hexahydrobenzo[*c*]pyrazino(1,2-*a*)pyrido[3,2-*f*]azepine. Various approaches have been achieved and reported in the literature for the production of mirtazapine.^464–466^ However, still some impurities are traced in the mirtazapine tablets, sold in the market,^467^ van der Burg and co-workers achieved and reported the synthesis of mirtazapine 437 started with 2-amino-3-cyanopyridine 432 which was reacted with *N*-methyl-1-phenyl-2,2′-iminodiethyl-chloride 433 to afford 1-(3-cyanopyridyl-2)-4-methyl-2-phenylpiperazine 434 (cyano-NMPP). The latter upon hydrolysis of its nitrile group under highly basic conditions (KOH/EtOH) at high temperatures (100 °C) for a long time (24 h) provided the 1-(3-carboxypyridyl-2)-4-methyl-2-phenylpiperazine 435. The vital intermediate-1-(3-hydroxy-methylpyridyl-2)-4-methyl-2-phenylpiperazine 436 was provided by reduction of 435 using lithium aluminium hydrate (LiAlH_4_) as an efficient reductive agent. Compound 436, upon treatment H_2_SO_4_ produced mirtazapine 437 (Scheme 60).^468^

**Scheme 61:**
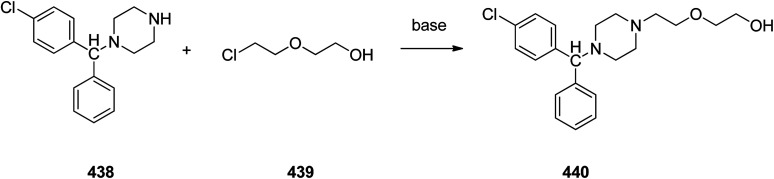
Synthesis of hydroxyzine 440.

Hydroxyzine 440 is selective antihistaminic medication, used in the treatment of itchiness, anxiety, and nausea. It is classified as a first generation antihistamine containing piperazine. Hydroxyzine 440 was first produced in 1956 and was approved for being prescribed in the United States. Nowadays, hydroxyzine 440 has been commercialized and sold under the trade name Atarax.^469^ Hydroxyzine 440 is chemically, 2-[2-[4-(*p*-chloro-α-phenylbenzyl)-1-piperazinyl]-ethoxy] ethanol which is produced by the alkylation reaction between 1-(4-chlorobenzohydril)piperazine 438 and 2-(2-hydroxyotoxy)ethylchloride 439 in the presence of an appropriate base (Scheme 61).^470–475^

**Scheme 62:**
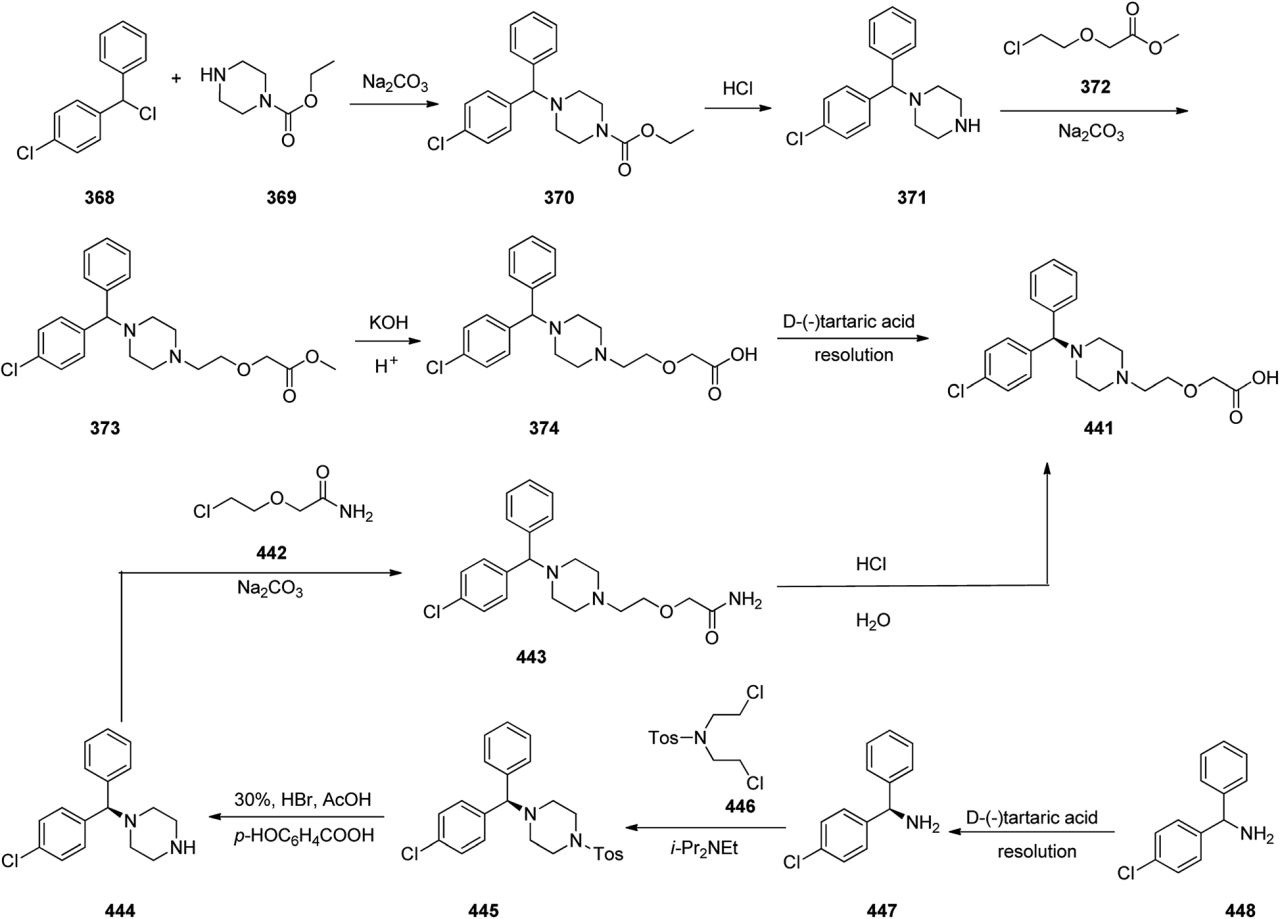
Synthesis of levocetirizine 441.

Levocetirizine 441, approved by FDA in 2007, is an antihistamine prescribed for the treatment of allergic rhinitis.^476^ Levocetirizine 441 (ref. 477–480) signifies a third generation of antihistamines that was patented and approved after the second-generation such as cetirizine. The enantioriched levocetirizine 441 was obtained *via* a conventional resolution of cetirizine as racemate using d-(−)-tartaric acid. The synthesis of cetirizine as racemate^408,409^ commenced with 4-chlorobenzhydrylchloride 368, which, reacted with ethyl piperazine-1-carboxylate 369, to provide compound 370. The last was subjected to acid hydrolysis to provide the benzhydrylpiperazine derivative 371. The latter was then treated with methyl 2-(2-chloroethoxy) acetate in the presence of Na_2_CO_3_ to afford the product 373. The resultant ester was readily transformed into cetirizine in the form of free acid 374 and next the desired target product, levocetirizine 441, was isolated through the classic racemic resolution *via* crystallization of d-(−)-tartaric salt from the racemic mixture.^409^ Alternatively, each stereoisomer of cetirizine was asymmetrically synthesized. One alternate^481^ commenced with from each isomer of 4-chloro-benzhydrylamine 448, which separated with the utilization of (−)-, or (+)-tartaric acids (*R*)-(4-chlorophenyl)(phenyl)methanamine 447. The suitable enantiomer was then upon treatment with *N*,*N*-bis(2-chloroethyl)-4-methylbenzenesulfonamide 446 in diisopropylethylamine under reflux provided a tosyl derivative 445 that easily by crystallization from EtOH was purified. Reductive elimination of the *N*-tosyl group in 445 using 4-hydroxybenzoic acid (phenol component) in HBr/CH_3_COO gave product 443 in highly pure form. Then, upon alkylation of the latter with 2-(2-chloroethoxy)acetamide compound 443 was obtained that was hydrolyzed in acidic media (HCl) to provide the desired target levocetirizine 441 as a single stereoisomer (Scheme 62).

**Scheme 63:**
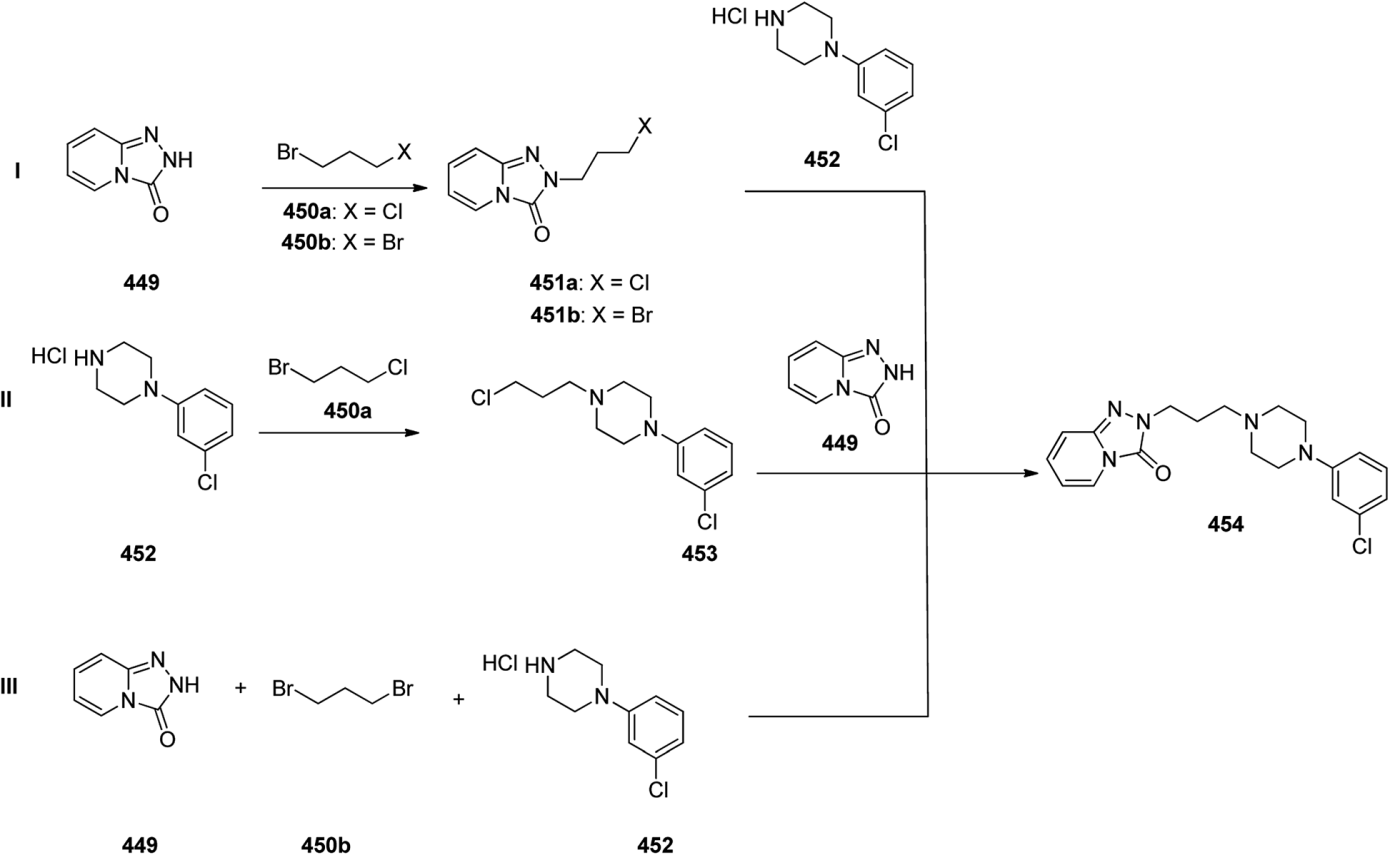
Methods for obtaining trazodone 454.

Trazodone 454, is a medication taken orally to treat main depressive and anxiety disorders, and also used as component with other drugs to treat alcohol dependence. Trazodone 454 was approved by FDA for being prescribed in US in 1981. Trazodone is also marketed as an antidepressant medication^482^ under several brand trade names. Trazodone 454 has been successfully synthesized from the reaction of 1,2,4-triazolo[4,3-*a*]pyridin-3(2*H*)-one 449 with 1-bromo-3-chloropropane 450a or 1,3-dibromopropane 450b to afford, 2-(3-halopropyl)[1,2,4]triazolo[4,3-*a*]pyridin-3(2*H*)-one 451a/b which upon reaction with 1-(3-chlorophenyl) piperazine hydrochloride 452, in the presence of K_2_CO_3_ as a reaction medium, a PTC (phase transfer catalyst) field, under MWI gave the desired compound trazodone 454 (Scheme 63, Method I).

An alternative but similar process also gave rise to the production of trazodone 454. Reaction of 1,2,4-triazolo[4,3-*a*]pyridin-3(2*H*)-one 449 with chloropropyl-2-chloroarylpiperazine 453 gave trazodone 454, directly (Scheme 63, Method II). Moreover, these conditions can be also performed *via* “one-pot” fashion which has its own merit including, that isolation and purification of intermediates is non-required (Scheme 63, Method III).^482^

**Scheme 64:**
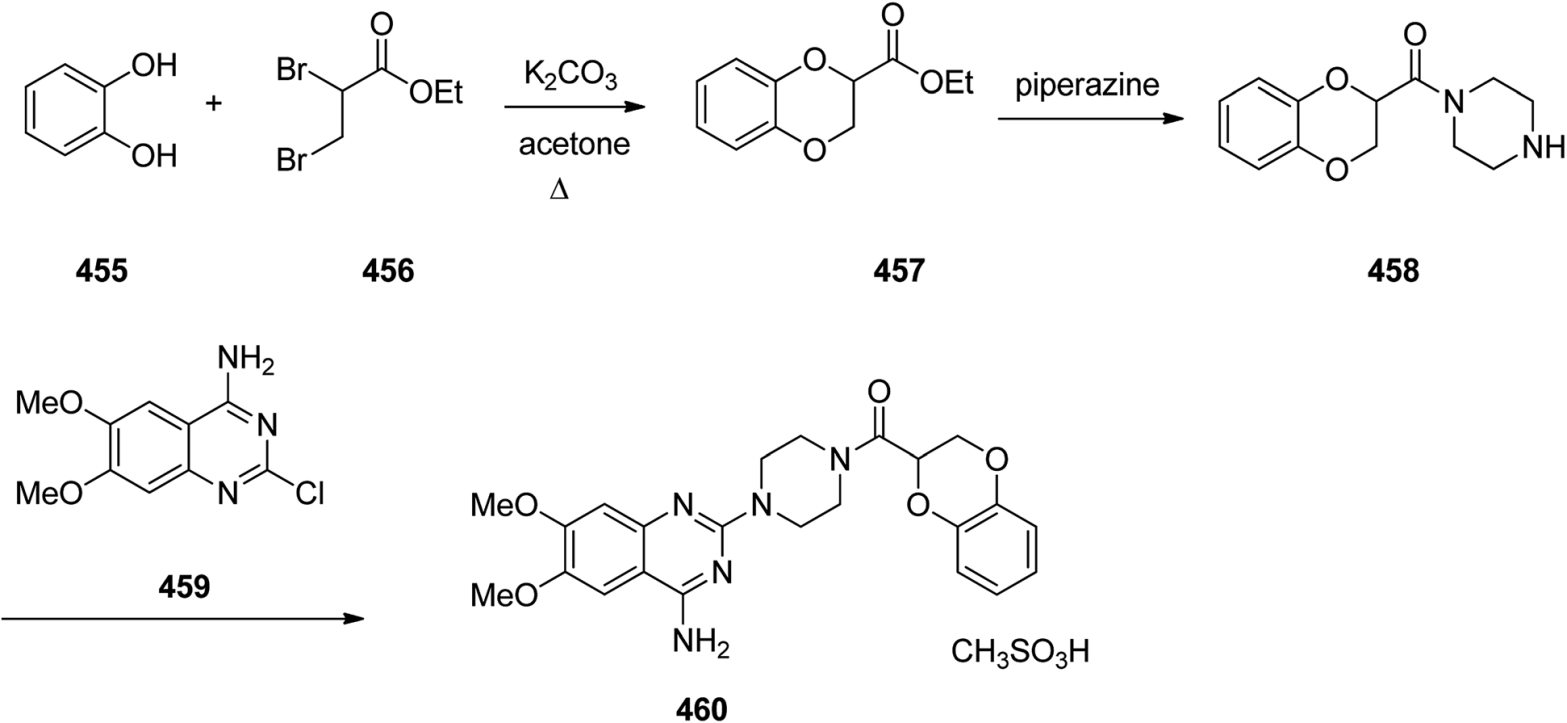
Preparation of doxazosin mesylate 460.

Doxazosin 460, first was patented in 1977 and approved for being prescribed to treat symptoms of an enlarged prostate and high blood pressure in 1988,^483^ and came to market a trade name of Cardura. Doxazosin 460 also showed a positive influence on coronary heart disease by reducing lipids.^484,485^ Doxazosin (doxazosin mesylate) 460 was synthesized in three steps starting from catechol 455 which was treated with 2,3-dibromopropionate in the presence of K_2_CO_3_ in acetone to afford ethyl 2,3-dihydro-1,4-benzodioxin-2-carboxylate 457. The latter was further refluxed with piperazine to give 1-(2,3-dihydro-1,4-benzodioxine-2-carbonyl) piperazine 458. The last was then condensed with 4-amino-2-chloro-6,7-dimethoxyquinazoline 459 to attain doxazosin mesylate 460. As depicted in Scheme 64, in the three steps synthesis of doxazosin mesylate 460 apparently five compounds are involved which are the stating materials and precursors, however, worthy to notice that a side product, a bis-amide (impurity-V), which is generated during the second step will be present as impurities in doxazosin mesylate 460.^486^

**Scheme 65:**
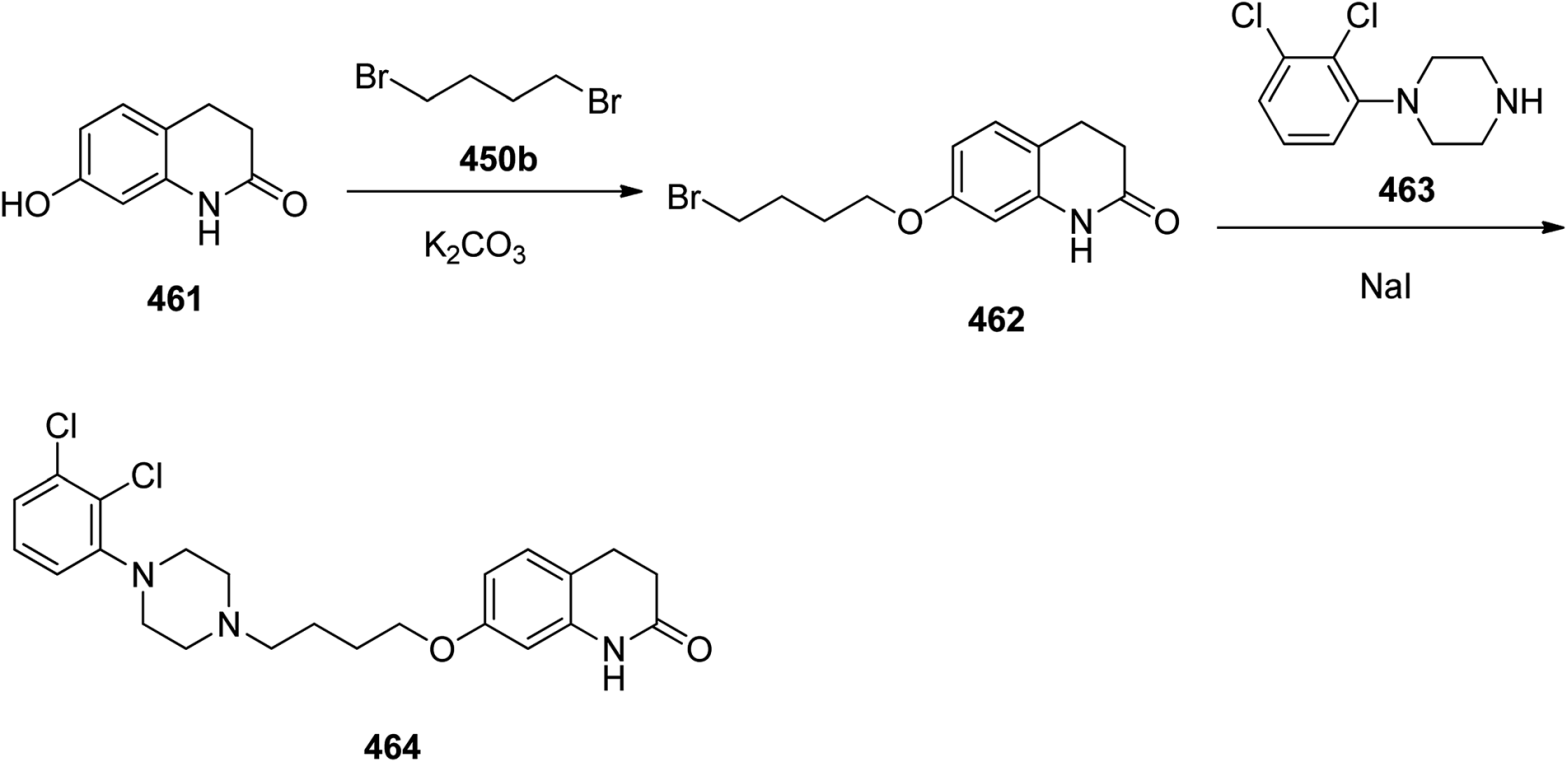
Synthesis of aripiprazole 464.

Aripiprazole, sold under the brand name Abilify among others, is an atypical antipsychotic. Aripiprazole 464 actually is an achiral quinolinone derivative, 7-[4-[4-(2,3-dichlorophenyl)piperazin-1-yl]butoxy]-3,4-dihydro-1*H*-quinolin-2-one.^487^ These physicochemical assets fulfill the Lipinski's rule of five and give it with bioavailability, such as protein binding, and an acceptable metabolic profile.^488^ The first synthetic route and reporting its antipsychotic activity was revealed by Oshiro and co-workers in 1991,^489^ in 1998, Otsuka researchers designated a similar synthetic route for its free base, but under somewhat different conditions (Scheme 65).^490,491^ The two step synthesis started with the alkylation reaction of 7-hydroxy-3,4-dihydro-2(1*H*)-quinolinone 461 by reaction with 1,4-dibromobutane 450b using K_2_CO_3_ in DMF at 60 °C to afford 7-(4-bromobutoxy)-3,4-dihydro-2(1*H*)-quinolinone 462. The latter then treated with sodium iodide in MeCN under reflux and 1-(2,3-dichlorophenyl)piperazine,^492^ and Et_3_N were added to the reaction mixture, and refluxed in the same vessel to give the free base of aripiprazole as a white powder. This powdery substance can be dissolved in EtOH, treated with different acids to give the various corresponding salt. Other compounds, for example OPC-4392, aripiprazole's precursor, were synthesized following similar procedures by taking (4-bromobutoxy)-2(1*H*)-quinolinone (or a structural analog) and reacted with different phenylpiperizines.^489,490^ It should be mentioned that this protocol has since been optimized.^492^ Aripiprazole can also be produced by different simpler approaches.^493–496^

**Scheme 66:**
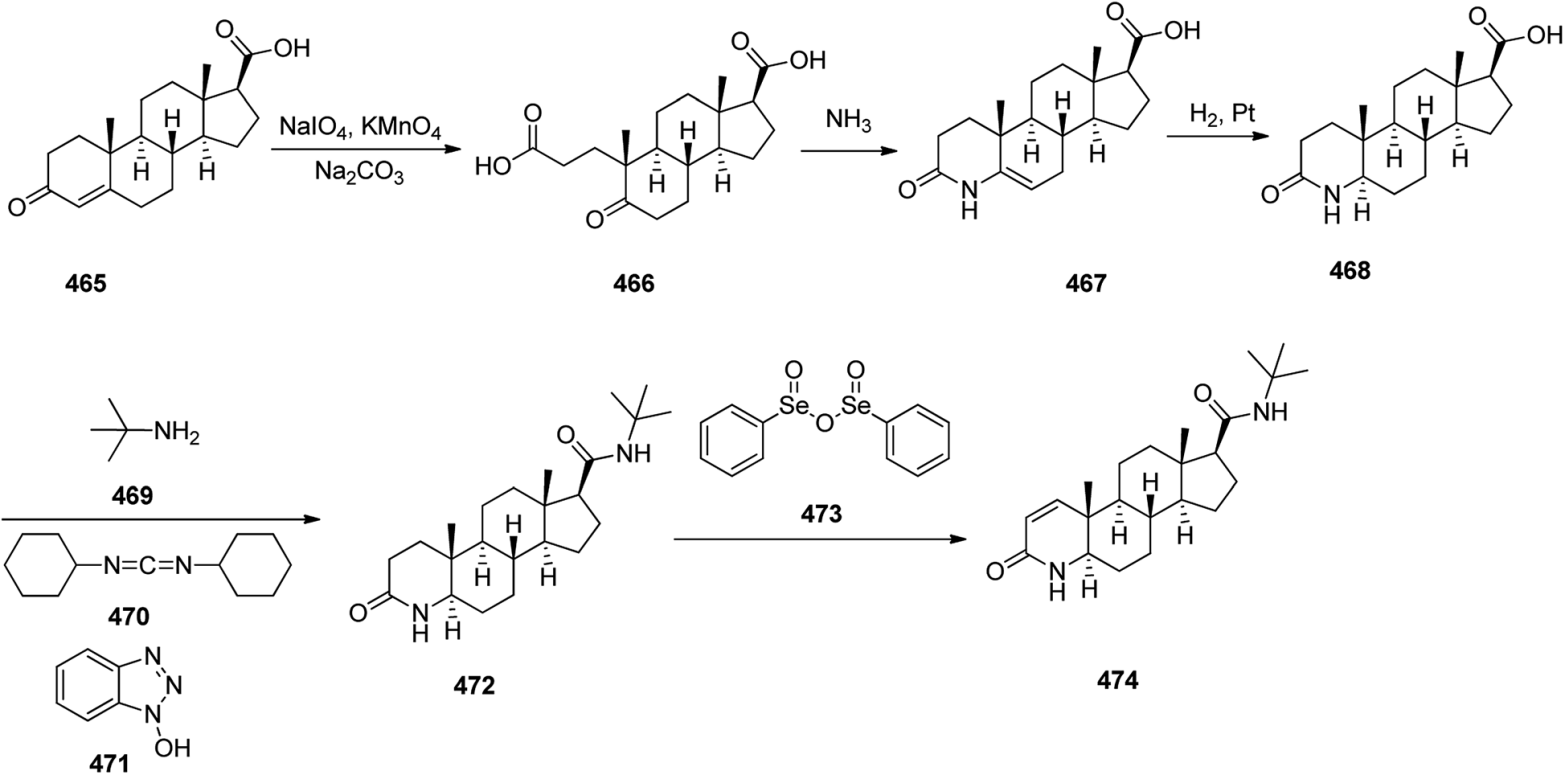
Synthesis of finasteride 474.

Finasteride 474, was patented in 1984 and approved in 1992 for being used as medicine to treat an enlarged prostate or hair loss in men.^497–499^ Finasteride came to market under the brand name Proscar. The synthesis of finasteride was patented and then published along with the chemistry of 4-azasteroids.^500,501^ Finasteride 474 was synthesized *via* multi steps pathway as depicted in Scheme 66. The synthesis commenced with 3-oxo-4-androstene-17β-carboxylic acid 465 which upon oxidative cleavage in a mixture of *t*-butyl alcohol and aqueous sodium carbonate with sodium periodate and potassium permanganate provided the corresponding diacid 466. The latter was subjected to ring closure with *t*-butyl amine in cold ethylene glycol and liquid NH_3_ and the solution was then gradually heated to 180 °C, in the same vessel generating the intermediate 3-oxo-4-aza-5-androstene-17β-carboxylic acid 467. The last was hydrogenated over Pt as catalyst to provide 4-azasteroid 468. The resultant acid was mixed with dicyclohexylcarbodiimide and *N*-hydroxybenzotriazole in dichloromethane and *t*-butyl amine to provide saturated azasteroid 472 that upon oxidation with benzeneseleninic anhydride in chlorobenzene provided the desired target finasteride 474 (Scheme 66).^502^

**Scheme 67:**
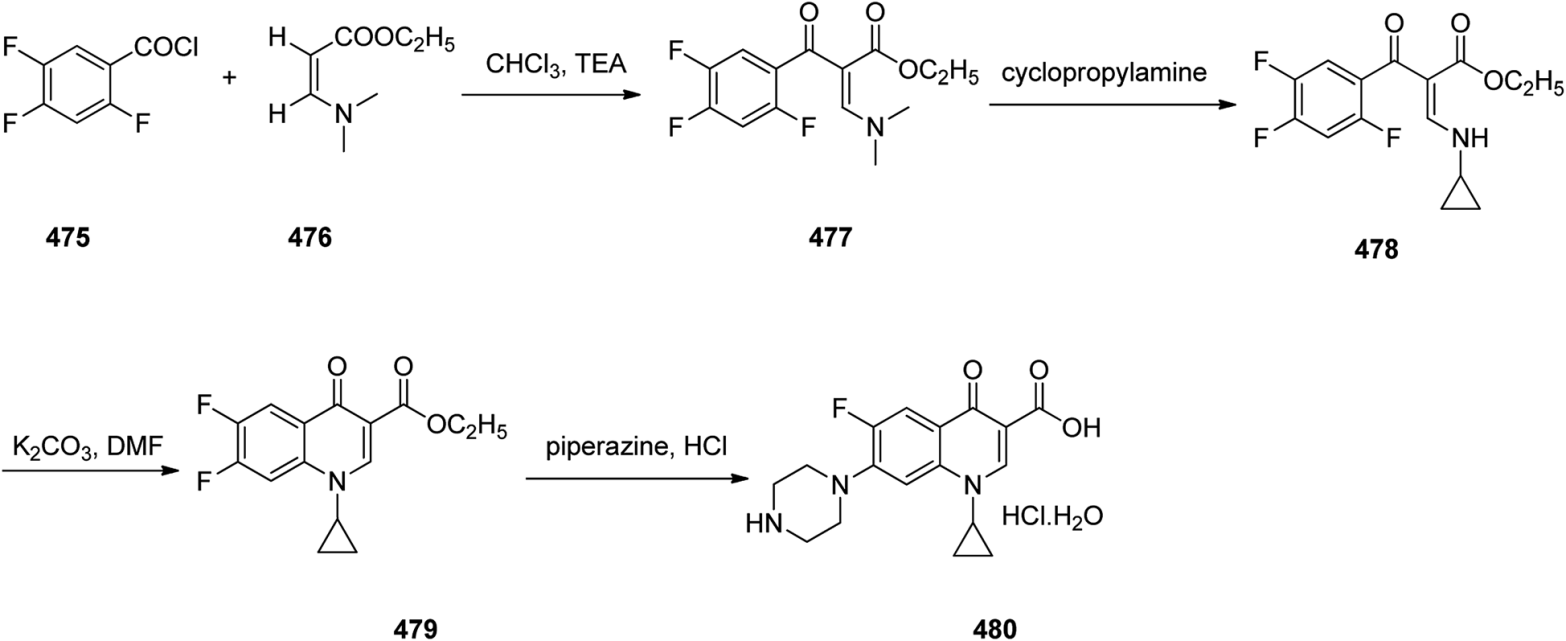
Synthetic process of ciprofloxacin 480.

Ciprofloxacin 480 is a multipurpose antibiotic used for the treatment of a wide range of bacterial infections. It is extensively used to treat austere infections of the urinary, respiratory, and gastrointestinal tracts.^503^ It was patented in 1980 and approved in 1987.^504^ Ciprofloxacin is chemically (1-cyclopropyl-6-fluoro-1,4-dihydro-4-oxo-7-(1-piperazinyl)-3-quinolinecarboxylic acid) 480, the interesting feature of ciprofloxacin is to contain a quinolone ring bearing fluorine atom at the C-6 position of it bicyclic rings.^505^ Ciprofloxacin 480 was originally synthesized by Klaus Grohe (worked for Bayer)^506^ thus is also sold under the brand name of ciprofloxacin Byer. Its synthesis started with the reaction of 2,4,5-trifluoro benzoyl chloride 475 and amino acrylate 476 in the presence of TEA in chloroform to afford the corresponding condensed product 477. The latter was then reacted with cyclopropylamine to afford compound 478 which is subsequently cyclized in the presence of K_2_CO_3_ in DMSO to afford the corresponding fluroquinolinone 479. At last the latter was reacted with piperazine in the presence of HCl to afford ciprofloxacin hydrocgloride 480 (Scheme 67).^507^

**Scheme 68:**
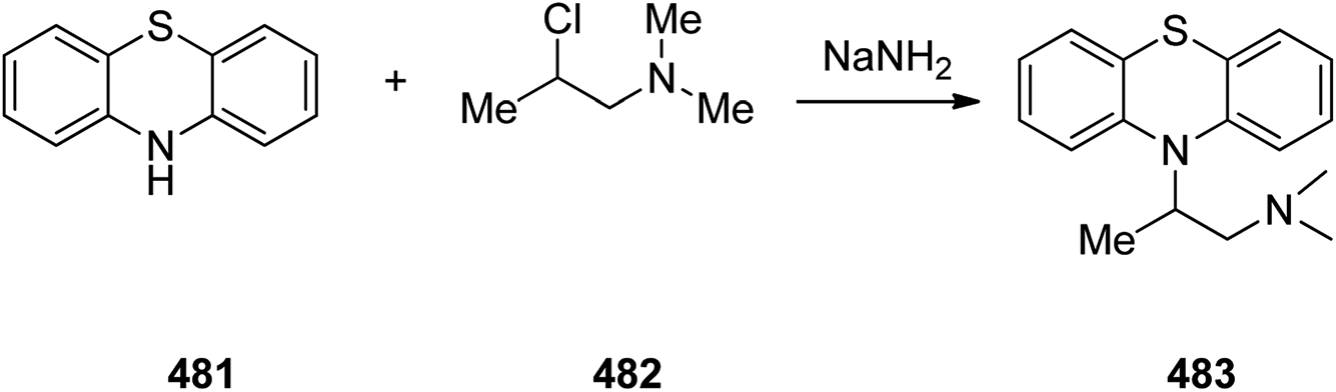
Synthesis of promethazine 483.

Promethazine 483 is categorized in the list of first-generation antihistamine. It is prescribed for the treatment of allergies, sleeplessness, and nausea. Promethazine was first synthesized during 1940s by researchers of Rhône-Poulenc laboratories^508^ and approved for being used as medication in 1951. Promethazine, which chemically is 10-(2-dimethylaminopropyl)phenothiazine 483, was synthesized by alkylating phenothiazine 481 using 1-dimethylamino-2-propylchloride 482 (Scheme 68).^509,510^

**Scheme 69:**
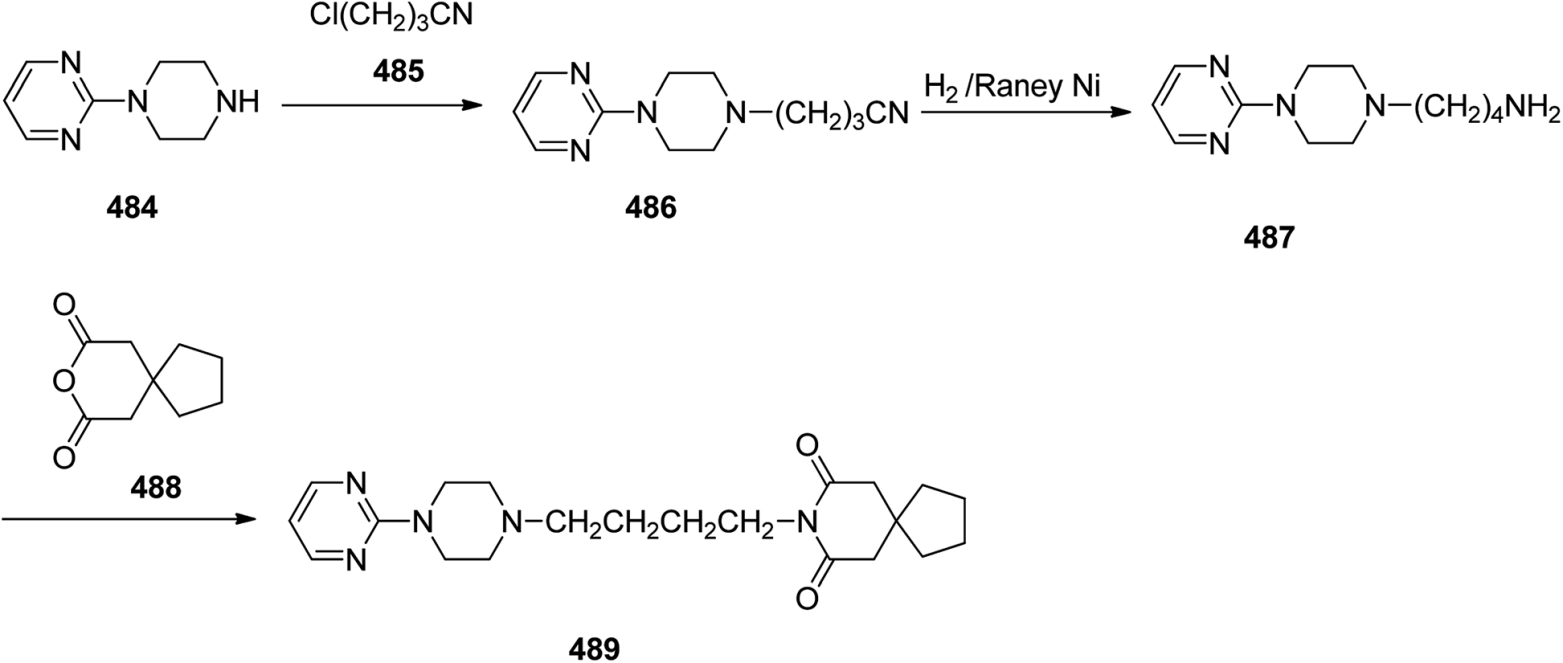
Synthesis of buspirone 489.

A chemical compound, so-called 8-[4-[4-(2-pyrimidyl)-1-piperazinyl]butyl]-8-azaspiro[4,5]decan-7,9-dione was first synthesized in 1968 and after standard clinical trials, approved being used as medication in 1986 to treat anxiety disorders, particularly generalized anxiety disorder. Buspirone 489, was came to market under the trade name Buspar. Buspirone 489 is an extremely specific drug that could possibly represent a new chemical class of anxiolytics-azaspirones but has not been found to be effective in treating psychosis.^255^ Buspirone, 8-[4-[4-(2-pyrimidyl)-1-piperazinyl]butyl]-8-azaspiro[4,5]decan-7,9-dione 489, was synthesized started with 1-(2-pyrimidyl)piperazine 484 which reacted with 4-chlorobutyronitrile 485, to afford 4-(2-pyrimidyl)-1-(3-cyanopropyl)piperazine 486. The latter was hydrogenated in the presence of RANEY® to give, 1-(2-pyrimidyl)-4-(4-aminobutyl)piperazine 487 which upon reaction with 8-oxaspiro[4,5]decan-7,9-dione 488 afforded the desired compound, buspirone 489 (Scheme 69).^255^

**Scheme 70:**
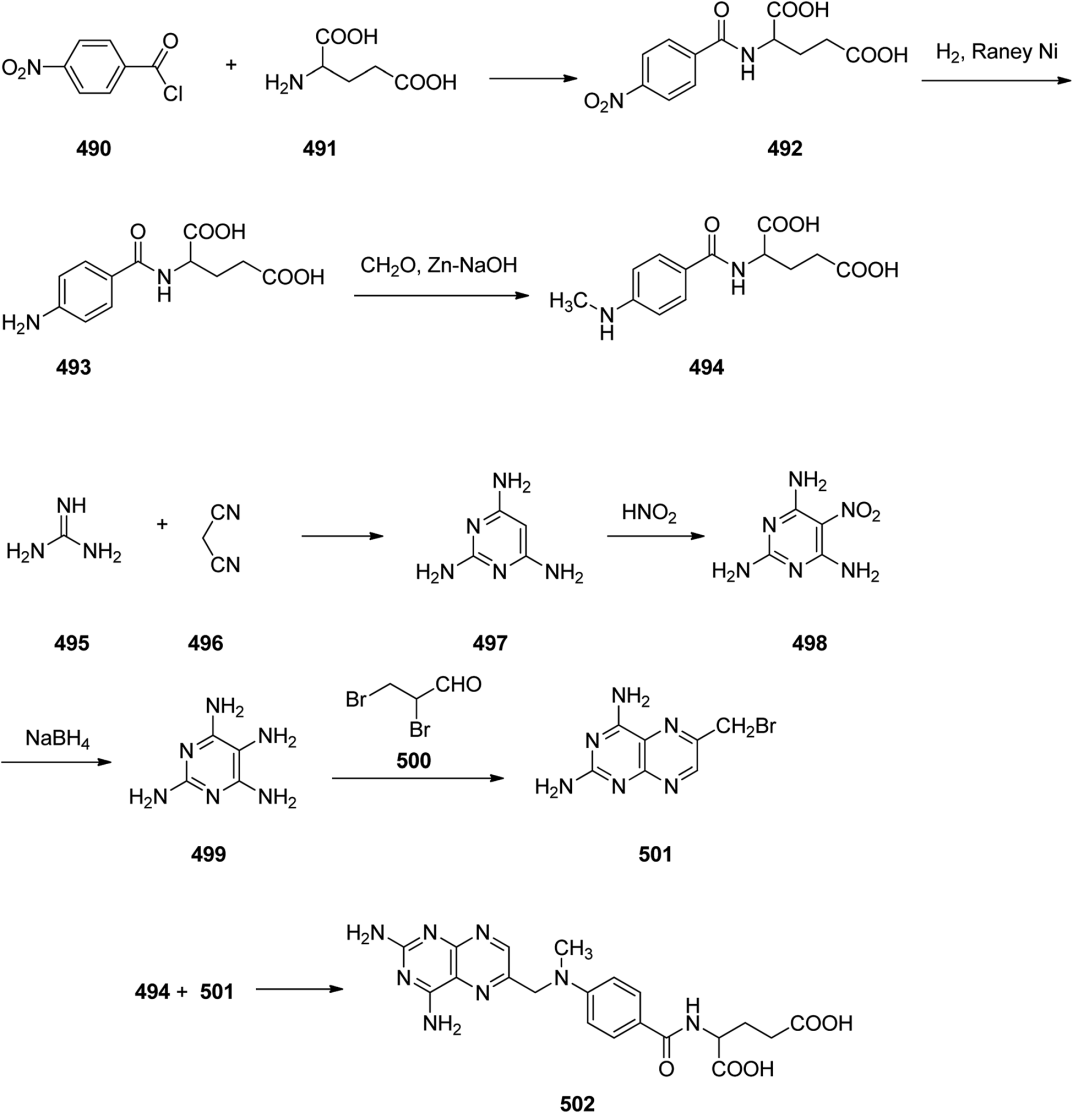
Synthesis of methotrexate 502.

Methotrexate 502, known as amethopterin, is a medication used as a component of chemotherapy and immune system suppressant. Methotrexate was first synthesized in 1947, and initially was used to treat cancer.^511^ Methotrexate 502, chemically is, *N*-[*p*-[[(2,4-diamino-6-piperidinyl)methyl]methylamino]-benzoyl]-l-(±)-glutamic acid 502. It was produced by a pathway as depicted in Scheme 70. Its multistep synthesis started with the reaction of 4-nitrobenzoyl chloride 490 with l-glutamic acid 491, to afford *N*-(4-nitrobenzoyl)glutamic acid 492. The nitro group of compound 490 was hydrogenated to an amino group in the presence of RANEY® that afforded *N*-(4-aminobenzoyl)glutamic acid 493. The latter was subjected to reductive methylation using formaldehyde and hydrogen that gave *N*-(4-methylaminobenzoyl)glutamic acid 494.^512–515^ On the other hand, 2,4,6-triaminopyrimidine 497 was prepared readily by treating malonic acid dinitrile with guanidine. Compound 497 was nitrosylated by anhydrous nitrous acid to afford 2,4,6-triamino-5-nitrosopyrimidine 498, which was subsequently reduced using NaBH_4_ to afford 2,4,5,6-tetraaminopyrimidine 499. Upon treating of the latter with 1,2-dibromopropionic aldehyde, the product of attaching bromine to acrolein, 2-amino-4-hydroxy-6-bromomethyl-pteridine 501 was obtained. In final step, the nitrogen atom of *N*-(4-methylaminbenzoyl)glutamic acid 494 was alkylated with the already prepared, bromide 501 to afford the desired target methotrexate 502 (Scheme 70).^516–523^

**Scheme 71:**
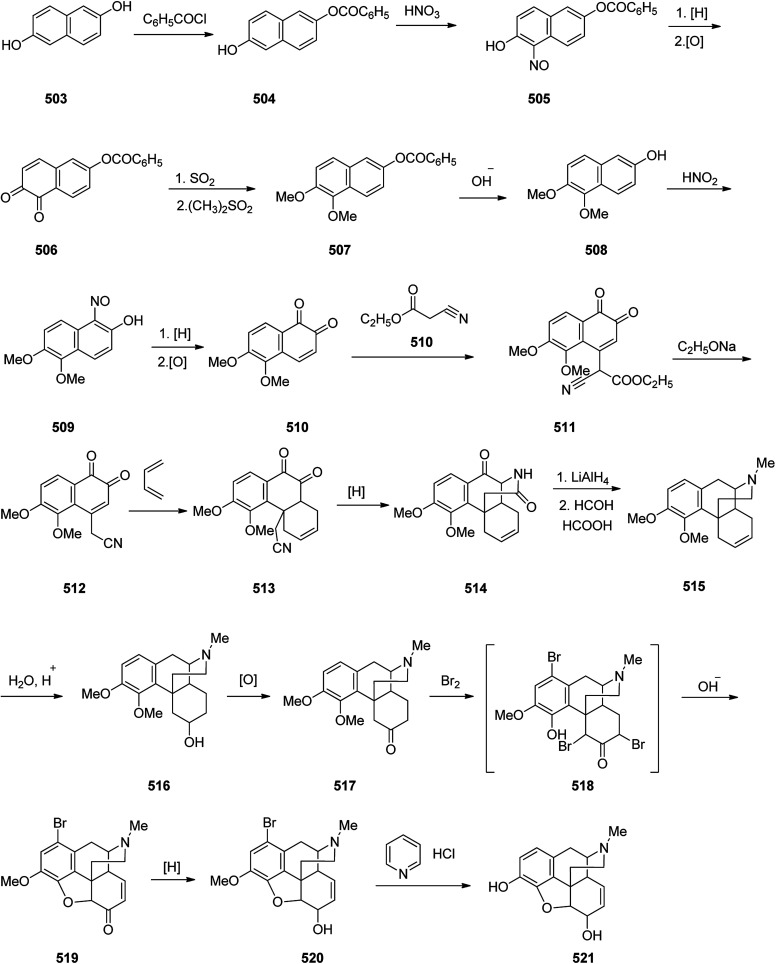
Synthesis of morphine 521.

A notorious morphine 522 as a legal medication is actually a strong pain killer.^524^ Morphine was initially isolated from the unripe seed pods of opium poppy, Papaver somniferum.^525–527^ Sertürner, who first isolated this compound, originally named it morphium.^528,529^ The isolation morphine as a naturally occurring compound is believed to be the first classical isolation of an active ingredient from a plant.^530^ A number of structurally related alkaloids, including codeine, thebaine, and codeinone, have also been isolated from the same plant.^531^ Morphine, chemically is 4,5-epoxy-17-methymorphin-7-ene-3,6-diol 522. One of the suggested, delicate, multi-phase approaches to the morphine synthesis is depicted in Scheme 71. The suggested pathway, started morphine 522 is synthesized from 2,6-dioxynaphthelene 503 that is reacted with benzoyl chloride to give monobenzoate 504, which upon further treatment with nitrous acid is transformed into 1-nitroso compound 505. Then, the latter was hydrogenated over a Pd catalyst and the resultant was subjected to further soft oxidation by iron trichloride to afford 6-benzoyloxy-1,2-naphthoquinone 506. The latter was reduced to 6-benzoyloxy-1,2,-naphthohydroquinone that is methylated using dimethylsulfate as methylating agent to afford 5,6-dimethoxy-2-benzoate 507. The latter next underwent alkaline hydrolysis to give 5,6-dimethoxy-2-naphthol 508. The last was subjected to the same consecutive steps of synthesis, involving nitrozation, reduction and oxidation (using the same reagents, as above), 5,6-dimethoxy-1,2-napthoquinone 510 obtained. The latter was then condensed with ethyl cyanoacetate through Knoevenagel reaction, using potassium ferrocyanide, for the oxidation of the condensation product, gave rise to the formation of product 512 which upon hydrolysis and further decarboxylated gave 5,6-dimethoxy-4-cyanomethyl-1,2-naphthoquinone 513. The latter was subjected to 4 + 2 cycloaddition reaction with 1,3-butadiene to afford a modest yield of 3,4-dimethoxy-9,10-dioxy-13-cyanomethyl-5,8,9,10,13,14-hexahy-drophenanthrene 514. The latter was then hydrogenated, in the presence of a copper chromite catalyst resulted in the formation of ketolactam 515. The last was reduced using lithium aluminum hydride, resulting in the reduction of the both carbonyl groups and amide, followed by methylation of the secondary nitrogen atom using a mixture of formaldehyde and formic acid gave racemic methyl ester β-Δ^6^-dihydrodesoxycodeine 516. Then, the resulting product was treated with l-(+)-dibenzoyltartaric acid afforded the (+)-methyl ester of β-Δ^6^-dihydrodesoxycodeine. This submitted to hydration using hot, dilute sulfuric acid, to afford the methyl ester of β-dihydrothebainol 517. The latter was treated vigorously with KOH in diethyleneglycol in which partial demethylation to β-dihydrothe-bainol took place followed by the oxidation of which in potassium *tert*-butylate/benzophenone system afforded β-dihydrothebainone 518. The latter was subjected to further bromination using 3 mol of bromine in HAOc gave (−)-1-bromocodeinone 520 that is isolated in the form of 2,4-dinitrophenylhyrazone. Apparently, in this step a double bond between both C7–C8 and an oxide bridge between C4–C5 concurrently is formed. In addition, an epimerization occurs at C14, *i.e.* the isomorphinan system isomerizes into a morphinane system. Subsequently (−)-1-bromocodeinone 520 was reduced by lithium aluminum hydride (LiAlH_4_) afforded codeine 521 that upon demethylation by pyridine hydrochloride produced the desired morphine 522 (Scheme 71).^532,533^

### 2.3. Seven-membered heterocycles

**Scheme 72:**
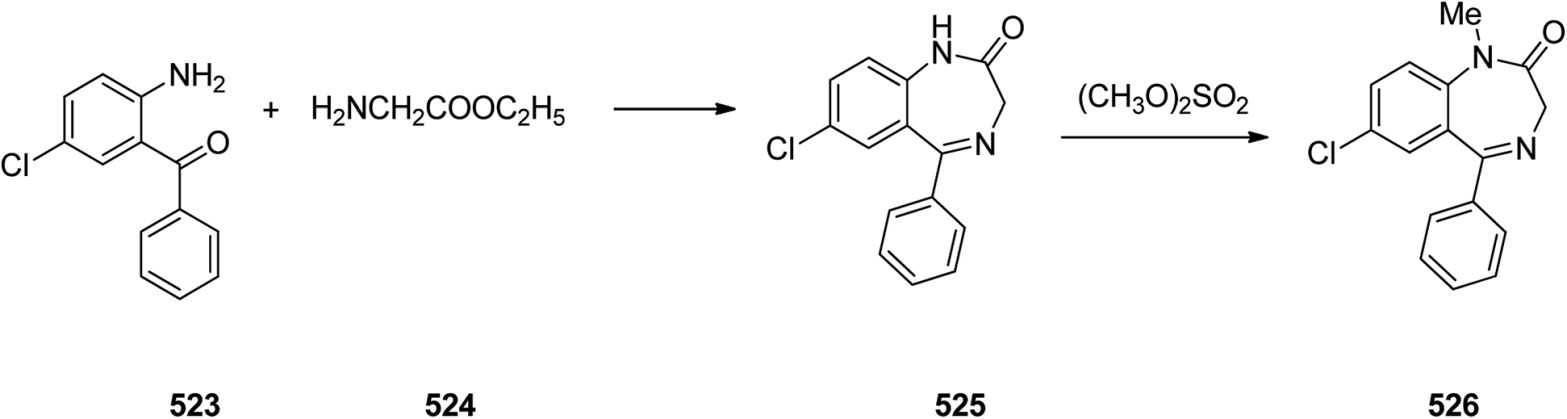
Synthesis of diazepam 526.

Diazepam 526, was synthesized and patented in 1959^534,535^ and marketed and well-known as Valium. It is classified in the benzodiazepine family that stereotypically provides a calming effect.^534^ It is frequently prescribed for the treatment of wide a range of conditions, involving anxiety, muscle spasms and trouble sleeping.^534^ Structurally, diazepam, is actually 7-chloro-1,3-dihydro-1-methyl-5-phenyl-2*H*-1,4-benzodiazepin-2-one 526. As a matter of fact, it is the most simple of all among the biologically potent derivatives of 1,4-benzodiazepin-2-ones. Diazepam can be synthesized stating from 2-amino-5-chlorobenzophenone *via* different approaches. Couple of ways involve of the direct cyclocondensation of 2-amino-5-chlorobenzophenone or 2-methylamino-5-chlorobenzophenone upon treatment with the ethyl ester of glycine hydrochloride to afford 7-chloro-1,3-dihydro-5-phenyl-2*H*-1,4-benzodiazepin-2-one 525. The amide nitrogen atom of 525 is methylated using dimethylsulfate results in the formation of diazepam 526 (Scheme 72).

**Scheme 73:**
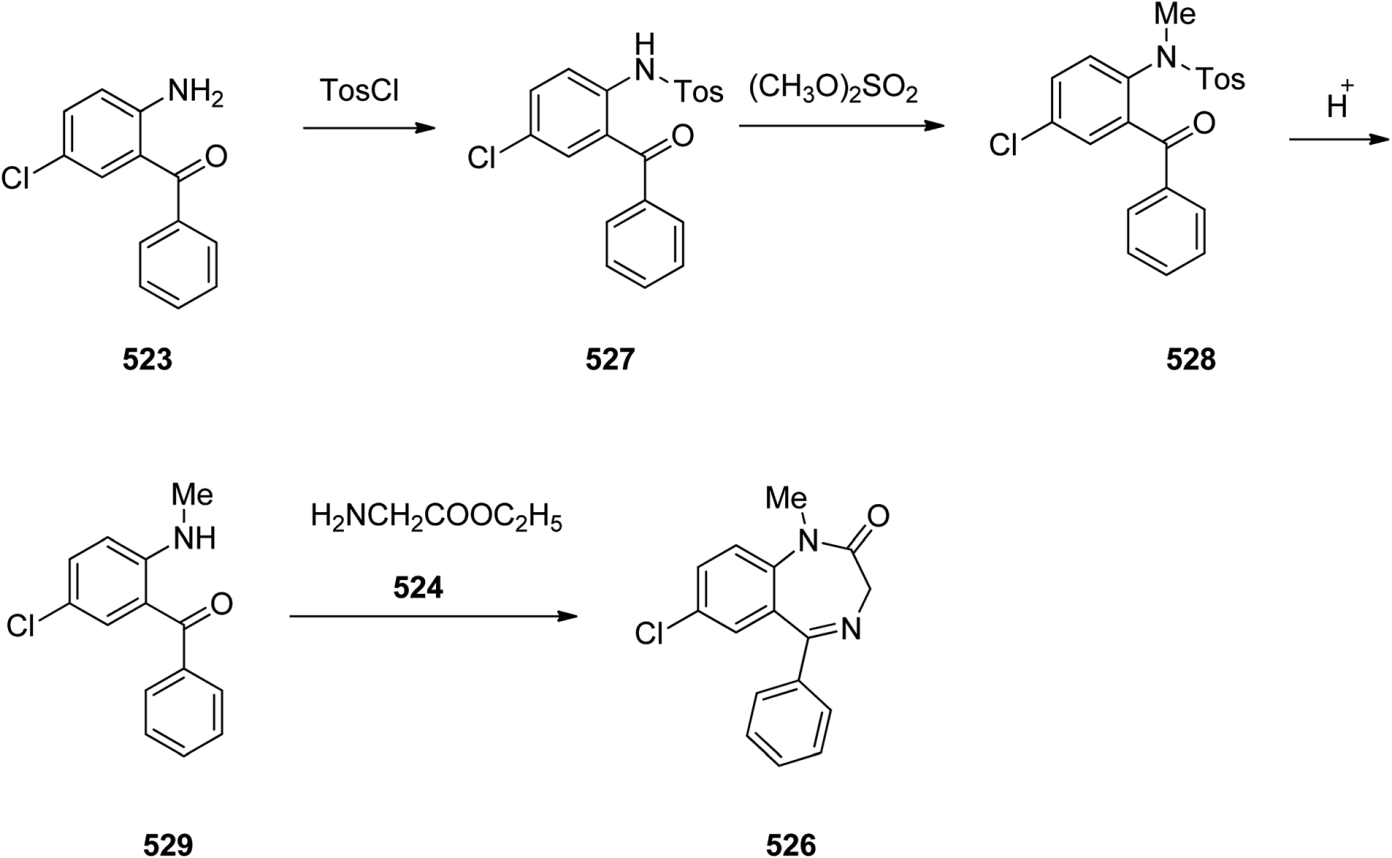
Synthesis of diazepam 526.

In the second approach, at first and before the cyclocondensation reaction, the nitrogen atom is methylated. Thus at first 2-amino-5-chlorobenzo-phenone is tosylated using *p*-toluenesulfonylchloride and the resultant tosylate 527 is converted to its *N*-sodium salt that is then methylated by dimethylsulfate to provide 2-*N*-tosyl-*N*-methyl-5-chlorobenzophenone 528. The latter is then hydrolyzed in an acidic medium, affording 2-methylamino-5-chlorobenzophenone 529 that is subjected to cyclocondensation *via* reaction with ethyl ester of glycine hydrochloride to afford the desired diazepam 526 (Scheme 73).^536–540^

**Scheme 74:**
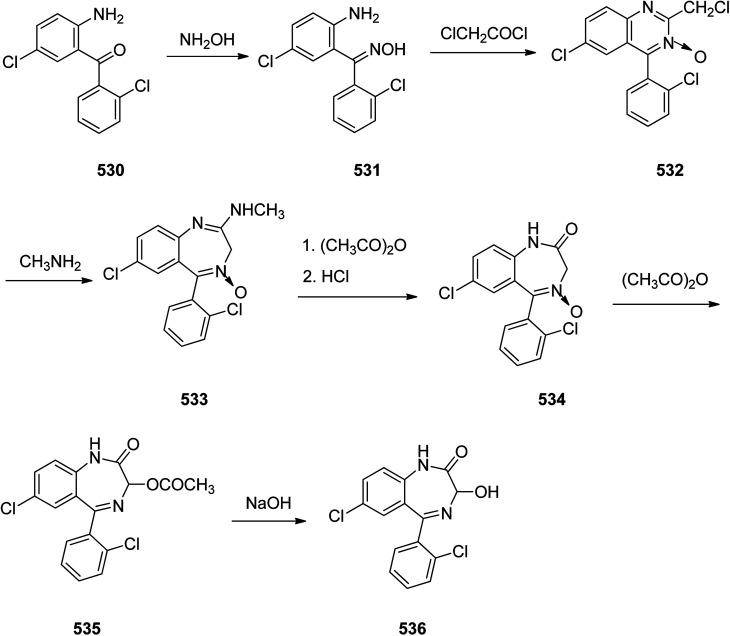
Synthesis of lorazepam 536.

Lorazepam 536, is also a benzodiazepine medicine. It is prescribed to treat anxiety disorders, trouble sleeping, active seizures including status epilepticus, alcohol withdrawal syndrome, and chemotherapy-induced nausea and vomiting. Lorazepam was first patented in 1963 and approved for being used as medication in 1977,^541^ sold under the brand name Ativan among others. Lorazepam, chemically is 7-chloro-4-(*o*-chlorophenyl)-1,3-dihydro-3-hydroxy-2*H*-1,4-benodiazepin-2-one 536. It is prepared in six steps started with 2-amino-2′,5-dichlorobenzophenone 530 which reacted with hydroxylamine to afford 531. The last was then reacted with chloracetyl chloride to afford 6-chloro-2-chlormethyl-4-(2′-chlorophenyl)quinazolin-3-oxide 532, upon heterocyclizationto. The latter upon reaction with methylamine, as in the case of chlordiazepoxide, resulted in rearrangement and a ring expansion, providing 7-chloro-2-methylamino-5-(2′-chlorphenyl)-3*H*-1,4-benzodiazepin-4-oxide 533. The last underwent acetylation at the secondary nitrogen atom, using Ac_2_O followed by hydrolysis in the presence of HCl gave 7-chloro-5-(2′-chlorophenyl)-1,2-dihydro-3*H*-1,4-benzodiazepin-2-on-4-oxide 534. Treatment of the latter with Ac_2_O resulted in a Polonovski type rearrangement, affording a 3-acetoxylated benzodiazepine, 7-chloro-1,3-dihydro-3-acetoxy-5-(2′-chlorphenyl)-2*H*-benzodiazepin-2-one 535, which upon hydrolysis produced the desired product lorazepam 536 (Scheme 74).^542–546^

**Scheme 75:**
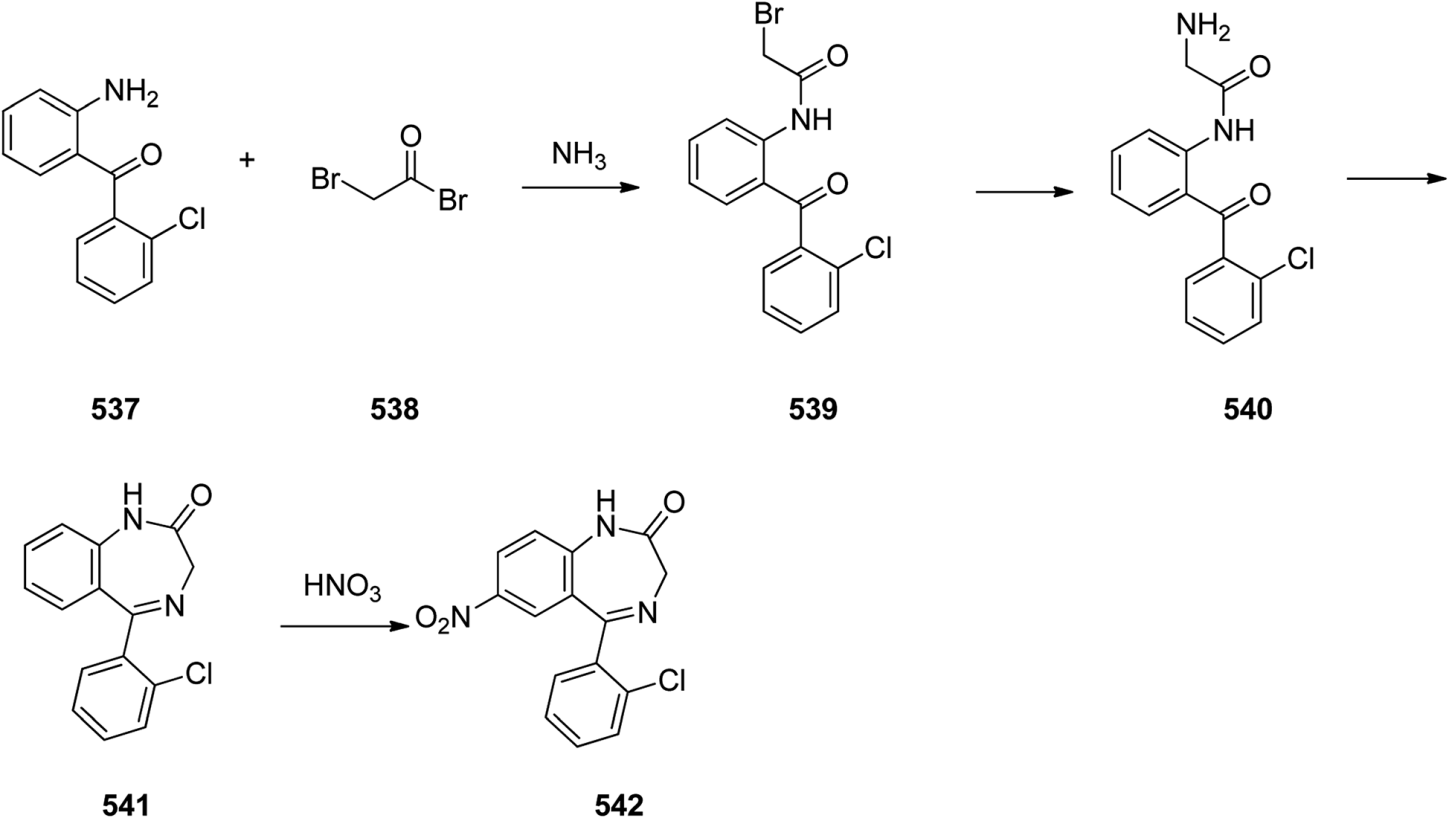
Synthesis of clonazepam 541.

Clonazepam 542 was synthesized and patented in 1960 but approved by FDA in 1975.^547^ It is also sold under the trade name, Klonopin among others. It is a medication to prevent and treat seizures, panic disorder, and the movement disorder known as akathisia and as recreational drug. Clonazepam,^548^ which chemically is 5-(2-chlorphenyl)-1,3-dihydro-7-nitro-2*H*-1,4-benzodi-azepine-2-one 542 was produced in five steps starting from 2-chloro-2′nitrobenzophenon that was hydrogenated in the presence of RANEY® to afford 2-chloro-2-aminobenzophenon 537. The amino group of the latter was amidated using 2-bromoacetyl bromide to afford the bromacetamide 539 which was next converted into aminoacetamide 540 through the reaction with ammonia. Heating of the latter in pyridine as a basic solvent with pyridine, resulted in intramolecular cyclization to provide 5-(2-chlorophenyl)-2,3-dihydro-1*H*-1,4-benzodiazepine-2-one 541. The nitration of the latter in mild reaction conditions (KNO_3_ in H_2_SO_4_) led to the production of clonazepam 542 (Scheme 75).^549–554^

**Scheme 76:**
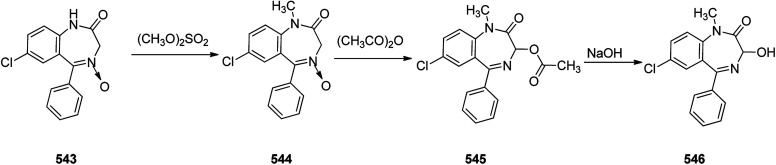
Synthesis of temazepam 546.

Temazepam 546, is accessible as a generic medication^555^ and was patented in 1962 whereas approved for being prescribed in 1969.^556^ It came to market under the brand names Restoril among others. It is a medication used to treat trouble sleeping and is an intermediate acting benzodiazepine and hypnotic.^557^ Temazepam, chemically is 7-chloro-1,3-dihydro-3-hydroxy-1-methyl-5-phenyl-2*H*-1,4-benzodiazepin-2-one 546. It is produced in three steps from one of the intermediates in oxazepam synthesis, 7-chloro-5-phenyl-1,3-dihydro-3-hydroxy-1-methyl-5-phenyl-2*H*-1,4-benzodiazepin-2-on-4-oxide 543. The latter was methylated at the nitrogen of the amide group in the first position of the benzodiazepine ring by dimethylsulfate as methylating agent that afforded 1-methyl-7-chloro-5-phenyl-1,3-dihydro-2*H*-1,4-benzodiazepin-2-on-4-oxide 544. The latter was then underwent acetylation using acetic anhydride, affording 1-methyl-3-acetoxy-7-chloro-5-phenyl-1,3-dihydro-2*H*-1,4-benzodiazepin-2-one 545. This transformation is believed to proceed *via* Polonovski reaction. The acetyl group in the resulted compound 545 was removed *via* alkaline hydrolysis (NaOH) resulted in the formation of the desired temazepam 546 (Scheme 76).^558–563^

**Scheme 77:**
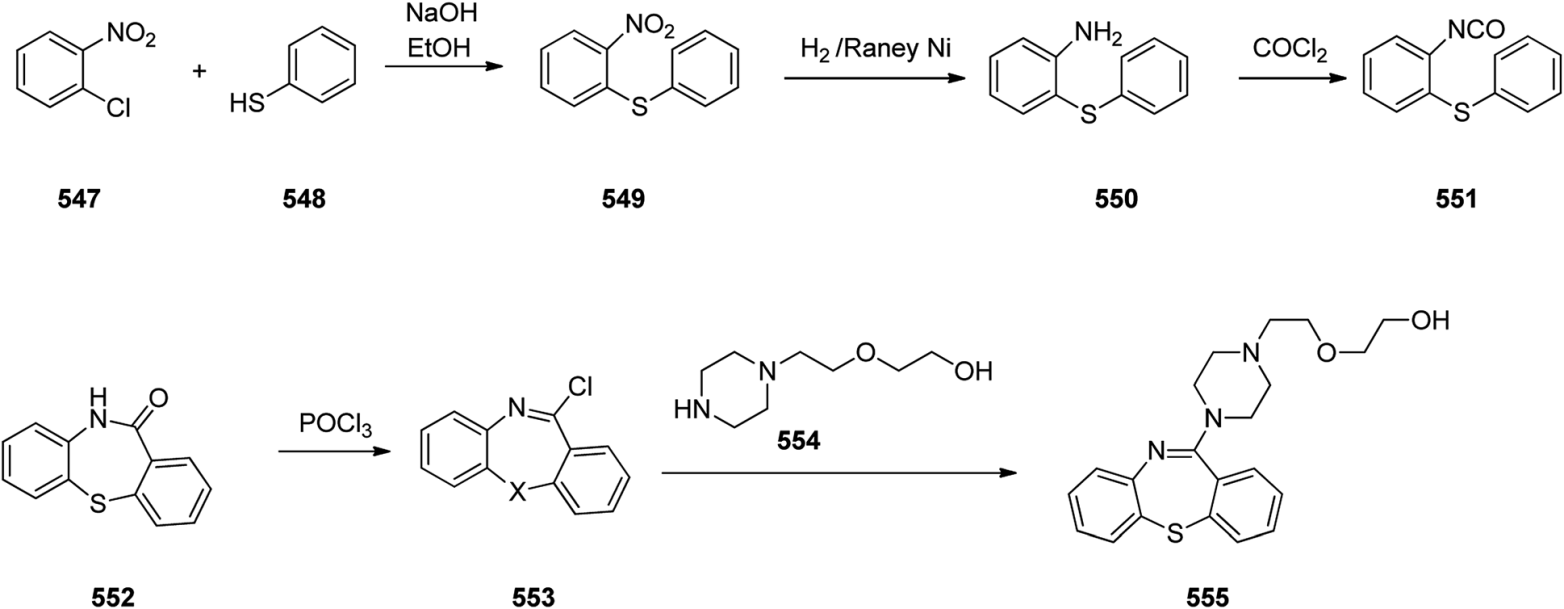
Synthesis of quetiapine 555.

Olanzapine 552, which came to market under the brand name Zyprexa, is a typical antipsychotic. It was commercialized under the brand name Seroquel among others. It is used for the treatment of schizophrenia and bipolar disorder. Olanzapine was patented in 1971 and approved by FDA in 1996 for being prescribed.^564^ It shows a relatively wide range receptor binding profiles. It exhibits moderate affinity to α_1_- und α_2_-adrenergic receptors and slight affinity to muscarinergic M1 receptors.^565^ The multistep synthesis of quetiapine 555 was started from the reaction of *o*-chloronitrobenzene 547 with thiophenol 548 in the presence of NaOH in EtOH to afford the corresponding *o*-nitrodiphenyl sulfide 549. The latter was reduced by hydrogenation catalyzed by RANEY® to give the corresponding amine 550. The latter was reacted with phosgene to provide isocyanate 551, which upon heating in the presence of AlCl_3_ in *o*-dichlorobenzene as solvent to give a key intermediate dibenzo[*b*,*f*][1,4]thiazepine-11(10*H*)-one 552.^566^ The latter upon heating in POCl_3_, and dimethylaniline afforded the intermediate iminochloride 553, which was reacted with 2-(2-(piperazin-1-yl)ethoxy)ethanol 554 to provide the final desired product, quetiapine 555 (Scheme 77).^567,568^

**Scheme 78:**
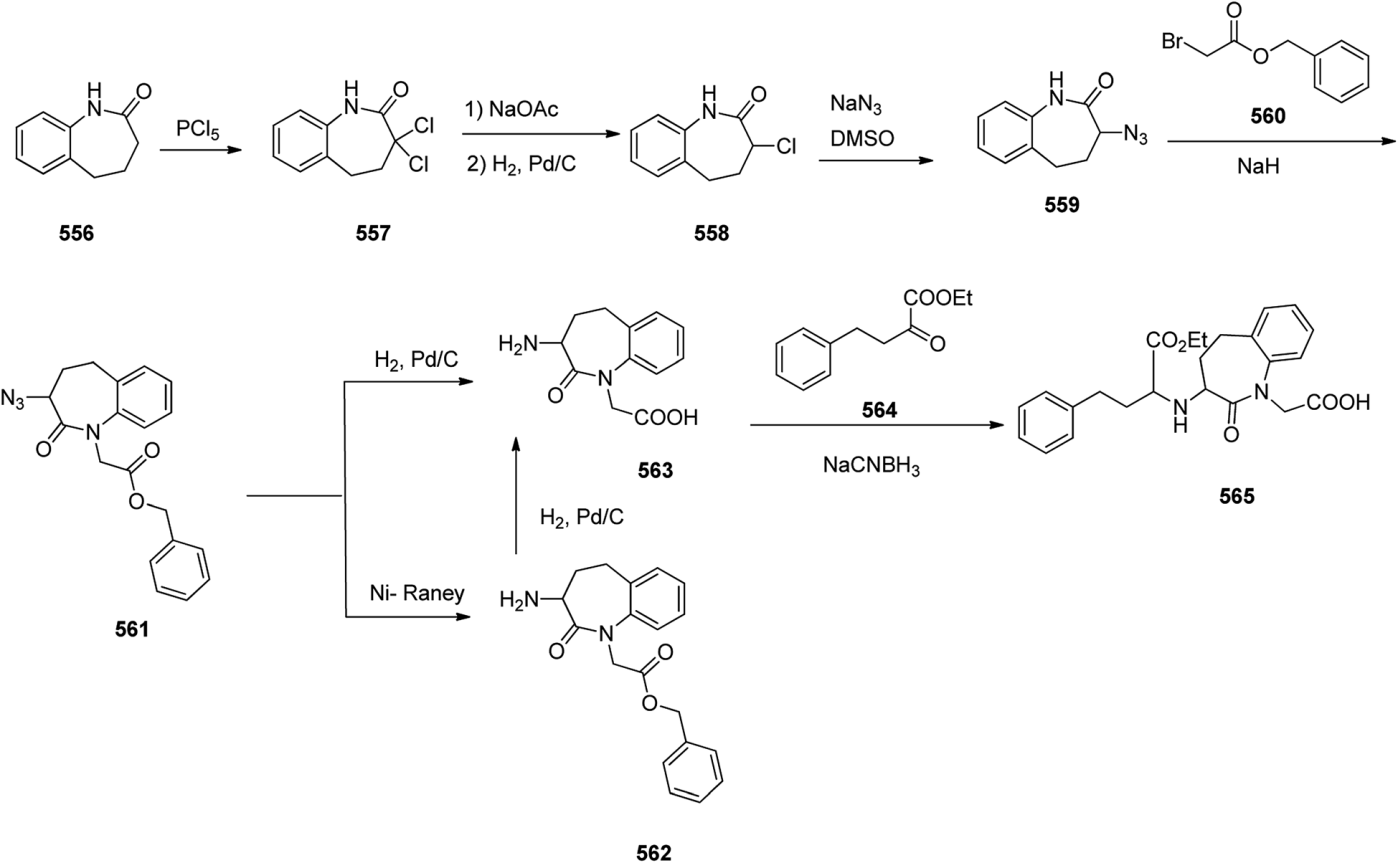
Synthesis of benazepril 565.

Benazepril 565, is accessible as a generic medication prescribed for the treatment, hypertension, chronic renal failures, and diabetic kidney disease. It was patented in 1981 and came into market in 1990, sold under the brand name Lotensin among others.^569^ In 2003 Chang and coworkers^570^ demonstrated an enantioselective synthesis of a benazepril intermediate through a bioreductive reaction using baker's yeast, but the enantioselectivity was not ideal for a medication (80% ee). Benazepril 565, was prepared *via* a multistep involving the treatment of 2,3,4,5-tetrahydro-1*H*-(1) benzazepin-2-one 556 with PCl_5_ in hot xylene afforded 3,3-dichloro-2,3,4,5-tetrahydro-1*H*-(1)benzazepin-2-one 557, which is initially treated with NaOAC and hydrogenated over Pd/C in CH_3_COOH providing 3-chloro-2,3,4,5-tetrahydro-1*H*-(1)benzazepin-2-one 558. The latter was reacted with NaN_3_ in DMSO gave 3-azido-2,3,4,5-tetrahydro-1*H*-(1)benzazepin-2-one 559, which is then condensed with benzyl bromoacetate 560 in the presence of NaH in DMF affording 3-azido-1-(benzyloxycarbonylmethyl)-2,3,4,5-tetrahydro-1*H*-(1)benzazepin-2-one 561. The latter was treated with RANEY® in EtOH/water to give 3-amino-1-(benzyloxycarbonylmethyl)-2,3,4,5-tetrahydro-1*H*-(1)benzazepin-2-one 562, which was debenzylated upon hydrogenation with H_2_ over Pd/C in EtOH to furnish 3-amino-1-(carboxymethyl)-2,3,4,5-tetrahydro-1*H*-(1)benzazepin-2-one 563. At last, compound 563 was condensed with ethyl 3-benzylpyruvate 564 in the presence of sodium cyanoborohydride in MeOH/AcOH to give the desired medication, benazepril 565 (Scheme 78).^571^

**Scheme 79:**
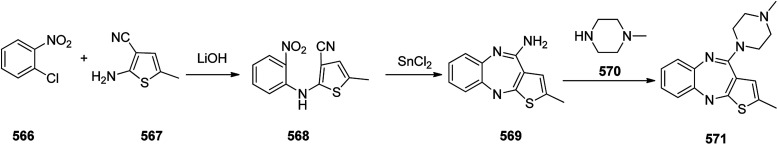
Synthesis of olanzapine 571.

Olanzapine 571, which came to market under the brand name Zyprexa among other trade names, is a typical antipsychotic. It is used for the treatment of schizophrenia and bipolar disorder. Olanzapine was patented in 1971 and approved by FDA in 1996 for being prescribed.^572^ The synthetic pathway of olanzapine 571 is illustrated in Scheme 79. 2-Amino-5-methylthiophene-3-carbonitrile 567 was reacted with *o*-chloronitrobenzene 566 to provide 2-phenylamino-thiophene-3-carbonitrile derivative 568. The nitro group of the latter was reduced to the amino group using SnCl_2_ which is concurrently cyclized to give 4-amino-2-methyl-10*H*-thieno[2,3-*b*][1,5]benzodiazepine 569. The latter was reacted with *N*-methylpiperazine 570 to afford the desired amidine-olanzapine 571.^573,574^

## 3. Conclusion

Due to their inherent resourcefulness and versatility as well as exceptional physicochemical potencies, heterocyclic systems have dignified themselves as factual cornerstones of medicinal chemistry. The main stream of heterocyclic systems and archetypally common heterocycle scaffolds are present in most natural products and medications which are currently prescribed thus, market purchasable. Among them, nitrogen heterocycles are imposing since by quick glance at FDA databases their structural significance is unveiled. In the FDA list of approved drugs approximately 60% are nitrogen-based heterocycles, thus these heterocyclic systems are important in the drug design, drug discovery and engineering of medications. In this review, we tried to underscore the top and best-selling prescribed drugs, containing N-heterocyclic systems. Thus, in this review, we classified the N-heterocyle medications in accordance with their sizes. In addition, we tried to give the readers some elementary information about their biological and clinical applications. Furthermore, the selected synthetic pathways towards the production of those drugs as published in both chemical literatures and patents, were underlined. We wish this review attracts the attention of organic synthetic chemists, as well as biologists, pharmacists and general practitioners and specialists.

## Conflicts of interest

There is no conflict of interest.

## Supplementary Material
